# Exploration of the Shared Gene Signatures between Myocardium and Blood in Sepsis: Evidence from Bioinformatics Analysis

**DOI:** 10.1155/2022/3690893

**Published:** 2022-08-06

**Authors:** Qi Long, Gang Li, Qiufen Dong, Min Wang, Jing Li, Liulin Wang

**Affiliations:** ^1^Department of Critical Care Medicine, Hubei Province Hospital of Traditonal Chinese Medicine, 856 Luoyu Street, Wuhan, Hubei 430061, China; ^2^Hubei Province Academy of Traditonal Chinese Medicine, 856 Luoyu Street, Wuhan, Hubei 430061, China

## Abstract

**Background:**

Septic cardiomyopathy is widespread during sepsis and has adverse effects on mortality. Diagnosis of septic cardiomyopathy now mainly depends on transthoracic echocardiogram. Although some laboratory tests such as troponin T and atrial brain natriuretic peptide play a role in the diagnosis, specific blood biochemistry biomarkers are still lacking. *Objective and Methods.* In our study, we sought to find potential biological markers from genes and pathways that are covariant in the blood and myocardium of septic patients. Bioinformatics and machine learning methods were applied to achieve our goal. Datasets of myocardium and peripheral blood of patients with sepsis were obtained from the Gene Expression Omnibus (GEO) database. Differentially expressed genes (DEGs) were selected and received functional enrichment analysis. Unsupervised hierarchical clustering analysis was performed to identify the subtypes of sepsis. Random forest, lasso regression, and logistic regression were used for variable screening and model construction. Internal and external validation sets were applied to verify the efficiency of the model in classifying disease and predicting mortality.

**Results:**

By defining significance for genes using Student's *t*-test, we obtained 1,049 genes commonly changed in both myocardium and blood of patients with sepsis. The upregulated genes (LogFC >0) were related to inflammation pathways, and downregulated (LogFC <0) genes were related to mitochondrial and aerobic metabolism. We divided 468 sepsis patients into two groups with different clinical result based on the mortality-related commonly changed genes (104 genes), using unsupervised hierarchical clustering analysis. In our validation datasets, a six-gene model (*SMU1*, *CLIC3*, *SP100*, *ARHGAP25*, *DECR1*, and *TNS3*) was obtained and proven to perform well in classifying groups and predicting mortality.

**Conclusion:**

We have identified genes that have the potential to become biomarkers for septic cardiomyopathy. Additionally, the pathophysiological changes in the myocardium of patients with sepsis were also reflected in peripheral blood to some extent. The co-occurring pathological processes can affect the prognosis of sepsis.

## 1. Introduction

Septic shock is often characterized by refractory hypotension and insensitivity to vasoactive agents, especially when the hypovolemia is redressed. The pathogenesis of septic shock has been extensively and profoundly discussed, with septic myocardial injury or septic cardiomyopathy (SCM) being considered an important cause of refractory hypotension caused by septic shock. The presence of SCM in septic shock, as determined by previous studies, infers a poorer prognosis as it significantly increases the mortality rate of patients to 70–90% and its incidence varies from 18 to 40% of septic shock patients [1, 2]. To date, the complex mechanism of SCM pathogenesis remains unclear. Myocardium depressant substances may be one of the most important factors. The most primary myocardial depressant factors include substances such as cytokines, prostanoids, nitric oxide, the complement system, and lipopolysaccharides (LPS), which are involved in actively suppressing the heart [3]. Exosome and dysfunction of mitochondrial dysfunction also play important roles in the pathogenesis of SCM [4–7].

Currently, the presence of SCM can be diagnosed in patients using a bedside transthoracic echocardiogram, which typically shows left ventricular ejection fraction <45% and right ventricular dilatation [8]. Some laboratory tests, including Troponin (cTnI) and brain natriuretic peptide (BNP), play auxiliary roles in the diagnosis of this disease, but specificity is still lacking. The prognostic value of these biomarkers seems more closely tied to disease severity than to specific abnormalities in cardiac function [9–12]. Considering the problems we have faced in our clinical work, we studied those genes and pathways that change synchronously in the blood and myocardium in order to facilitate our access to potential biomarkers.

Gene expression profiling methods have been widely used in many fields of clinical and scientific research [13–15], and the research on sepsis genomics has been gradually carried out [16–20]. Many sequencing data obtained from peripheral blood indicate the changes in inflammation-related genes and pathways in sepsis patients at different stages and outcomes. In addition, a recent study sequenced the myocardial tissue of patients with sepsis and found that mitochondrial-related proteins and sarcomere proteins were extensively downregulated in the myocardial tissue of patients with sepsis compared to the control group [16]. Although myocardial depressant factors and dysfunction of mitochondria are common in sepsis, we do not know the correlation of these pathologic processes in myocardium and peripheral blood, nor the details about their regulatory direction. Therefore, we believe that comparing the genetic and cellular pathways that covary in the blood and myocardium of patients with sepsis may help us identify some meaningful biomarkers that are specific for septic cardiomyopathy diagnosis, particularly regarding inflammatory response and mitochondrial function.

Machine learning methods have been widely used in the identification and establishment of sepsis subtypes, but most findings are mainly aimed at mortality-related genes, and the classification characteristics obtained are mainly different immune and inflammatory response states [17, 18, 20]. In our study, we looked at genes that changed in both the myocardium and peripheral blood of patients with sepsis, which included some mitochondrial and aerobic metabolism-related genes, as the variables of clustering analysis, and tried to identify the population in sepsis with potential myocardial injury through the changes in the peripheral blood. We then used machine learning methods and model construction to screen variables, build a prediction model with those genes that are representative, and verify their efficiency in group classification and mortality prediction.

## 2. Materials and Methods

### 2.1. Gene Expression Profiles and Data Preparation

**Figure 1:**
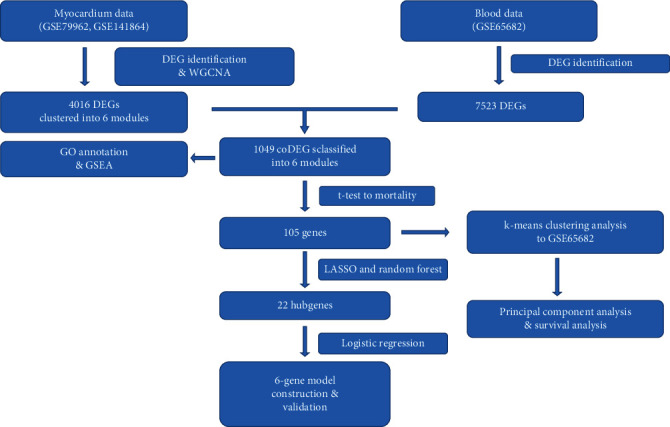
Pipeline of our proposed work and statistical analysis methods.

The main procedure of our statistical analysis is displayed in Figure 1. There are 51 myocardium samples in GSE79962, including 20 septic cardiomyopathy (SCM), 11 ischemic heart disease (IHD), 11 dilated cardiomyopathy (DCM), and nine nonfailing heart (NFH) samples. GSE141864 included eight septic cardiomyopathy samples and two nonfailing heart samples.

We collected 802 whole blood samples for sepsis patients (*n* =760) and healthy controls (*n* =142) in the blood dataset GSE65682. In total, 468 sepsis samples with 28 days of survival data were studied as training datasets and internal testing datasets for model construction with sepsis survivors (78.0%, 365/468) and sepsis nonsurvivors (22%, 103/468).

Four external validation datasets were obtained in our study. GSE54514 contained 163 daily *PAX* gene samples for up to five days for sepsis survivors (*n* =26), sepsis nonsurvivors (*n* =9), and healthy controls (*n* =18). GSE131761 contained 129 gene samples for nonseptic shock (*n* =33), septic shock (*n* =81), and healthy controls (*n* =15). GSE57065 contained 107 blood samples for 40 simplified acute physiology score II- (SAPS II-) high septic patients, 42 SAPS II-low septic patients, and 25 controls. GSE110487 contained 62 blood samples from 31 septic patients who received early liquid resuscitation therapy, 17 of them were responders and 14 were not responders. For each patient, two samples were collected. In particular, the first sample (T1) collected within 16 hours from ICU admission whereas the second (T2) collected within 48 hours from ICU admission.

All raw data were preprocessed using the affy package in R [22]. We integrated the myocardial tissue data from GSE79962 and GSE141864 and used the sva package in R to remove the batch effect [22]. We then used the psych package in R to perform principle component analysis (PCA) of the dataset and used the first two principal components to draw a scatter plot to observe the distribution of the samples [23]. We found that the sepsis samples in the dataset were well fused and isolated from the other diseases and control samples. In the GSE79962 study, the authors collected IHD and DCM groups to eliminate interference caused by other kinds of heart failure. They compared IHD, DCM, and the sepsis group to NDH separately and visualized three groups of differentially expressed genes (DEGs) by Venn diagram. The DEGs in sepsis were discarded when they appeared in DEGs of IHD and DCM. We used this principle in our research and finished the work using the weighted correlation network analysis (WGCNA) R package. If the modules generated by WGCNA showed no statistically significant correlation with sepsis group, the DEGs in this module were dropped.

Other blood sample databases were analyzed separately. GSE65682 served as a deviation dataset, which was divided into the training set and the internal validation set, and the rest of the datasets served as the external validation set.

### 2.2. DEG Analysis and WGCNA

In the myocardium dataset (combination of GSE79962 and GSE141864) and peripheral blood dataset (GSE65682), DEGs analysis was performed separately using the limma package in R [24]. Gene significance was defined using a Student *t*-test statistic for testing differential expression between sepsis and controls. Every *p*-value was first adjusted by the Benjamini & Hochberg method. In GSE65682, we compared the sepsis group and healthy controls, and set the adjusted *p*-value <0.05 as the cut-off for DEGs selection.

In the myocardium dataset, we first compared the sepsis group and nondilated heart. In order to obtain more potentially significant genes, we tolerated more possible errors at the beginning and set *p*-value <0.05 as criterion for DEGs selection. More specific selection was done following WGCNA.

The log fold change (LFC) value was calculated by subtracting the average control value from the average sepsis value for each gene. Using LFC >0 or <0, the DEGs were classified as upregulated or downregulated.

In the GSE79962 study, the authors compared the septic cardiomyopathy, dilated cardiomyopathy, and ischemic heart disease separately to the nonfailing heart and obtained three different groups of DEGs. Co-expressed DEGs among the three groups were abnegated, in order to eliminate the effects caused by other types of heart failure [16]. We applied this principle and finished the course with WGCNA. We first obtained the DEGs between septic group and nonfailing heart with *p*-value <0.05, which were applied for WGCNA [25]. Finally, modules were identified in the resulting dendrogram using the Dynamic Tree Cut algorithm. Modules with similar expression profiles were merged at the threshold of 0.25. While the modules were confirmed, we tested the correlation between modules and gene significance using Student's *t*-test. Genes in the module that were confirmed to be correlated with gene significance were kept for further analysis. While separated by different modules, the DEGs in the myocardium dataset were still marked as upregulated or downregulated.

We compared the DEGs from the myocardial dataset, which were grouped by different modules, to the DEGs from blood dataset. We retained genes that were both upregulated and downregulated in the blood dataset, and genes that changed in different directions were discarded. We then obtained the genes that changed in the same way in both blood and myocardium. In addition, we used the WGCNA classification of myocardial DEGs to classify these co-expressed genes into different modules.

### 2.3. Functional Enrichment of DEGs

The pathway information in GO was used to annotate the retained DEGs in each module and obtain the pathways of biological process, molecular function, and cellular component that would be dysregulated in both the blood and myocardium of septic shock patients. A *p*-value <0.05 and *q*-value <0.05 indicated a significantly enriched term. Then, GSEA was applied for further analysis of the pathway regulation, with an adjusted *p*-value <0.05 indicating a significantly enriched term. The R package clusterProfiler was used in these analyses [26].

### 2.4. Predictor Variable Selection

In the deviation dataset GSE65682, all the 1,049 remaining DEGs received univariate filtering on mortality. We used Student's *t*-test to compare the 1,049 DEGs between patients who survived and deceased patients by setting *p*-value <0.05. In total, 105 genes were verified to be related to mortality in the sepsis patient group. Compare to the external validation databases, 104 genes were used for variable selection, while one was unavailable in the external datasets. A recursive-partitioning machine learning algorithm, random forest, was used to identify the most important classifier variables in the derivation dataset. For variable selection, this technique is known to penalize categorical variables, particularly those with the fewest categories. Therefore, LASSO was also used to identify important classifier variables [27]. The random forest and glmnet packages in R were used for the courses [28, 29].

### 2.5. *K*-Means Clustering Analysis

The 104 genes were used for *k*-means clustering analysis in 468 septic patients from GSE65682. The number of clusters was determined by the elbow method and average silhouette method. Survival analysis was performed between the two groups. The NbClust package in R was used for the clustering [30].

### 2.6. Logistic Regression in Deviation Dataset

The GSE65682 dataset was divided into a training set and verification set in a ratio of 4: 1. The top genes obtained from the machine learning methods were used for backward stepwise regression to the clustering groups, and nested logistic regression models were generated by sequential deletion of the variables until all the variables in the model were statistically meaningful with a *p*-value <0.01. Chi-square test was used to compare the predictive efficiency of different models. Finally, six genes were kept in the regression model. The glmnet package in R was used for the analysis [28].

### 2.7. Model Performance in the Internal and External Verification Dataset

The gene model was first validated to predict the classification and mortality prediction efficiency of the test dataset generated in GSE65682 and compare it to age, using the AUC of the ROC curve. In GSE54514, we also used the same method to compare the efficiency of the six-gene model in predicting mortality with the age and appach II score. We also integrated the six genes with age and appach II score, and evaluated the weight of different factors in predicting mortality with a nomograph and calibration curve. The pROC, survival, and rms packages in R were used for analysis [31]. In GSE57065, GSE131761, and GSE110487, we verified the ability of the classification model to classify severe patients, identify septic shock and nonseptic shock, and judge the response to fluid therapy using a chi-square test.

## 3. Results

### 3.1. DEG Identification and WGCNA

**Figure 2:**
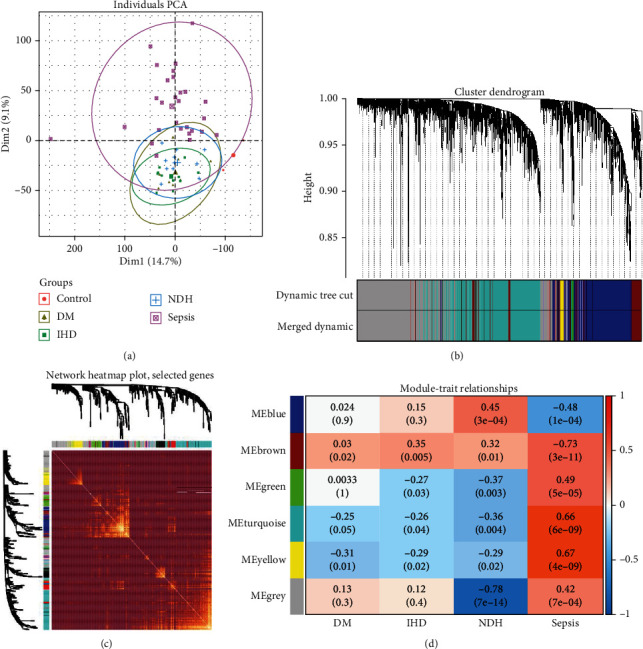
PCA and WGCNA analysis. (a) Principal component analysis of the myocardium dataset; samples were classified by the two most important principal components. The sepsis group is separated from other groups. (b) Dendrogram of all differentially expressed genes clustered based on the measurement of dissimilarity. A total of 11 modules were identified by clustering analysis. (c) Heat map plot shows the topological overlap matrix (TOM) among all genes and presents the correlation between different samples and modules after clustering analysis. (d) Correlation between the module eigengenes and different patient groups are presented in the heat map.

We merged the GSE79962 and GSE141864 together as a myocardium dataset. The new dataset included 61 myocardium samples for sepsis (*n* =28), IHD (*n* =11), DCM (*n* =11), and NFH (*n* =11). According to the result of PCA analysis, the sepsis data from GSE79962 and GSE141864 merged well and were separated from other groups (Figure 2(a)). Thus, we set the NFH group as the control. By using *p*-value <0.05 as the criterion, a total of 4,016 DEGs in the myocardium dataset were used for WGCNA. IHD and DCM groups were kept in WGCNA to filter the interference caused by other kinds of heart failure. Six modules were ultimately identified (Figures 2(b)–2(d)). By testing the correlation between modules and gene significance by using Student's *t*-test statistic (*p* < 0.001), blue, brown, grey, and turquoise modules were found to be correlated linearly with gene significance (Figure S1). There were 841 genes in the blue module, 422 genes in the brown module, 1,102 genes in the turquoise module, and 1,554 genes in the grey module.

In GSE65682, 760 sepsis patients and 142 healthy controls were obtained, with the sepsis group compared to healthy controls and every *p*-value adjusted by the Benjamini & Hochberg method. With the *p*-value <0.05 set as the cut-off point, we observed 7,523 DEGs in the blood dataset. By comparing 4,016 DEGs from the myocardium dataset with 7,523 DEGs from the blood dataset and correlating the LFC direction, 1,049 DEGs changed in the same way in both myocardium and blood datasets, including 549 genes upregulated and 500 genes downregulated. Moreover, the 1,049 DEGs were also marked with different modules, with 325 genes in the blue module, 116 genes in the brown module, 305 genes in the grey module, 261 genes in the turquoise module, 40 genes in the yellow module, and two genes in the green module (Table S1).

### 3.2. GO Annotation and GSEA

**Figure 3:**
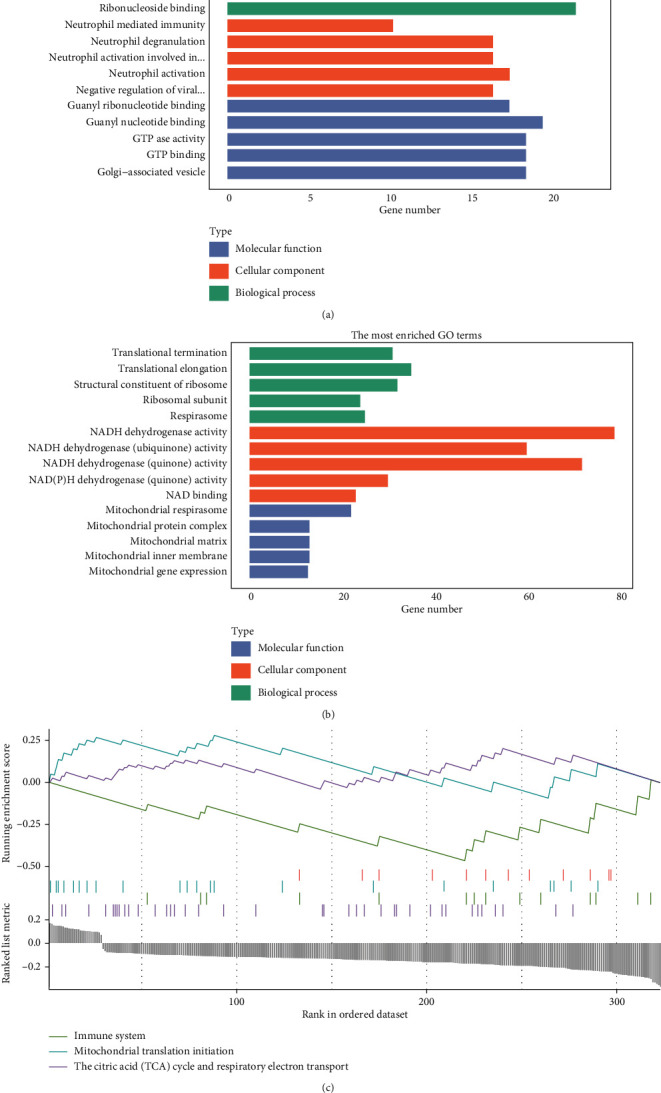
GO annotation. (a) GO annotation of upregulated hub genes in turquoise module. (b) GO annotation of downregulated hub genes in the blue module. (c) GSEA of downregulated hub genes in the blue module.

Using GO annotation, we observed that several cellular pathways and inflammatory cytokines were upregulated in the turquoise modules (Figure 3(a)). In the blue module, pathways for mitochondrial and protein complex disassembly were downregulated (Figure 3(b)). No meaningful pathways were identified in other modules. Detailed GO analysis results are shown in Table S2, with several pathways of mitochondrial and aerobic respiratory function being significantly enriched in the blue module (adjusted *p*-value <0.01). Pathways of the immune system, mitochondrial translation initiation, the citric acid (TCA) cycle, and respiratory electron transport were significantly enriched (adjusted *p*-value <0.05). No meaningful pathways were identified by GSEA in the turquoise module, with the results displayed in Figure 3(c) and Table S2.

### 3.3. *K*-Means Clustering Analysis

**Figure 4:**
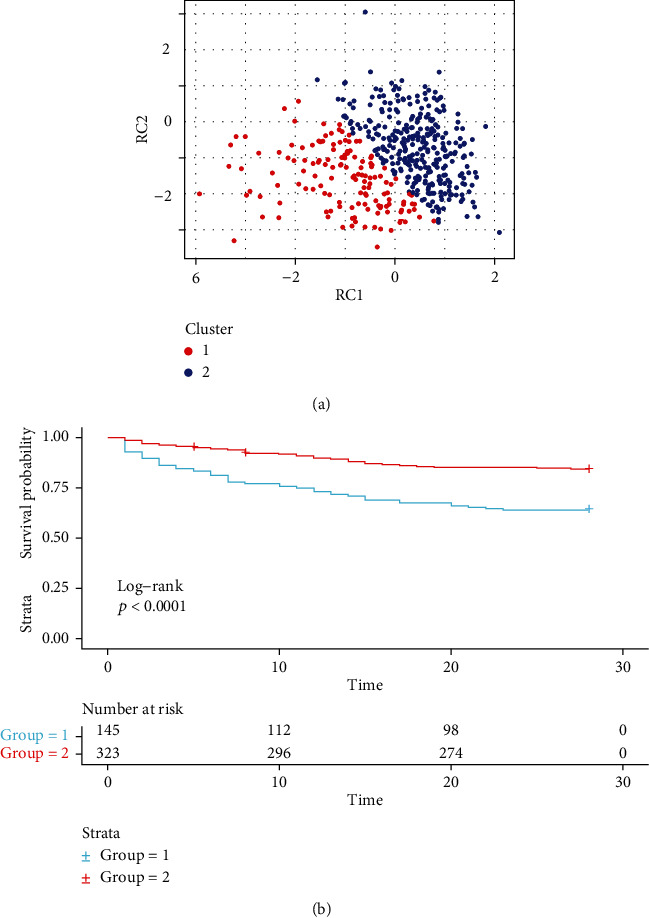
(a) Distribution of sepsis is presented in a scatter diagram, in which two classes of sepsis grouped by the clustering analysis are well separated by the two most important dimensions after PCA. (b) Survival analysis of two classes of sepsis shows that the survival rate of Class2 is lower than that of Class1, and it is statistically significant (*p* < 0.001).

A total of 105 genes were verified to be related to mortality in the sepsis patient group from 1,049 DEGs (Table-S3). Clustering analysis was performed in GSE65682 using the 105 DEGs obtained. The optimal number of clusters was determined to be two by measuring the total within sum of square and average silhouette width (Figure S2A). PCA was then performed, with patients well separated in the first two major dimensions (Figure 4(a), Figure S2A). There were 145 patients in Class 1 (31.0%) and 323 patients in Class 2 (69.0%). Patients in Class 1 exhibited significantly higher mortality rates than in Class 2 (35.9% [52/145] vs. 15.8% [51/323]; *p* <0.01 for chi-square test). The survival analysis was also statistically different (Figure 4(b)).

### 3.4. Development of Class Model

**Figure 5:**
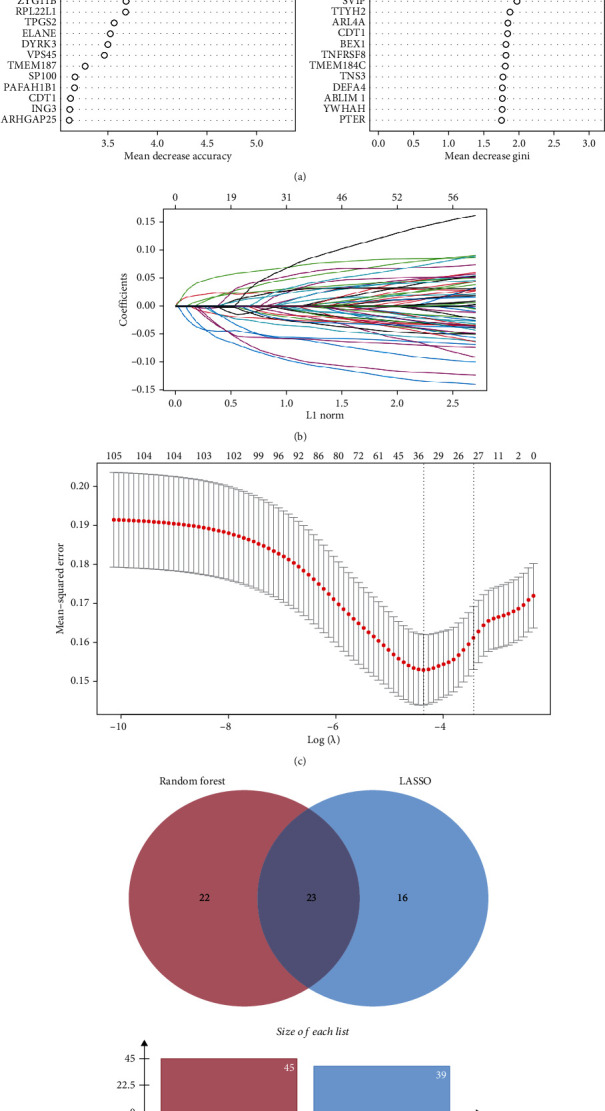
(a) Score of MeanDecreaseAccuracy and MeanDecreaseGini indicated the weight of factor calculated by the random forest method, in the course of model construction. *x*-axis shows the gene names; *y*-axis shows the score of MeanDecreaseAccuracy and MeanDecreaseGini. Top 30 genes generated from random forest method were presented in the figures, measured by MeanDecreaseAccuracy and MeanDecreaseGini. (b) Regression course of the LASSO. (c) The figure showed the recommended area of log*λ* and number of factors that should be obtained (between two vertical dashed lines). The best tuning parameter (*λ*) was suggested as 0.0127231 by precise calculation. (d) A Venn diagram shows the intersection of variables from the random forest and LASSO's selection.

Compared with the external validation databases, 104 genes were used for variable selection, one of which was unavailable in external datasets. We used mean decrease accuracy and mean decrease Gini scores to select the most significant variables in random forest model, with the top 30 genes of both mean decrease accuracy and mean decrease Gini shown in Figure 5(a). Union of the two groups contained 47 genes, which were used for model construction (Figure 5(a), Table S4). For the lasso modeling, best tuning parameter (*λ*) was suggested as 0.0127231. We set *λ* =0.01 for variable selection, with 39 variables obtained from LASSO regression model (Figures 5(b) and 5(c), Table S5). The top 22 most significant classifier variables from both machine learning algorithms were kept and used to generate logistic regression models (Figure 5(d)). In total, six genes were kept in the regression model (*SMU1*, *CLIC3*, *SP100*, *ARHGAP25*, *DECR1*, and *TNS3*).

### 3.5. Classification and Mortality Validation in Derivation Dataset

**Figure 6:**
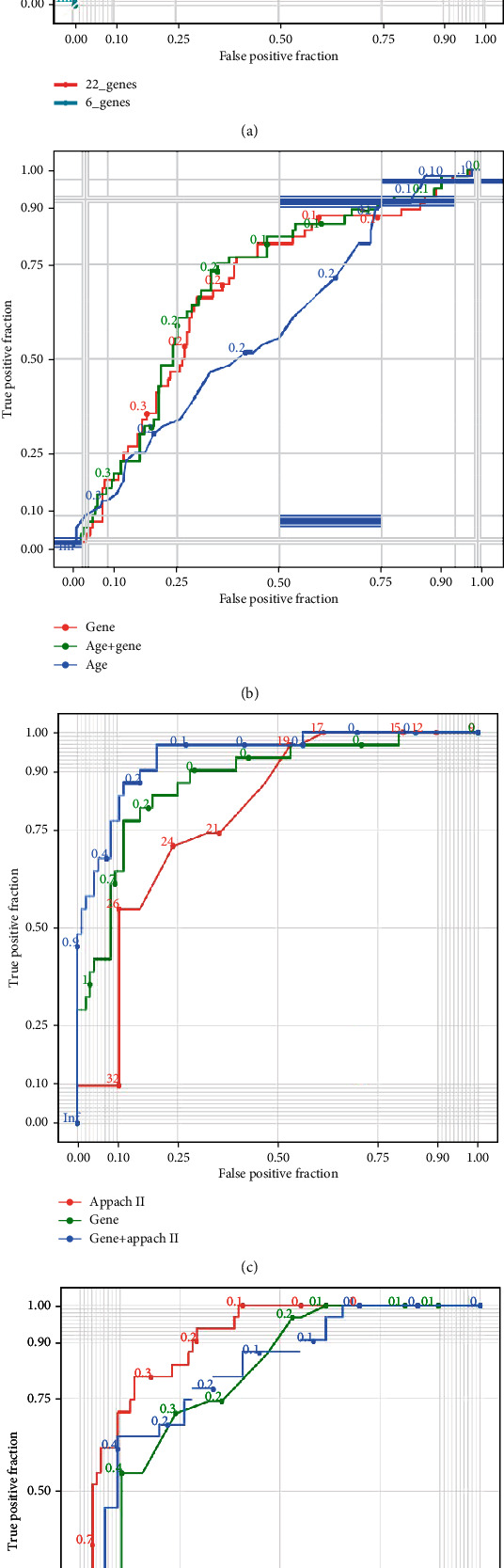
Validation of six-gene model of classification and mortality prediction (a) The AUC of the six-gene class model in the group prediction was 0.958 (95% CI: 0.892–0.872), and the AUC of the 22-gene class model in the group prediction was 0.955 (95% CI: 0.892–0.897). (b) Validation of the mortality prediction of the six-gene model and age in GSE65682. The AUC for the six-gene model was 0.681 (95% CI: 0.603–0.768), the AUC for age was 0.585 (95% CI: 0.247–0.911), and the AUC for the six-gene-age model was 0.695 (95% CI: 649–0.750). (c) Validation of the mortality prediction of the six-gene model and age in GSE95233. The AUC of the six-gene model was 0.818 (95% CI: 0.906–0.645), the AUC of age was 0.676 (95% CI: 0.427–0.968), and the AUC of the six-gene-age model was 0.838 (95% CI: 0.594–0.935). (d) Validation of the mortality prediction of the six-gene model and appach II score in GSE54514. The AUC of the six-gene model was 0.818 (95% CI: 0.906–0.645), the AUC of appach II was 0.792 (95% CI: 0.760–0.710), and the AUC of the six-gene-appach II model was 0.906 (95% CI: 0.865–0.839).

As the derivation dataset, GSE65682 was grouped into the training set and testing set at 4 : 1. Classification efficiency of the 22-gene model was well validated in the test dataset generated from the validation data, with an AUC of 0.955 (95% CI: 0.892–0.897) (Figure 6(a)). Classification efficiency of the six-gene model presented as strongly as the 22-gene model, while an AUC of 0.958 (95% CI: 0.892–0.872), and *p*-value in the chi-square test of the two models was 0.1479.

We then evaluated the mortality prediction value with the six-gene model. The six-gene model from classification training was satisfactory in predicting mortality (AUC 0.681 [95% CI: 0.603–0.768]). We also trained the six-gene model for mortality (AUC 0.699 [95% CI: 0.684–0.679]), with the prediction efficiency of the two models demonstrating no statistical difference (*p* =3.6724). Thus, we kept the model from classification training for further analysis, in order to balance the accuracy of classification and death prediction.

In the generated dataset, we assessed the predictive power of age on mortality, where the AUC for age was 0.585 (95% CI: 0.247–0.911) and the AUC for the six-gene-age model was 0.695 (95% CI: 649–0.750) (Figure 6(b)). The six-gene model performed better than the six-gene-age model (*p* =0.0004); when age was integrated, the six-gene-age model performed slightly better (*p* =0.0001) than the six-gene model.

The same method was applied in the GSE54514 external validation set. The six-gene model generated from GSE65682 did not fit well in GSE54514 (AUC 0.478 [95% CI: 0.969, 0.129]). Considering the different data background, we retrained the six genes for mortality in GSE54514, with a much stronger result. We compared the six-gene model to age and appach II scoring system in mortality prediction efficiency in GSE54514. The AUC of the six-gene model was 0.818 (95% CI: 0.906–0.645), the AUC of age was 0.676 (95% CI: 0.427–0.968), and the AUC of the six-gene-age model was 0.838 (95% CI: 0.594–0.935) (Figure 6(c)). We observed that in the external validation set, the model predicted mortality more accurately than age (*p* =0.0004), and that its predictive power was greatly enhanced after integrating age (*p* =0.0208).

When compared to the appach II scoring system in GSE54514, the AUC of appach II was 0.792 (95% CI: 0.760–0.710), and the AUC of the six-gene-appach II model was 0.906 (95% CI: 0.865–0.839) (Figure 6(d)). Similarly, the ability of our model to predict mortality is slightly higher than that of the appach II scoring system (*p* =0.0241), with the six-gene model improved when the appach II score was integrated when predicting mortality (*p* <0.001). Nomograph and calibration curve are presented in Figure S3A,3B.

In GSE57065, we compared our classification with simplified acute physiology score II (SAP II). In patients with mild sepsis, the ratio of Class 2 members was 14.3% (6/42) and 40.0% (13/42) in the severe sepsis group, with a statistically significant difference in ratio of Class 2 members (*p* <0.01).

In GSE131761, we observed the performance of the classification model in shock patients with or without sepsis and found a significantly higher proportion of Class 2 members in the population without shock (17.6%, 6/34) than in the population of patients with shock (22.2%, 18/81), with a statistical difference (*p* <0.01). This may reflect the insufficiency of our model in recognition of infection and inflammatory course.

## 4. Discussion

Sepsis cardiomyopathy is a common complication of sepsis and has a negative impact on the mortality of patients. The conventional diagnostic methods include echocardiograph and some laboratory tests, such as BNP, cTnI, and myocardial enzymes [3]. BNP and cTnI are helpful for the prognosis and diagnosis of sepsis cardiomyopathy, but the further research revealed they are more closely tied to the severity of sepsis than the specific cardiac function [9, 12]. Because of the lack of specificity of the above biochemical tests, more efficient and specific biological markers are still in high demand in clinical work. Although some gene expression profiling studies on sepsis have been carried out, studies on myocardial and peripheral blood specimens have been conducted independently, and few omics studies describing their association exist [16–20].

Our study analyzed co-expressed genes in myocardium and peripheral blood samples from patients with sepsis and used these genes for clustering analysis. Our aim was to identify the population with myocardial injury by analyzing the peripheral blood genes of sepsis patients. As mentioned in the introduction, the mechanism of SCM is complicated and inflammatory reactions play a great role in this mechanism. LPS, IL-1, TNF, prostanoids, and nitric oxide could work as depressant factors for myocardium in severe septic patients [8, 32–34]. Exosome and mitochondrial function are also involved in the process. Exosome containing nicotinamide adenine dinucleotide phosphate (NADPH) could cause reactive oxygen species production, vascular apoptosis, and myocardium dysfunction by mechanism that is associated with inflammatory. Inhibition of platelet exosome would be beneficial for sepsis patients [4]. At the meantime, mitochondrial dysfunction has already been proven to be a systemic problem in sepsis, especially for the myocardium [5–7]. It has been reported that in the myocardial tissue of sepsis patients, energy metabolism proteins such as mitochondria-related protein and myocardial contractility-related proteins are significantly downregulated [16]. Mitochondrial dysfunction has been shown in other organs and tissues taken from septic patients including skeletal muscle [35–37], platelets [38–40], and peripheral blood mononuclear cells (PBMCs) [41, 42]. Plasma mitochondrial DNA levels have also been associated with the incidence of acute respiratory distress syndrome (ARDS) in trauma and sepsis patients [43].

Our study verified the conclusions outlined above from the perspective of bioinformatics. By annotating DEGs with GO database and GSEA, we found some pathways of inflammatory factors, autophagy, macroautophagy, and proteolysis were upregulated. Conversely, pathways about mitochondrial, aerobic respiratory, and NADPH were downregulated. All of the pathological processes of myocardial depressant factors, exosomes, and mitochondria were systemic, in another word, they happened in both in the myocardium and peripheral blood.

Using the co-expressed DEGs, patients with sepsis were divided into two groups by *k*-means clustering analysis. Class2 accounted for approximately 25% of the total database and had a mortality rate twice that of Class1. Previous attempts to classify sepsis subsets have been reported [44–46], but most of them were based on patterns of the strength of the inflammatory response. In our classification model, mitochondrial and aerobic metabolism-related functional abnormalities were added on the basis of inflammatory response, so as to better accord with the characteristics of sepsis cardiomyopathy. Although mitochondrial and aerobic metabolic processes were considered, in the classification and modeling process, infection-related inflammatory response remained a major factor affecting sepsis severity and mortality. Our six-gene model contained *SMU1*, *CLIC3*, *SP100*, *ARHGAP25*, *DECR1*, and *TNS3* (Table S2, Figure 2(c)). Few of them have been previously related to septic cardiomyopathy, but some indirect information has been identified by previous studies. The chloride intracellular channel 3 (CLIC3) is a voltage-sensitive channel found across the cellular membrane, and no research has directly validated its function to septic cardiomyopathy. Interestingly, the disturbance of serum chloride level (hypochloremia) and different kinds of chloride channels have been observed to have adverse effect on acute and chronic heart failure, by causing dysregulation of renin secretion and fluid retention [47]. The 2,4-dienoyl-CoA reductase 1(*DECR1*) gene has been reported as one of the most significantly upregulated hub genes in diabetic cardiomyopathy, but further validation has not been performed [48]. *SP100* is a type of nuclear antigen which encodes a subnuclear organelle and is major component of the PML- (promyelocytic leukemia-) SP100 nuclear bodies. Despite epidemiological research linking sepsis and cancer, including acute and chronic leukemia [49], more studies are needed to verify the function of SP100 in sepsis and septic cardiomyopathy.

According to the mortality prediction analysis of our model, the six-gene model performed better than age and appach II score did, and its prediction ability can be further improved after integrating age and appach II score.

Our study found that upregulation of inflammation-related pathways and downregulation of mitochondrial and aerobic respiration-related functions co-exist in the blood and myocardium of sepsis patients. Study of key targets of these pathways can help us further understand the pathogenesis of septic cardiomyopathy. We identified 1,049 co-expressed genes, 104 of which were associated with mortality, and ultimately six genes were used to construct models for identifying sepsis subgroups and predicting mortality with satisfied validation. This work has allowed us to build a genetic dataset that can help us to screen and validate biological markers more purposefully in the future.

Our study has several limitations. First, many publicly available sepsis datasets primarily focus on the differential diagnosis of sepsis versus noninfectious systematic inflammatory response syndrome and did not report mortality outcome. Additionally, it was difficult to find a database that contained enough results from cardiac ultrasound and other laboratory tests, which made it challenging to compare our model with these routine tests in terms of diagnostic efficiency and mortality prediction.

Shock induced by septic cardiomyopathy has poor response to fluid resuscitation. GSE110487 is a gene sequencing study based on fluid response grouping. We tried to use hub genes in our model to predict patients' responses to fluid resuscitation, but the result was negative [15]. In fact, in the original study of GSE110487, patients who responded to fluid therapy and those who did not could not be well separated by principal component analysis of peripheral blood omics data. Therefore, the results predicted by our model are not indicative of the diagnostic efficiency of sepsis cardiac dysfunction.

Second, our study was designed to detect cardiac dysfunction in patients with sepsis. We validated our classification model using data from GSE131761 and found a higher proportion of group two patients in nonseptic shock [50]. This may suggest that our model of classification focuses more on cardiac dysfunction and less on infection. Therefore, it remains to be seen whether the prediction results have advantages over traditional inspection methods.

As mentioned above, there is still a great deal of work to be done on the omics of septic cardiomyopathy. We need a better prospective study design that includes a clear definition of septic cardiomyopathy and more laboratory and clinical data on myocardial injury. Similarly, although very difficult, we would expect to have blood and cardiac tissue samples from the same patient to complete the design of a paired trial.

## 5. Conclusion

We identified 1,049 differently expressed genes regulated the same way in both myocardium and blood, including 549 genes that were upregulated and 500 genes that were downregulated, 105 genes of which were found to be related to mortality in sepsis patients. By GO annotation and GSEA, we determined that upregulated genes were related to inflammatory pathways, and downregulated genes were regulated to mitochondrial and aerobic metabolism. These findings revealed that the downregulation of mitochondrial and aerobic metabolism was synchronously changed in both peripheral blood and myocardium in septic patients.

After applying clustering analysis by using the 104 genes mentioned above, sepsis patients were divided into two groups with significantly different mortality. As such, we proved that those genes in the blood that change in parallel with the myocardium can work as characteristic genes for the subtypes identification of sepsis and influence the morbidity and mortality between the different subtypes. A six-gene model was obtained by machine learning method (LASSO and random forest) and logical regression. This model can be well validated in derivation dataset and external dataset in both classification and mortality prediction. Therefore, we believe that the changes in peripheral blood of the genes screened in our work could potentially reflect some of the same changes in myocardial tissue, and have the potential to be valid biomarkers.

## Data Availability

All the datasets used in our study are obtained in https://www.ncbi.nlm.nih.gov/geo/.

## Supplementary Materials

Supplementary Materials Supplement Figure S1. Correlations between different module memberships generated by weighted correlation network analysis (WGCNA). The gene significances were calculated and are presented in a scatter plot. Correlation coefficients and *p*-values are shown on the top of the figure. The blue, brown, grey, and turquoise modules were found to be linearly correlated with gene significance. Figure S2. The *x*-axis of the bar plot indicates the possible clustering method suggested by *k*-means clustering analysis, and the *y*-axis presents the evaluation scores of the different clustering methods. The *k*-means clustering analysis suggests that the best classification is dichotomous. B. The eigenvalue decreases rapidly as the component number increases at the beginning, especially when the first two components are added. The decrease in the eigenvalue was less obvious when more than three components were added. A scree plot shows that two components can well describe the characteristics of the groups clustered by the clustering analysis. Figure S3. Nomograph displaying the risk score of each risk factor when six genes were integrated with the age and appach II score, including *SMU1*, *SP100*, and *ARHGAP25*, which contribute great weight to mortality. Calibration curve in GSE54514, which shows the good fit of our model. The mean absolute error is acceptable (mean absolute error =0.033). Table S1. A total of 1,049 DEGs changed in the same way in both the myocardium and blood datasets, including 549 genes upregulated and 500 genes downregulated. There were 325 genes in the blue module, 116 genes in the brown module, 305 genes in the turquoise module, and 261 genes in the gray module. Table S2. Detailed results of Gene Ontology (GO) analysis and GeneSet Enrichment Analysis (GSEA) of the turquoise and blue modules, as supplementary material for Figure 2. This table displays all the GO analysis pathways, including biological process (BP), cell component (CC), and molecular function (MF), as well as the GSEA pathway details. Table S3. Detailed data of a total of 105 genes out of the 1,049 differentially expressed genes (DEGs) shown in Table S1 verified to be related to mortality in the group of sepsis patients. Table S4. Mean decrease accuracy and mean decrease Gini scores of all 105 genes received random forest method, as complementary material for Figure S3. Table S5. Regression coefficients of 39 genes selected by LASSO.
