# Supplementary material for: Exploration of the Shared Gene Signatures between Myocardium and Blood in Sepsis: Evidence from Bioinformatics Analysis
Source: Biomed Res Int. 2022 Aug 6;2022:3690893. doi: 10.1155/2022/3690893 (PMC9375705; doi:10.1155/2022/3690893)
Supplement: Supplementary Materials — Supplement Figure S1. Correlations between different module memberships generated by weighted correlation network analysis (WGCNA). The gene significances were calculated and are presented in a scatter plot. Correlation coefficients and p-values are shown on the top of the figure. The blue, brown, grey, and turquoise modules were found to be linearly correlated with gene significance. Figure S2. The x-axis of the bar plot indicates the possible clustering method suggested by k-means clustering analysis, and the y-axis presents the evaluation scores of the different clustering methods. The k-means clustering analysis suggests that the best classification is dichotomous. B. The eigenvalue decreases rapidly as the component number increases at the beginning, especially when the first two components are added. The decrease in the eigenvalue was less obvious when more than three components were added. A scree plot shows that two components can well describe the characteristics of the groups clustered by the clustering analysis. Figure S3. Nomograph displaying the risk score of each risk factor when six genes were integrated with the age and appach II score, including SMU1, SP100, and ARHGAP25, which contribute great weight to mortality. Calibration curve in GSE54514, which shows the good fit of our model. The mean absolute error is acceptable (mean absolute error =0.033). Table S1. A total of 1,049 DEGs changed in the same way in both the myocardium and blood datasets, including 549 genes upregulated and 500 genes downregulated. There were 325 genes in the blue module, 116 genes in the brown module, 305 genes in the turquoise module, and 261 genes in the gray module. Table S2. Detailed results of Gene Ontology (GO) analysis and GeneSet Enrichment Analysis (GSEA) of the turquoise and blue modules, as supplementary material for Figure 2. This table displays all the GO analysis pathways, including biological process (BP), cell component (CC), and molecular function (MF), as [file 3690893.f1.zip › Figure-Supplement.docx]

Figure S1

A B


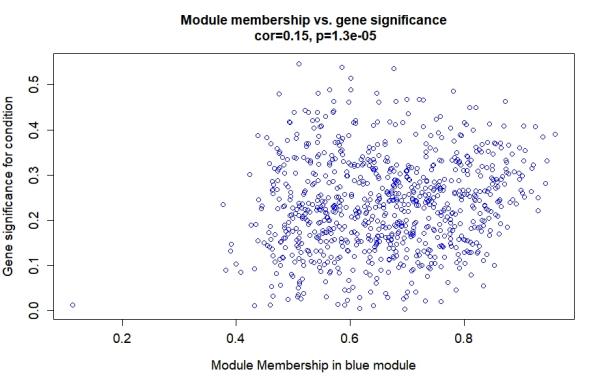

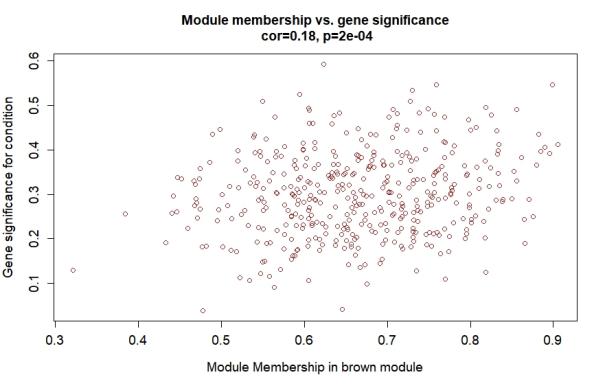


C D


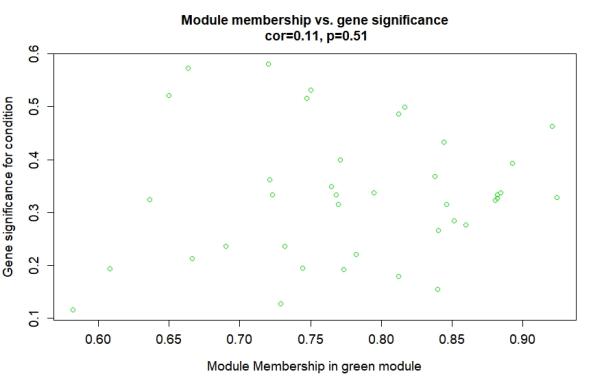

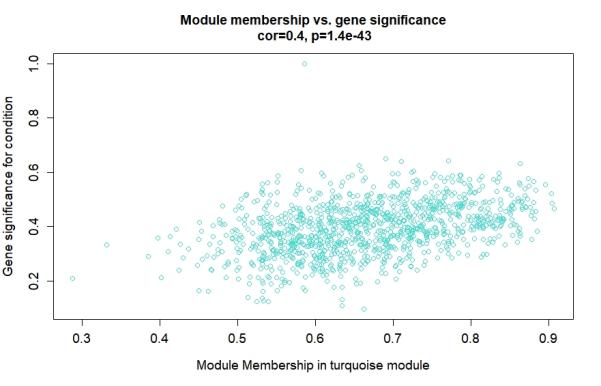


E F


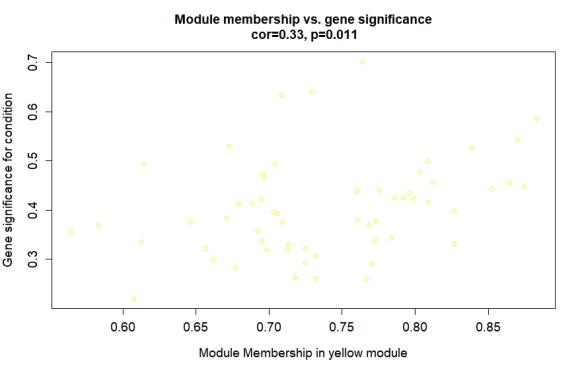

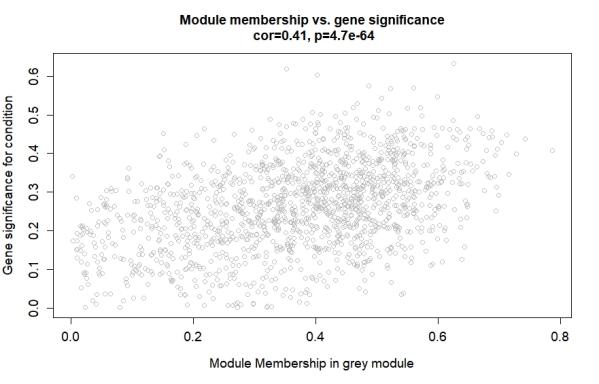


Correlations between different module memberships generated by weighted correlation network analysis (WGCNA). The gene significances were calculated and are presented in a scatter plot. Correlation coefficients and p-values are shown on the top of the figure. The blue, brown, grey, and turquoise modules were found to be linearly correlated with gene significance.

Figure S2

A B


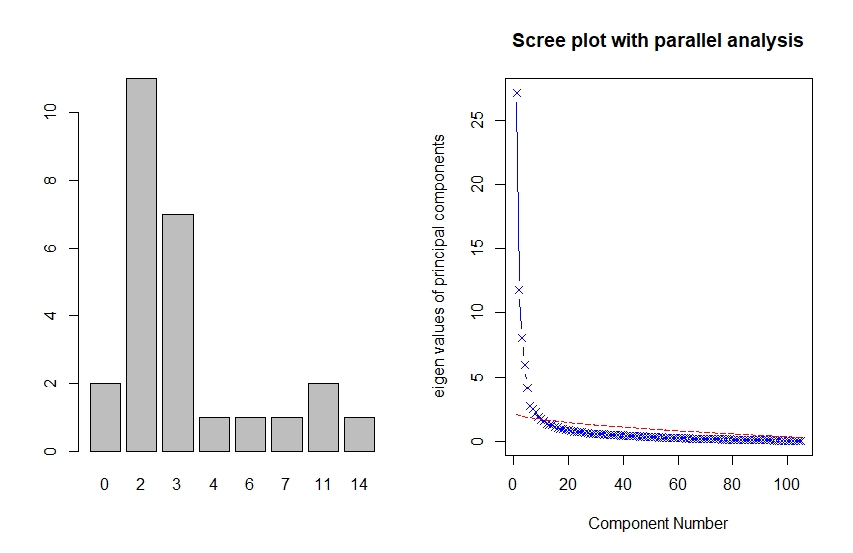


1. The X-axis of the bar plot indicates the possible clustering method suggested by k-means clustering analysis, and the y-axis presents the evaluation scores of the different clustering methods. The k-means clustering analysis suggests that the best classification is dichotomous.

B. The eigenvalue decreases rapidly as the component number increases at the beginning, especially when the first two components are added. The decrease in the eigenvalue was less obvious when more than three components were added. A scree plot shows that two components can well describe the characteristics of the groups clustered by the clustering analysis.

Figure S3

A


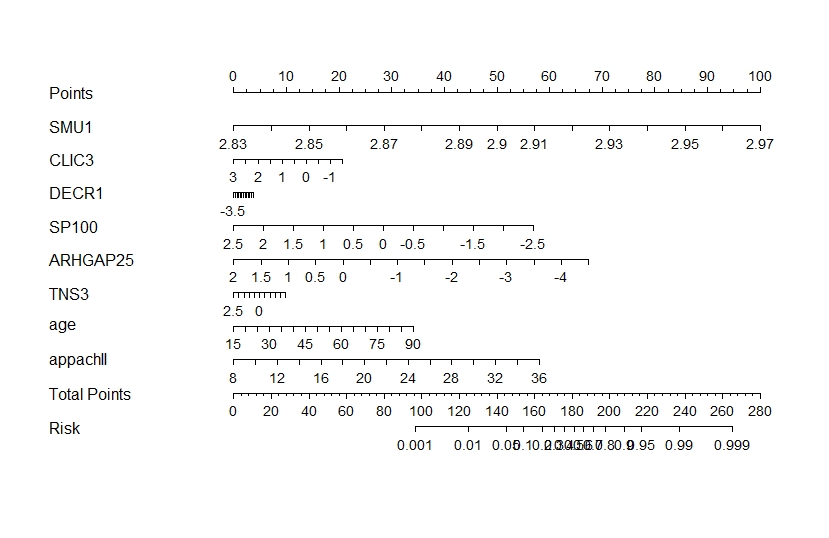


B


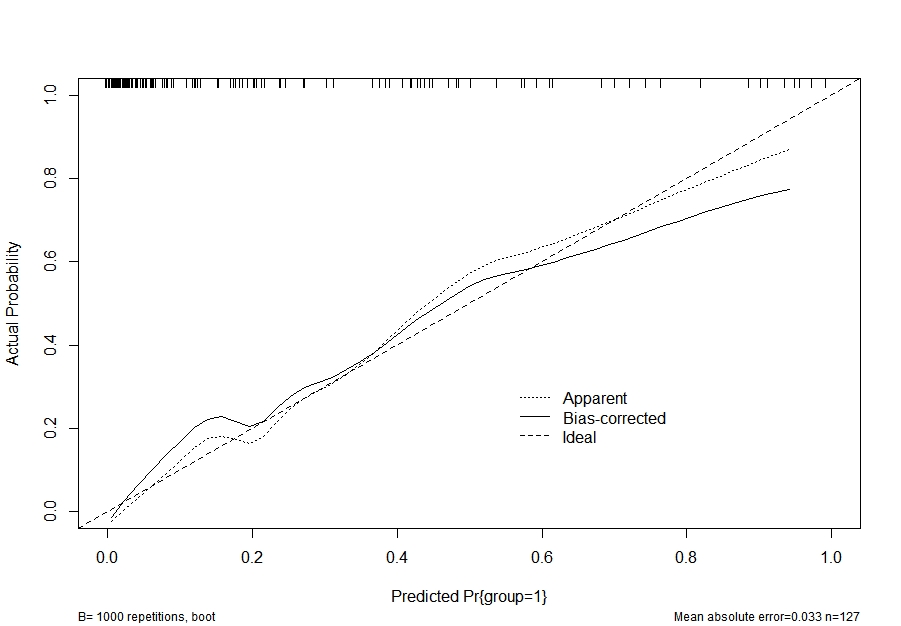


1. Nomograph displaying the risk score of each risk factor when six genes were integrated with the age and appach II score, including SMU1, SP100, and ARHGAP25, which contribute great weight to mortality.
2. Calibration curve in GSE54514, which shows the good fit of our model. The mean absolute error is acceptable (mean absolute error = 0.033).
