# Supplementary material for: Exploration of the Shared Gene Signatures between Myocardium and Blood in Sepsis: Evidence from Bioinformatics Analysis
Source: Biomed Res Int. 2022 Aug 6;2022:3690893. doi: 10.1155/2022/3690893 (PMC9375705; doi:10.1155/2022/3690893)
Supplement: Supplementary Materials — Supplement Figure S1. Correlations between different module memberships generated by weighted correlation network analysis (WGCNA). The gene significances were calculated and are presented in a scatter plot. Correlation coefficients and p-values are shown on the top of the figure. The blue, brown, grey, and turquoise modules were found to be linearly correlated with gene significance. Figure S2. The x-axis of the bar plot indicates the possible clustering method suggested by k-means clustering analysis, and the y-axis presents the evaluation scores of the different clustering methods. The k-means clustering analysis suggests that the best classification is dichotomous. B. The eigenvalue decreases rapidly as the component number increases at the beginning, especially when the first two components are added. The decrease in the eigenvalue was less obvious when more than three components were added. A scree plot shows that two components can well describe the characteristics of the groups clustered by the clustering analysis. Figure S3. Nomograph displaying the risk score of each risk factor when six genes were integrated with the age and appach II score, including SMU1, SP100, and ARHGAP25, which contribute great weight to mortality. Calibration curve in GSE54514, which shows the good fit of our model. The mean absolute error is acceptable (mean absolute error =0.033). Table S1. A total of 1,049 DEGs changed in the same way in both the myocardium and blood datasets, including 549 genes upregulated and 500 genes downregulated. There were 325 genes in the blue module, 116 genes in the brown module, 305 genes in the turquoise module, and 261 genes in the gray module. Table S2. Detailed results of Gene Ontology (GO) analysis and GeneSet Enrichment Analysis (GSEA) of the turquoise and blue modules, as supplementary material for Figure 2. This table displays all the GO analysis pathways, including biological process (BP), cell component (CC), and molecular function (MF), as [file 3690893.f1.zip › Table-S1.pdf]

|  | logFC | AveExpr | t | P.Value | adj.P.Val | B | module |
|---|---|---|---|---|---|---|---|
| P2RY14 | 0.465312 | 2.155245 | 7.116615 | 1.19E-09 | 6.94E-06 | 11.70159 | 3 |
| GPAM | 0.320981 | 2.211 | 6.687208 | 6.71E-09 | 1.92E-05 | 10.09799 | 3 |
| LRRN3 | 0.367575 | 1.775003 | 6.516986 | 1.33E-08 | 2.32E-05 | 9.46529 | 0 |
| CYB5D1 | 0.201895 | 2.568819 | 5.760343 | 2.63E-07 | 0.000192 | 6.689844 | 1 |
| PDP1 | 0.191414 | 3.001284 | 5.644851 | 4.12E-07 | 0.00023 | 6.273535 | 3 |
| LYRM7 | 0.226245 | 2.745162 | 5.412575 | 1.01E-06 | 0.000383 | 5.444018 | 2 |
| SYNE1 | 0.186481 | 2.578762 | 5.127905 | 2.96E-06 | 0.000716 | 4.443648 | 3 |
| MRPS33 | 0.232189 | 2.882504 | 5.125225 | 2.99E-06 | 0.000716 | 4.434324 | 2 |
| GPR34 | 0.252655 | 2.086309 | 5.059147 | 3.83E-06 | 0.000837 | 4.205048 | 0 |
| IFIT1 | 0.33839 | 1.939218 | 5.040431 | 4.10E-06 | 0.000865 | 4.140319 | 1 |
| NDUFB5 | 0.280441 | 3.145703 | 4.983703 | 5.07E-06 | 0.000979 | 3.944714 | 2 |
| PHB | 0.239533 | 3.360372 | 4.976589 | 5.21E-06 | 0.000979 | 3.920249 | 2 |
| MRPL16 | 0.290448 | 3.056052 | 4.950993 | 5.72E-06 | 0.001037 | 3.832337 | 2 |
| MRPS25 | 0.280488 | 2.874672 | 4.946767 | 5.81E-06 | 0.001037 | 3.817843 | 2 |
| FAM78A | 0.228488 | 2.165255 | 4.922498 | 6.36E-06 | 0.001099 | 3.734691 | 1 |
| MTSS1 | 0.230275 | 2.798635 | 4.910037 | 6.66E-06 | 0.001099 | 3.692064 | 1 |
| PTPN4 | 0.157413 | 2.244209 | 4.882708 | 7.37E-06 | 0.001156 | 3.598736 | 3 |
| IMP3 | 0.112589 | 2.605481 | 4.88206 | 7.39E-06 | 0.001156 | 3.596526 | 2 |
| DLAT | 0.274418 | 3.085342 | 4.873595 | 7.62E-06 | 0.001156 | 3.567664 | 2 |
| MRPL48 | 0.269856 | 2.488276 | 4.807078 | 9.73E-06 | 0.001297 | 3.341623 | 2 |
| RASSF4 | 0.186263 | 2.565271 | 4.787899 | 1.04E-05 | 0.001335 | 3.276703 | 3 |
| PDHB | 0.270267 | 3.243881 | 4.764566 | 1.14E-05 | 0.0014 | 3.197874 | 2 |
| PDE7A | 0.347935 | 2.967434 | 4.694519 | 1.47E-05 | 0.001635 | 2.962264 | 3 |
| COQ10A | 0.384322 | 3.127921 | 4.694302 | 1.47E-05 | 0.001635 | 2.961537 | 3 |
| GTPBP8 | 0.159339 | 2.477454 | 4.694279 | 1.47E-05 | 0.001635 | 2.96146 | 2 |
| DSTN | 0.210055 | 3.405282 | 4.648428 | 1.73E-05 | 0.001783 | 2.808106 | 2 |
| KLF13 | 0.198657 | 2.50403 | 4.644586 | 1.76E-05 | 0.001783 | 2.795288 | 1 |
| ISOC1 | 0.22813 | 3.107402 | 4.641122 | 1.78E-05 | 0.001783 | 2.783733 | 2 |
| MTIF2 | 0.264356 | 2.766737 | 4.636652 | 1.81E-05 | 0.001783 | 2.768832 | 2 |
| TC2N | 0.250389 | 1.998214 | 4.621532 | 1.91E-05 | 0.001835 | 2.718476 | 0 |
| SLC25A26 | 0.166904 | 2.610187 | 4.590062 | 2.14E-05 | 0.001978 | 2.613911 | 2 |
| TMEM203 | 0.19184 | 3.138409 | 4.549816 | 2.47E-05 | 0.002172 | 2.48069 | 2 |
| NCALD | 0.231314 | 2.02826 | 4.520979 | 2.74E-05 | 0.002287 | 2.385581 | 1 |
| TTYH2 | 0.199862 | 2.55418 | 4.492219 | 3.04E-05 | 0.002361 | 2.291022 | 1 |
| MRPL39 | 0.333085 | 2.937002 | 4.491147 | 3.05E-05 | 0.002361 | 2.287504 | 2 |
| TIMM21 | 0.367508 | 3.051133 | 4.489553 | 3.07E-05 | 0.002361 | 2.282271 | 2 |
| ATXN7L3B | 0.160494 | 3.074674 | 4.456396 | 3.45E-05 | 0.002588 | 2.173658 | 3 |
| FASTKD1 | 0.23427 | 2.563353 | 4.449526 | 3.53E-05 | 0.002641 | 2.151202 | 2 |
| AKAP1 | 0.239042 | 3.044477 | 4.353525 | 4.96E-05 | 0.0032 | 1.839271 | 3 |
| APOOL | 0.235897 | 3.004635 | 4.324553 | 5.49E-05 | 0.003466 | 1.745821 | 2 |
| MRPL34 | 0.173044 | 2.577151 | 4.30306 | 5.92E-05 | 0.003644 | 1.676706 | 2 |
| NDUFAB1 | 0.33386 | 3.68889 | 4.265349 | 6.75E-05 | 0.003936 | 1.555876 | 2 |
| SNRPC | 0.219468 | 3.013931 | 4.263896 | 6.78E-05 | 0.003936 | 1.551233 | 2 |
| TOB1 | 0.177013 | 2.890216 | 4.24687 | 7.20E-05 | 0.004015 | 1.496877 | 3 |
| VDAC3 | 0.239679 | 3.402581 | 4.243591 | 7.28E-05 | 0.004016 | 1.48642 | 2 |
| MLH1 | 0.163369 | 2.839939 | 4.235089 | 7.50E-05 | 0.004085 | 1.459331 | 2 |
| EBF1 | 0.166585 | 2.607508 | 4.22471 | 7.77E-05 | 0.004167 | 1.426303 | 1 |
| MRPS30 | 0.231983 | 2.969173 | 4.224012 | 7.79E-05 | 0.004167 | 1.424082 | 2 |
| DECR1 | 0.211369 | 3.268662 | 4.216269 | 8.00E-05 | 0.004232 | 1.399472 | 2 |
| BCL11A | 0.206174 | 2.115395 | 4.214242 | 8.06E-05 | 0.004232 | 1.393033 | 1 |

| | | | | | | | |
|---|---|---|---|---|---|---|---|
| HEATR5B | 0.170843 | 2.408543 | 4.195732 | 8.59E-05 | 0.004408 | 1.334318 | 2 |
| MPEG1 | 0.292758 | 2.088403 | 4.192394 | 8.69E-05 | 0.004408 | 1.323743 | 0 |
| KAT6B | 0.207317 | 2.691347 | 4.186536 | 8.87E-05 | 0.004435 | 1.305198 | 3 |
| PMVK | 0.19077 | 2.565823 | 4.171413 | 9.34E-05 | 0.004577 | 1.257389 | 2 |
| EI24 | 0.227777 | 2.647751 | 4.146381 | 0.000102 | 0.00488 | 1.17846 | 2 |
| EPM2AIP1 | 0.16647 | 2.508963 | 4.137815 | 0.000105 | 0.004956 | 1.151511 | 3 |
| OSGEPL1 | 0.202475 | 2.078649 | 4.130434 | 0.000108 | 0.005029 | 1.128314 | 3 |
| NDUFA9 | 0.293734 | 3.428911 | 4.126297 | 0.000109 | 0.005087 | 1.115324 | 2 |
| EBP | 0.154569 | 2.906356 | 4.123323 | 0.00011 | 0.005092 | 1.105988 | 2 |
| ZNF770 | 0.228624 | 2.751846 | 4.112171 | 0.000114 | 0.0052 | 1.071021 | 2 |
| HIGD2A | 0.281778 | 3.527276 | 4.10926 | 0.000116 | 0.005225 | 1.0619 | 2 |
| TACO1 | 0.216515 | 2.712962 | 4.092054 | 0.000123 | 0.005443 | 1.008071 | 2 |
| SMC2 | 0.202169 | 2.313886 | 4.082133 | 0.000127 | 0.005573 | 0.977093 | 3 |
| RNF125 | 0.226429 | 1.813451 | 4.069283 | 0.000133 | 0.005682 | 0.937029 | 0 |
| CYC1 | 0.283466 | 3.668393 | 4.057463 | 0.000138 | 0.005783 | 0.90024 | 2 |
| TRMT61B | 0.214773 | 2.055678 | 4.050819 | 0.000141 | 0.005861 | 0.879587 | 0 |
| TNRC6A | 0.159596 | 2.836524 | 4.045193 | 0.000144 | 0.005892 | 0.862115 | 3 |
| SH3KBP1 | 0.135493 | 2.605195 | 4.03922 | 0.000147 | 0.005964 | 0.84358 | 0 |
| VIPR1 | 0.158444 | 2.005504 | 4.02869 | 0.000152 | 0.006046 | 0.810943 | 1 |
| CHN2 | 0.201203 | 2.503648 | 4.02146 | 0.000156 | 0.006121 | 0.788562 | 1 |
| NDUFAF4 | 0.269546 | 2.884577 | 4.020307 | 0.000156 | 0.006121 | 0.784993 | 2 |
| NDUFS3 | 0.237033 | 3.252136 | 4.005942 | 0.000164 | 0.006285 | 0.740602 | 2 |
| CBX5 | 0.179971 | 3.154605 | 3.981289 | 0.000178 | 0.00664 | 0.664632 | 2 |
| MRPS10 | 0.292139 | 2.994945 | 3.97412 | 0.000183 | 0.006745 | 0.64259 | 2 |
| SDHD | 0.236879 | 3.409146 | 3.948353 | 0.000199 | 0.007202 | 0.56356 | 2 |
| SPOP | 0.118563 | 2.725979 | 3.942035 | 0.000204 | 0.007307 | 0.544229 | 2 |
| NREP | 0.231372 | 2.68785 | 3.941563 | 0.000204 | 0.007307 | 0.542784 | 1 |
| NDUFC1 | 0.190444 | 3.34696 | 3.937359 | 0.000207 | 0.007381 | 0.529934 | 2 |
| SCP2 | 0.162058 | 2.856812 | 3.920374 | 0.000219 | 0.007702 | 0.478089 | 2 |
| C6orf136 | 0.149995 | 2.640312 | 3.914976 | 0.000223 | 0.007804 | 0.46164 | 2 |
| ZBED5 | 0.110156 | 2.699315 | 3.912716 | 0.000225 | 0.007822 | 0.454758 | 2 |
| DNAJC19 | 0.21507 | 3.111337 | 3.867644 | 0.000261 | 0.008481 | 0.31799 | 2 |
| NDUFA8 | 0.302488 | 3.407703 | 3.864133 | 0.000264 | 0.008531 | 0.307378 | 2 |
| TMEM116 | 0.238443 | 2.709985 | 3.853603 | 0.000273 | 0.008689 | 0.275579 | 3 |
| PTDSS1 | 0.354402 | 2.892562 | 3.841576 | 0.000284 | 0.008912 | 0.239324 | 2 |
| BLCAP | 0.117707 | 2.749456 | 3.838112 | 0.000288 | 0.008982 | 0.228894 | 1 |
| ASF1A | 0.168913 | 2.604801 | 3.829158 | 0.000296 | 0.009156 | 0.201961 | 2 |
| AGK | 0.149862 | 2.640481 | 3.825937 | 0.000299 | 0.009218 | 0.192283 | 2 |
| JARID2 | 0.149204 | 2.350701 | 3.81866 | 0.000307 | 0.009318 | 0.170435 | 3 |
| AIMP2 | 0.194855 | 3.163651 | 3.805453 | 0.00032 | 0.009608 | 0.130844 | 2 |
| CUL5 | 0.20714 | 2.597183 | 3.803505 | 0.000322 | 0.009653 | 0.125013 | 2 |
| PIK3R1 | 0.16386 | 3.117144 | 3.800833 | 0.000325 | 0.009688 | 0.117017 | 3 |
| ZYG11B | 0.227143 | 2.831851 | 3.791589 | 0.000335 | 0.009919 | 0.089379 | 2 |
| GATA2 | 0.18699 | 2.21177 | 3.780281 | 0.000348 | 0.010198 | 0.055626 | 0 |
| ST3GAL5 | 0.169636 | 1.920444 | 3.779612 | 0.000349 | 0.010198 | 0.05363 | 0 |
| TPCN1 | 0.186783 | 2.941502 | 3.768018 | 0.000362 | 0.01045 | 0.019092 | 3 |
| CACYBP | 0.203323 | 3.015119 | 3.760668 | 0.000371 | 0.010599 | -0.00277 | 2 |
| NDRG3 | 0.151656 | 2.702937 | 3.758638 | 0.000373 | 0.010635 | -0.0088 | 3 |
| AIFM1 | 0.227769 | 3.05558 | 3.755853 | 0.000377 | 0.010667 | -0.01708 | 2 |
| CYCS | 0.193526 | 3.387488 | 3.755376 | 0.000377 | 0.010667 | -0.01849 | 2 |
| PTER | 0.165972 | 2.542807 | 3.752106 | 0.000381 | 0.01069 | -0.0282 | 1 |

| | | | | | | | |
|---|---|---|---|---|---|---|---|
| REV1 | 0.188343 | 2.42764 | 3.742366 | 0.000394 | 0.010964 | -0.05709 | 2 |
| UCHL5 | 0.124504 | 2.654812 | 3.740814 | 0.000396 | 0.010977 | -0.06169 | 2 |
| METAP1 | 0.126033 | 2.856212 | 3.694937 | 0.000459 | 0.011981 | -0.1971 | 2 |
| APOBEC3C | 0.151511 | 2.063877 | 3.657046 | 0.000519 | 0.012967 | -0.30814 | 2 |
| FOXN3 | 0.136297 | 3.121321 | 3.642259 | 0.000544 | 0.013333 | -0.35128 | 2 |
| SUN1 | 0.111778 | 2.955836 | 3.637601 | 0.000553 | 0.013476 | -0.36484 | 3 |
| NDUFS6 | 0.183674 | 3.490129 | 3.597288 | 0.000629 | 0.014597 | -0.48178 | 2 |
| ZC3HC1 | 0.101992 | 2.068467 | 3.59534 | 0.000633 | 0.014649 | -0.48741 | 0 |
| HDHD2 | 0.236768 | 2.754164 | 3.591999 | 0.000639 | 0.014767 | -0.49706 | 2 |
| IDH3B | 0.223056 | 3.109764 | 3.582697 | 0.000659 | 0.014974 | -0.5239 | 2 |
| MCCC1 | 0.125995 | 2.723049 | 3.57936 | 0.000666 | 0.015072 | -0.53352 | 2 |
| SUCLA2 | 0.188763 | 2.983733 | 3.566449 | 0.000693 | 0.015446 | -0.57067 | 2 |
| ZNF503 | 0.155529 | 2.130549 | 3.558927 | 0.00071 | 0.015661 | -0.59227 | 0 |
| ING3 | 0.159744 | 2.445499 | 3.558889 | 0.00071 | 0.015661 | -0.59238 | 2 |
| FARS2 | 0.176724 | 2.432242 | 3.55409 | 0.000721 | 0.015821 | -0.60615 | 2 |
| MRPS17 | 0.151181 | 2.610699 | 3.550958 | 0.000728 | 0.015925 | -0.61513 | 2 |
| MEGF8 | 0.151303 | 3.036902 | 3.541389 | 0.000751 | 0.016246 | -0.64253 | 1 |
| PFKM | 0.247308 | 3.308081 | 3.538767 | 0.000757 | 0.016341 | -0.65003 | 3 |
| TOMM22 | 0.16987 | 3.160374 | 3.530852 | 0.000776 | 0.016631 | -0.67265 | 2 |
| ACVR2A | 0.127762 | 2.34325 | 3.527816 | 0.000784 | 0.016722 | -0.68131 | 2 |
| EEPD1 | 0.12013 | 1.851333 | 3.524789 | 0.000791 | 0.016849 | -0.68995 | 1 |
| CS | 0.25845 | 3.505393 | 3.522677 | 0.000796 | 0.01688 | -0.69597 | 2 |
| ADCK2 | 0.148527 | 1.756418 | 3.518664 | 0.000807 | 0.016991 | -0.70741 | 0 |
| MRPS7 | 0.273547 | 3.244184 | 3.514088 | 0.000818 | 0.017155 | -0.72044 | 2 |
| PNPLA4 | 0.20003 | 2.715682 | 3.50991 | 0.000829 | 0.01732 | -0.73233 | 2 |
| LANCL2 | 0.107726 | 2.221391 | 3.493388 | 0.000873 | 0.017901 | -0.77925 | 2 |
| GRSF1 | 0.147033 | 2.966034 | 3.487014 | 0.000891 | 0.018156 | -0.79731 | 2 |
| TFAM | 0.16542 | 2.790425 | 3.484091 | 0.000899 | 0.018238 | -0.80558 | 2 |
| MRPL44 | 0.171718 | 2.626786 | 3.476357 | 0.000921 | 0.018417 | -0.82746 | 2 |
| NDUFS2 | 0.179983 | 3.413556 | 3.474695 | 0.000926 | 0.018482 | -0.83216 | 2 |
| POLR3K | 0.097942 | 2.753743 | 3.471978 | 0.000934 | 0.018597 | -0.83983 | 2 |
| CTNNBIP1 | 0.128822 | 2.286003 | 3.468895 | 0.000943 | 0.018692 | -0.84853 | 1 |
| ALKBH7 | 0.150279 | 2.898394 | 3.461272 | 0.000966 | 0.018971 | -0.87003 | 2 |
| ACAA2 | 0.342297 | 3.176506 | 3.447787 | 0.001007 | 0.019473 | -0.90798 | 2 |
| ALDOC | 0.20852 | 3.419619 | 3.440444 | 0.00103 | 0.019763 | -0.92861 | 2 |
| HSD17B4 | 0.17628 | 3.064191 | 3.440095 | 0.001031 | 0.019763 | -0.92959 | 2 |
| MRPL11 | 0.120119 | 2.399661 | 3.427756 | 0.001072 | 0.020282 | -0.96417 | 2 |
| FDX1 | 0.171526 | 2.686885 | 3.425261 | 0.00108 | 0.020308 | -0.97115 | 2 |
| MRPS18B | 0.204982 | 3.179457 | 3.419272 | 0.0011 | 0.020556 | -0.9879 | 2 |
| CBLL1 | 0.106624 | 2.670205 | 3.414975 | 0.001115 | 0.020652 | -0.99991 | 2 |
| PMPCB | 0.231213 | 2.965207 | 3.409361 | 0.001135 | 0.020906 | -1.01558 | 2 |
| ZNF431 | 0.137682 | 2.557056 | 3.403694 | 0.001155 | 0.021152 | -1.03138 | 2 |
| SDHA | 0.170464 | 3.488555 | 3.399194 | 0.001171 | 0.021394 | -1.04391 | 3 |
| KLF12 | 0.130424 | 2.623642 | 3.391483 | 0.001199 | 0.02175 | -1.06536 | 3 |
| PPARA | 0.161033 | 2.829619 | 3.388571 | 0.00121 | 0.021878 | -1.07345 | 3 |
| FAM220A | 0.133033 | 2.717115 | 3.384145 | 0.001227 | 0.022096 | -1.08574 | 0 |
| CUTA | 0.118488 | 2.968364 | 3.377342 | 0.001252 | 0.02241 | -1.10461 | 2 |
| COA1 | 0.145354 | 2.952828 | 3.37645 | 0.001256 | 0.02241 | -1.10708 | 2 |
| AKR7A2 | 0.10909 | 2.6139 | 3.374052 | 0.001265 | 0.022507 | -1.11373 | 2 |
| TMEM19 | 0.115255 | 2.137411 | 3.370759 | 0.001278 | 0.022667 | -1.12284 | 0 |
| CERS6 | 0.262851 | 3.249043 | 3.368667 | 0.001286 | 0.022699 | -1.12863 | 3 |

| | | | | | | | |
|---|---|---|---|---|---|---|---|
| RNASE6 | 0.224006 | 2.395129 | 3.368651 | 0.001286 | 0.022699 | -1.12868 | 0 |
| WWOX | 0.100274 | 1.797116 | 3.367579 | 0.001291 | 0.022726 | -1.13165 | 0 |
| EIF1AX | 0.275927 | 3.350795 | 3.360698 | 0.001318 | 0.022901 | -1.15067 | 2 |
| TSEN2 | 0.101692 | 2.051799 | 3.351089 | 0.001358 | 0.023415 | -1.17718 | 0 |
| TSC1 | 0.168534 | 2.699992 | 3.349044 | 0.001366 | 0.023492 | -1.18282 | 3 |
| PCYOX1 | 0.134698 | 2.902938 | 3.347289 | 0.001374 | 0.023547 | -1.18766 | 2 |
| RPP14 | 0.146015 | 2.867022 | 3.339865 | 0.001405 | 0.023901 | -1.20809 | 2 |
| CLCN4 | 0.11224 | 2.356357 | 3.323716 | 0.001476 | 0.02463 | -1.25244 | 2 |
| RPP25L | 0.144035 | 2.776856 | 3.317729 | 0.001503 | 0.024965 | -1.26884 | 2 |
| OXSM | 0.135908 | 1.950678 | 3.313587 | 0.001522 | 0.025162 | -1.28018 | 0 |
| SLC41A1 | 0.25198 | 3.477349 | 3.311758 | 0.001531 | 0.025231 | -1.28518 | 2 |
| ARHGAP10 | 0.221933 | 2.808467 | 3.302984 | 0.001572 | 0.025606 | -1.30915 | 3 |
| MTRF1 | 0.11911 | 2.061849 | 3.302902 | 0.001573 | 0.025606 | -1.30937 | 2 |
| ADH5 | 0.151232 | 3.063073 | 3.301965 | 0.001577 | 0.025615 | -1.31193 | 2 |
| MAF | 0.179226 | 2.253192 | 3.292288 | 0.001624 | 0.026009 | -1.33831 | 1 |
| TMEM184C | 0.200508 | 2.583558 | 3.289853 | 0.001636 | 0.026154 | -1.34495 | 2 |
| GLYR1 | 0.171592 | 2.849968 | 3.287082 | 0.00165 | 0.026291 | -1.35249 | 2 |
| CCDC146 | 0.174441 | 1.928004 | 3.278397 | 0.001694 | 0.026691 | -1.37609 | 1 |
| TMEM131 | 0.130705 | 2.820939 | 3.277857 | 0.001697 | 0.026691 | -1.37756 | 2 |
| MRS2 | 0.119156 | 2.74702 | 3.272772 | 0.001723 | 0.026929 | -1.39136 | 2 |
| TUBE1 | 0.169395 | 2.607105 | 3.269474 | 0.00174 | 0.027076 | -1.4003 | 3 |
| ESD | 0.229305 | 3.176799 | 3.266396 | 0.001757 | 0.027256 | -1.40864 | 2 |
| ENTPD6 | 0.123775 | 2.764029 | 3.262866 | 0.001775 | 0.027523 | -1.41819 | 0 |
| ZNF493 | 0.161071 | 2.532696 | 3.244668 | 0.001876 | 0.028572 | -1.46735 | 2 |
| POLR2B | 0.195835 | 2.977484 | 3.243506 | 0.001882 | 0.028593 | -1.47048 | 2 |
| FH | 0.279252 | 3.061952 | 3.214611 | 0.002053 | 0.030467 | -1.54811 | 2 |
| ZNF808 | 0.186702 | 2.686854 | 3.207858 | 0.002094 | 0.030957 | -1.56619 | 2 |
| TNRC6B | 0.148734 | 2.750945 | 3.202218 | 0.00213 | 0.031336 | -1.58126 | 3 |
| EPB41L2 | 0.101796 | 2.815589 | 3.202095 | 0.002131 | 0.031336 | -1.58159 | 3 |
| LEO1 | 0.156294 | 2.536361 | 3.200208 | 0.002143 | 0.03146 | -1.58663 | 3 |
| UPRT | 0.133185 | 2.30467 | 3.196548 | 0.002166 | 0.031672 | -1.59639 | 2 |
| DNAJC15 | 0.221884 | 3.213895 | 3.193315 | 0.002187 | 0.031925 | -1.60502 | 2 |
| ITM2A | 0.158083 | 2.816652 | 3.182538 | 0.002259 | 0.032558 | -1.63371 | 0 |
| TMEM14A | 0.167145 | 2.359529 | 3.171121 | 0.002336 | 0.033301 | -1.66403 | 0 |
| SMDT1 | 0.20832 | 2.974742 | 3.17109 | 0.002337 | 0.033301 | -1.66411 | 2 |
| GSTK1 | 0.133046 | 3.24654 | 3.166821 | 0.002366 | 0.03367 | -1.67543 | 2 |
| SLC20A2 | 0.176664 | 2.820909 | 3.166508 | 0.002369 | 0.033674 | -1.67626 | 2 |
| METTL18 | 0.148526 | 1.989223 | 3.156844 | 0.002437 | 0.034207 | -1.70184 | 0 |
| TEFM | 0.152677 | 2.152033 | 3.152625 | 0.002468 | 0.034443 | -1.71299 | 0 |
| PDIK1L | 0.12465 | 1.608739 | 3.146948 | 0.00251 | 0.034914 | -1.72797 | 0 |
| LPAR5 | 0.101963 | 2.206234 | 3.139973 | 0.002562 | 0.035332 | -1.74635 | 0 |
| DNAJA3 | 0.11966 | 2.751388 | 3.119926 | 0.002718 | 0.036629 | -1.79903 | 2 |
| CACNA2D3 | 0.179307 | 1.82117 | 3.119861 | 0.002718 | 0.036629 | -1.7992 | 0 |
| NUDT7 | 0.198156 | 2.358514 | 3.116524 | 0.002745 | 0.036815 | -1.80795 | 3 |
| BCKDHB | 0.117372 | 2.632252 | 3.108724 | 0.002809 | 0.037406 | -1.82836 | 2 |
| CCDC97 | 0.116848 | 2.160534 | 3.107499 | 0.002819 | 0.03748 | -1.83156 | 0 |
| QDPR | 0.119689 | 2.546272 | 3.10727 | 0.002821 | 0.03748 | -1.83216 | 2 |
| OXNAD1 | 0.114601 | 2.629595 | 3.102491 | 0.002861 | 0.037836 | -1.84465 | 2 |
| THAP6 | 0.105858 | 2.425504 | 3.094285 | 0.00293 | 0.038321 | -1.86605 | 3 |
| UBN1 | 0.137328 | 2.410243 | 3.091727 | 0.002952 | 0.0385 | -1.87272 | 0 |
| CYP20A1 | 0.113586 | 2.407843 | 3.089611 | 0.00297 | 0.038675 | -1.87823 | 3 |

| | | | | | | | |
|---|---|---|---|---|---|---|---|
| IGKC | 0.171047 | 2.324342 | 3.082336 | 0.003034 | 0.039155 | -1.89715 | 0 |
| ADHFE1 | 0.165266 | 2.918113 | 3.079809 | 0.003057 | 0.039358 | -1.90371 | 3 |
| FAM210A | 0.123137 | 2.71582 | 3.076631 | 0.003085 | 0.039579 | -1.91196 | 2 |
| CELF2 | 0.117462 | 2.835854 | 3.075839 | 0.003092 | 0.039629 | -1.91402 | 3 |
| TMEM69 | 0.124715 | 2.877128 | 3.075012 | 0.0031 | 0.039629 | -1.91616 | 2 |
| ZHX2 | 0.126575 | 2.894361 | 3.074939 | 0.0031 | 0.039629 | -1.91635 | 3 |
| TMEM14B | 0.117751 | 3.181631 | 3.059852 | 0.003239 | 0.040988 | -1.95542 | 2 |
| CDK19 | 0.107643 | 2.008306 | 3.056529 | 0.003271 | 0.041236 | -1.964 | 3 |
| CRLF3 | 0.136915 | 2.37911 | 3.053925 | 0.003296 | 0.04137 | -1.97073 | 3 |
| AFF3 | 0.129425 | 1.912024 | 3.051846 | 0.003316 | 0.041442 | -1.97609 | 1 |
| MRPS23 | 0.276718 | 2.844138 | 3.046744 | 0.003365 | 0.041761 | -1.98925 | 2 |
| PCCA | 0.180437 | 2.440228 | 3.042722 | 0.003404 | 0.042097 | -1.9996 | 2 |
| RCBTB2 | 0.130974 | 2.088411 | 3.031589 | 0.003516 | 0.043143 | -2.02822 | 1 |
| ZBTB40 | 0.139885 | 2.184834 | 3.014941 | 0.003689 | 0.044423 | -2.07087 | 3 |
| CALCOCO2 | 0.12258 | 3.24579 | 3.0124 | 0.003716 | 0.044615 | -2.07737 | 2 |
| PCID2 | 0.154379 | 2.528232 | 3.00704 | 0.003774 | 0.045117 | -2.09106 | 2 |
| TMEM251 | 0.150049 | 2.706217 | 3.00453 | 0.003801 | 0.045337 | -2.09746 | 2 |
| SLC25A3 | 0.207033 | 3.671008 | 2.995926 | 0.003896 | 0.045807 | -2.11938 | 2 |
| IKZF5 | 0.164481 | 2.366882 | 2.99279 | 0.003931 | 0.04606 | -2.12736 | 2 |
| LIAS | 0.116687 | 2.399073 | 2.99266 | 0.003933 | 0.04606 | -2.12769 | 3 |
| ATRN | 0.158282 | 2.475136 | 2.99089 | 0.003953 | 0.04617 | -2.13219 | 2 |
| PCMTD2 | 0.202719 | 2.736282 | 2.989409 | 0.003969 | 0.046305 | -2.13595 | 2 |
| ITPKB | 0.133563 | 1.88246 | 2.987445 | 0.003992 | 0.046442 | -2.14094 | 0 |
| CREB1 | 0.122557 | 2.763477 | 2.981648 | 0.004059 | 0.046938 | -2.15565 | 2 |
| TIMM44 | 0.129779 | 2.579289 | 2.974893 | 0.004138 | 0.047476 | -2.17277 | 2 |
| ELP5 | 0.112749 | 2.394788 | 2.973231 | 0.004157 | 0.047608 | -2.17697 | 2 |
| ACP1 | 0.112471 | 2.945194 | 2.969917 | 0.004197 | 0.047998 | -2.18536 | 2 |
| QRSL1 | 0.154407 | 2.513777 | 2.968397 | 0.004215 | 0.048126 | -2.1892 | 2 |
| PIGP | 0.123305 | 2.236694 | 2.966069 | 0.004243 | 0.048217 | -2.19508 | 2 |
| SEC11A | 0.185258 | 2.838386 | 2.961066 | 0.004304 | 0.048573 | -2.20771 | 2 |
| COX11 | 0.106915 | 2.422325 | 2.953978 | 0.004392 | 0.049089 | -2.22558 | 0 |
| TMBIM6 | 0.161958 | 3.532367 | 2.942987 | 0.004531 | 0.049895 | -2.25321 | 2 |
| FUNDC1 | 0.101059 | 2.793081 | 2.941426 | 0.004551 | 0.050085 | -2.25714 | 2 |
| KLHDC3 | 0.172601 | 2.681157 | 2.940874 | 0.004559 | 0.050132 | -2.25852 | 2 |
| RNF165 | 0.182613 | 1.948723 | 2.940346 | 0.004565 | 0.050176 | -2.25985 | 1 |
| HLTF | 0.127703 | 2.403795 | 2.937435 | 0.004603 | 0.050465 | -2.26715 | 2 |
| SLC25A36 | 0.125097 | 2.837288 | 2.930823 | 0.00469 | 0.050935 | -2.28372 | 3 |
| CLOCK | 0.171286 | 2.72596 | 2.923131 | 0.004793 | 0.051716 | -2.30295 | 2 |
| COX4I1 | 0.240863 | 3.43018 | 2.911376 | 0.004955 | 0.052792 | -2.33228 | 2 |
| SUCLG2 | 0.152758 | 2.93599 | 2.910715 | 0.004964 | 0.052792 | -2.33393 | 2 |
| STRBP | 0.123999 | 2.3577 | 2.906893 | 0.005018 | 0.053051 | -2.34344 | 0 |
| EIF3M | 0.263584 | 3.204997 | 2.903935 | 0.00506 | 0.053365 | -2.3508 | 2 |
| TMEM14C | 0.188571 | 3.228338 | 2.902845 | 0.005076 | 0.053426 | -2.35351 | 2 |
| ADK | 0.160815 | 2.59351 | 2.891273 | 0.005243 | 0.054413 | -2.38222 | 2 |
| CUL4A | 0.153327 | 2.993697 | 2.889072 | 0.005276 | 0.054588 | -2.38768 | 2 |
| KLRG1 | 0.137299 | 1.699859 | 2.883887 | 0.005353 | 0.05529 | -2.40051 | 1 |
| LPIN1 | 0.122083 | 2.520581 | 2.883254 | 0.005363 | 0.055355 | -2.40207 | 3 |
| THUMPD3 | 0.139107 | 2.837579 | 2.87525 | 0.005484 | 0.056344 | -2.42184 | 2 |
| RABGAP1L | 0.158255 | 2.580397 | 2.869293 | 0.005576 | 0.057055 | -2.43653 | 3 |
| COA3 | 0.120283 | 2.996007 | 2.869013 | 0.005581 | 0.057067 | -2.43722 | 2 |
| PARP1 | 0.198852 | 2.869731 | 2.864437 | 0.005652 | 0.057464 | -2.44849 | 2 |

| | | | | | | | |
|---|---|---|---|---|---|---|---|
| IP6K2 | 0.126822 | 2.400891 | 2.860735 | 0.005711 | 0.057926 | -2.45759 | 3 |
| ARL6IP1 | 0.191354 | 2.989869 | 2.857273 | 0.005767 | 0.058353 | -2.4661 | 2 |
| AKAP9 | 0.141766 | 2.632261 | 2.855214 | 0.0058 | 0.058553 | -2.47115 | 3 |
| GTF3C6 | 0.257869 | 2.820399 | 2.850346 | 0.005879 | 0.058993 | -2.48309 | 2 |
| FAM50B | 0.107329 | 1.936102 | 2.849947 | 0.005885 | 0.058993 | -2.48407 | 1 |
| THRA | 0.099332 | 2.926534 | 2.849818 | 0.005887 | 0.058993 | -2.48439 | 3 |
| AHSA1 | 0.229817 | 3.203238 | 2.847072 | 0.005933 | 0.05918 | -2.49112 | 2 |
| RMND1 | 0.118534 | 1.968747 | 2.84537 | 0.005961 | 0.05939 | -2.49528 | 0 |
| GAB1 | 0.16153 | 3.036059 | 2.839815 | 0.006053 | 0.059854 | -2.50887 | 3 |
| PPP1R12B | 0.193328 | 3.284948 | 2.838771 | 0.006071 | 0.059909 | -2.51142 | 2 |
| HSD17B8 | 0.142673 | 2.476452 | 2.820785 | 0.006381 | 0.061903 | -2.55527 | 2 |
| LRPPRC | 0.192361 | 3.065342 | 2.820693 | 0.006383 | 0.061903 | -2.55549 | 2 |
| DEXI | 0.090741 | 3.354925 | 2.818431 | 0.006423 | 0.062119 | -2.56099 | 0 |
| CTCF | 0.182856 | 2.732762 | 2.814463 | 0.006494 | 0.062598 | -2.57063 | 2 |
| GOT2 | 0.192152 | 3.307794 | 2.803678 | 0.00669 | 0.064027 | -2.59677 | 2 |
| TNFRSF8 | 0.114303 | 1.771117 | 2.79516 | 0.006848 | 0.064941 | -2.61736 | 0 |
| CCSER2 | 0.119484 | 3.048356 | 2.793384 | 0.006882 | 0.065046 | -2.62165 | 3 |
| SIAH1 | 0.07683 | 2.411959 | 2.785652 | 0.00703 | 0.066157 | -2.64029 | 0 |
| HAX1 | 0.139002 | 3.272702 | 2.783177 | 0.007078 | 0.066429 | -2.64625 | 2 |
| NDUFS1 | 0.190903 | 3.433036 | 2.775225 | 0.007233 | 0.067507 | -2.66537 | 2 |
| DIS3L | 0.112745 | 2.462246 | 2.77354 | 0.007267 | 0.067661 | -2.66941 | 2 |
| LSS | 0.115338 | 2.266019 | 2.762814 | 0.007483 | 0.068831 | -2.69512 | 0 |
| ABR | 0.101133 | 2.980999 | 2.762147 | 0.007497 | 0.068898 | -2.69672 | 3 |
| OCEL1 | 0.116641 | 1.964109 | 2.748455 | 0.007782 | 0.070759 | -2.72941 | 0 |
| ACSS1 | 0.192415 | 2.568502 | 2.74547 | 0.007845 | 0.071114 | -2.73652 | 2 |
| RPL30 | 0.097348 | 3.828421 | 2.743661 | 0.007884 | 0.071354 | -2.74083 | 2 |
| MDH2 | 0.139131 | 3.72337 | 2.738522 | 0.007995 | 0.072059 | -2.75305 | 2 |
| BCS1L | 0.086124 | 2.546214 | 2.731888 | 0.00814 | 0.072842 | -2.76881 | 2 |
| FOLR2 | 0.15131 | 2.521052 | 2.730867 | 0.008162 | 0.073006 | -2.77123 | 0 |
| TMEM135 | 0.112957 | 2.210281 | 2.724215 | 0.008311 | 0.07403 | -2.78699 | 2 |
| MRPL45 | 0.208277 | 2.893061 | 2.721723 | 0.008367 | 0.07432 | -2.79288 | 2 |
| ASF1B | 0.103319 | 1.857291 | 2.720894 | 0.008386 | 0.07432 | -2.79484 | 0 |
| RNF157 | 0.130422 | 1.927239 | 2.718803 | 0.008433 | 0.074573 | -2.79979 | 0 |
| CLASP2 | 0.095651 | 2.739679 | 2.718253 | 0.008446 | 0.074573 | -2.80109 | 3 |
| PPID | 0.199192 | 2.930103 | 2.714339 | 0.008536 | 0.075115 | -2.81033 | 2 |
| DFFB | 0.084274 | 1.963695 | 2.700219 | 0.008867 | 0.076618 | -2.84359 | 0 |
| WDR7 | 0.159246 | 2.641561 | 2.694563 | 0.009003 | 0.077428 | -2.85687 | 3 |
| MRPL37 | 0.122455 | 3.22701 | 2.691027 | 0.009089 | 0.077789 | -2.86517 | 2 |
| RNASET2 | 0.095456 | 1.923643 | 2.688044 | 0.009162 | 0.078033 | -2.87216 | 0 |
| RPRD2 | 0.134701 | 2.787848 | 2.683415 | 0.009276 | 0.078624 | -2.88299 | 3 |
| STYX | 0.086763 | 2.708796 | 2.677008 | 0.009437 | 0.079575 | -2.89797 | 2 |
| PAFAH1B1 | 0.129398 | 3.096946 | 2.676411 | 0.009452 | 0.07965 | -2.89936 | 2 |
| RPS27A | 0.122614 | 3.463095 | 2.676034 | 0.009461 | 0.079692 | -2.90024 | 2 |
| NDUFS7 | 0.110332 | 3.085312 | 2.673046 | 0.009537 | 0.0801 | -2.90721 | 2 |
| MCCC2 | 0.155286 | 2.803713 | 2.661419 | 0.009838 | 0.08184 | -2.93428 | 2 |
| BOLA3 | 0.119693 | 3.313762 | 2.660557 | 0.009861 | 0.08199 | -2.93628 | 2 |
| PEPD | 0.086268 | 2.482514 | 2.659983 | 0.009876 | 0.082076 | -2.93761 | 2 |
| C11orf21 | 0.125789 | 2.1441 | 2.655207 | 0.010002 | 0.08271 | -2.9487 | 1 |
| CEP85L | 0.092467 | 2.063016 | 2.654488 | 0.010021 | 0.082775 | -2.95037 | 0 |
| MLYCD | 0.187641 | 3.050357 | 2.649681 | 0.01015 | 0.083408 | -2.96151 | 2 |
| STT3B | 0.145248 | 2.975803 | 2.646012 | 0.01025 | 0.083988 | -2.97 | 2 |

| | | | | | | | |
|---|---|---|---|---|---|---|---|
| ERMP1 | 0.133531 | 2.4825 | 2.645579 | 0.010262 | 0.08402 | -2.971 | 2 |
| PDHX | 0.158436 | 3.006824 | 2.639786 | 0.010421 | 0.084911 | -2.98439 | 2 |
| PDHA1 | 0.146359 | 3.442166 | 2.639137 | 0.010439 | 0.085018 | -2.98589 | 2 |
| TOP2B | 0.173004 | 2.980379 | 2.636484 | 0.010513 | 0.08518 | -2.99201 | 2 |
| SLC35A4 | 0.088331 | 2.904924 | 2.631496 | 0.010652 | 0.085957 | -3.00351 | 2 |
| MAPRE2 | 0.113322 | 3.200043 | 2.627528 | 0.010765 | 0.086588 | -3.01264 | 3 |
| GPD1L | 0.187914 | 3.235516 | 2.627515 | 0.010765 | 0.086588 | -3.01267 | 2 |
| ARMCX3 | 0.099735 | 2.807144 | 2.618781 | 0.011017 | 0.087602 | -3.03274 | 2 |
| DCTPP1 | 0.129176 | 2.685931 | 2.610443 | 0.011262 | 0.088823 | -3.05184 | 2 |
| DUSP10 | 0.139955 | 1.803691 | 2.609605 | 0.011287 | 0.088979 | -3.05376 | 1 |
| PHAX | 0.121765 | 2.34109 | 2.608587 | 0.011317 | 0.089058 | -3.05609 | 2 |
| RASGRP2 | 0.107917 | 2.025779 | 2.603027 | 0.011484 | 0.089765 | -3.0688 | 0 |
| NNT | 0.196925 | 3.398639 | 2.601673 | 0.011525 | 0.089924 | -3.07189 | 2 |
| LRBA | 0.132629 | 2.473149 | 2.593742 | 0.011767 | 0.091116 | -3.08997 | 2 |
| HTATSF1 | 0.124188 | 2.991674 | 2.593183 | 0.011785 | 0.091139 | -3.09124 | 2 |
| CNOT7 | 0.091389 | 2.742667 | 2.590312 | 0.011874 | 0.091383 | -3.09777 | 2 |
| PTGDR | 0.119746 | 1.688827 | 2.589259 | 0.011906 | 0.091595 | -3.10017 | 1 |
| PCNA | 0.147945 | 2.402315 | 2.574029 | 0.012391 | 0.094078 | -3.13471 | 2 |
| NDRG2 | 0.10931 | 3.501887 | 2.571532 | 0.012472 | 0.094447 | -3.14036 | 2 |
| SLC41A3 | 0.069824 | 2.557569 | 2.56753 | 0.012603 | 0.095068 | -3.14941 | 0 |
| IRF8 | 0.110597 | 1.738185 | 2.565322 | 0.012675 | 0.09537 | -3.15439 | 0 |
| EMC4 | 0.119391 | 2.84669 | 2.56333 | 0.012741 | 0.095619 | -3.15888 | 2 |
| RFC5 | 0.088406 | 1.804632 | 2.561639 | 0.012797 | 0.095809 | -3.1627 | 0 |
| METTL17 | 0.092886 | 2.669751 | 2.560616 | 0.012832 | 0.095927 | -3.165 | 2 |
| PKN2 | 0.146456 | 2.776431 | 2.559121 | 0.012882 | 0.096096 | -3.16837 | 3 |
| POLR3B | 0.093438 | 2.276416 | 2.551074 | 0.013154 | 0.097462 | -3.18647 | 0 |
| ABLIM1 | 0.165346 | 3.609427 | 2.550039 | 0.013189 | 0.097593 | -3.1888 | 3 |
| COG4 | 0.109121 | 2.523767 | 2.547906 | 0.013263 | 0.097852 | -3.19359 | 2 |
| IL2RA | 0.132962 | 1.828072 | 2.54203 | 0.013466 | 0.098659 | -3.20676 | 0 |
| LYSMD4 | 0.084382 | 1.760097 | 2.54033 | 0.013526 | 0.098971 | -3.21057 | 0 |
| COPRS | 0.104033 | 2.822041 | 2.533757 | 0.013758 | 0.099811 | -3.22527 | 2 |
| COQ9 | 0.135605 | 3.242293 | 2.533476 | 0.013768 | 0.099811 | -3.2259 | 2 |
| SPIN1 | 0.084061 | 2.746676 | 2.532003 | 0.01382 | 0.100026 | -3.22919 | 3 |
| VDAC1 | 0.119546 | 3.57687 | 2.516028 | 0.014402 | 0.102914 | -3.26477 | 2 |
| FCGRT | 0.09378 | 2.576469 | 2.514924 | 0.014443 | 0.10308 | -3.26722 | 0 |
| RPL14 | 0.128297 | 3.981148 | 2.510269 | 0.014617 | 0.103856 | -3.27756 | 2 |
| TMEM147 | 0.144722 | 3.12603 | 2.508052 | 0.0147 | 0.104101 | -3.28247 | 2 |
| C17orf80 | 0.104458 | 2.195411 | 2.505845 | 0.014784 | 0.104533 | -3.28736 | 2 |
| TIMM50 | 0.149296 | 3.058831 | 2.504904 | 0.01482 | 0.10472 | -3.28944 | 2 |
| PSEN2 | 0.087322 | 2.122441 | 2.504074 | 0.014851 | 0.104777 | -3.29128 | 0 |
| LACTB2 | 0.124669 | 2.456978 | 2.501454 | 0.014951 | 0.105039 | -3.29708 | 2 |
| BTD | 0.088479 | 1.970009 | 2.500067 | 0.015005 | 0.105202 | -3.30014 | 1 |
| ERP29 | 0.097666 | 2.303969 | 2.497924 | 0.015087 | 0.10557 | -3.30488 | 0 |
| UQCR11 | 0.173299 | 3.906979 | 2.49709 | 0.01512 | 0.105669 | -3.30672 | 2 |
| ADCY9 | 0.14471 | 2.61812 | 2.48974 | 0.015407 | 0.106949 | -3.32293 | 0 |
| MRPS27 | 0.094663 | 2.74654 | 2.489005 | 0.015436 | 0.107065 | -3.32455 | 2 |
| NAGK | 0.060996 | 2.598121 | 2.488052 | 0.015474 | 0.107199 | -3.32665 | 0 |
| CLIC3 | 0.14667 | 1.956392 | 2.487425 | 0.015498 | 0.107328 | -3.32803 | 1 |
| ZMYM3 | 0.104358 | 2.066752 | 2.486135 | 0.01555 | 0.107512 | -3.33087 | 1 |
| NF2 | 0.097361 | 2.32217 | 2.47645 | 0.015939 | 0.109457 | -3.35214 | 0 |
| TMEM186 | 0.103931 | 2.104324 | 2.476179 | 0.01595 | 0.109457 | -3.35273 | 2 |

| | | | | | | | |
|---|---|---|---|---|---|---|---|
| PASK | 0.088764 | 1.619231 | 2.475409 | 0.015981 | 0.109505 | -3.35442 | 0 |
| KRBOX4 | 0.099434 | 2.13882 | 2.474644 | 0.016012 | 0.109565 | -3.3561 | 0 |
| WDR92 | 0.081427 | 1.84158 | 2.473814 | 0.016046 | 0.109571 | -3.35792 | 0 |
| LSM5 | 0.099195 | 2.432542 | 2.473519 | 0.016058 | 0.109571 | -3.35857 | 2 |
| TMEM187 | 0.130383 | 2.127389 | 2.47255 | 0.016098 | 0.10966 | -3.36069 | 0 |
| NUDT16L1 | 0.123033 | 2.627907 | 2.468319 | 0.016272 | 0.110114 | -3.36995 | 1 |
| EID1 | 0.098465 | 3.377868 | 2.464959 | 0.016412 | 0.110759 | -3.37729 | 2 |
| NFXL1 | 0.136717 | 2.15186 | 2.463642 | 0.016467 | 0.111002 | -3.38017 | 0 |
| ORC2 | 0.090771 | 2.510787 | 2.463104 | 0.016489 | 0.111103 | -3.38135 | 3 |
| DAD1 | 0.162134 | 3.400575 | 2.461166 | 0.016571 | 0.111401 | -3.38558 | 2 |
| USP16 | 0.14662 | 2.900424 | 2.460169 | 0.016613 | 0.111512 | -3.38775 | 2 |
| RTCB | 0.153196 | 2.75644 | 2.456535 | 0.016767 | 0.11233 | -3.39567 | 2 |
| OCIAD1 | 0.096632 | 2.895143 | 2.454854 | 0.016838 | 0.112608 | -3.39934 | 2 |
| TMEM159 | 0.112017 | 2.944012 | 2.451605 | 0.016977 | 0.113092 | -3.40641 | 2 |
| ITGA6 | 0.085772 | 2.908539 | 2.449703 | 0.017059 | 0.113402 | -3.41054 | 0 |
| KCNAB2 | 0.124218 | 2.008841 | 2.446776 | 0.017186 | 0.113919 | -3.4169 | 0 |
| COG1 | 0.099873 | 2.907778 | 2.442213 | 0.017386 | 0.114546 | -3.4268 | 2 |
| SMG8 | 0.10602 | 1.922293 | 2.44044 | 0.017464 | 0.11481 | -3.43065 | 0 |
| ACTR8 | 0.106754 | 2.512283 | 2.43712 | 0.017611 | 0.11555 | -3.43784 | 2 |
| AFG3L2 | 0.181445 | 3.112587 | 2.436908 | 0.01762 | 0.115568 | -3.4383 | 2 |
| GOT1 | 0.200769 | 3.726626 | 2.430181 | 0.017921 | 0.116931 | -3.45284 | 2 |
| SCAP | 0.098902 | 2.664285 | 2.428174 | 0.018012 | 0.117319 | -3.45718 | 0 |
| CUL2 | 0.114691 | 2.51193 | 2.42789 | 0.018025 | 0.117319 | -3.45779 | 2 |
| TNS3 | 0.10265 | 2.24742 | 2.421649 | 0.01831 | 0.118549 | -3.47124 | 0 |
| SP4 | 0.081084 | 2.413987 | 2.420901 | 0.018345 | 0.118697 | -3.47285 | 3 |
| NAA20 | 0.10662 | 2.487308 | 2.419628 | 0.018404 | 0.118923 | -3.47559 | 2 |
| SNRPF | 0.087349 | 2.772881 | 2.413981 | 0.018666 | 0.119849 | -3.48773 | 2 |
| NDUFA12 | 0.142699 | 3.543342 | 2.412549 | 0.018733 | 0.119929 | -3.49081 | 2 |
| VEZF1 | 0.193594 | 3.171038 | 2.408264 | 0.018936 | 0.120623 | -3.5 | 3 |
| UBA5 | 0.081507 | 2.673063 | 2.401276 | 0.01927 | 0.12196 | -3.51496 | 2 |
| TMEM106B | 0.084309 | 2.769876 | 2.399086 | 0.019375 | 0.122403 | -3.51964 | 2 |
| HIC2 | 0.103229 | 2.128662 | 2.396697 | 0.019491 | 0.122552 | -3.52474 | 0 |
| CNST | 0.096308 | 2.186573 | 2.396463 | 0.019503 | 0.122552 | -3.52524 | 0 |
| MPC1 | 0.158832 | 3.252018 | 2.396343 | 0.019509 | 0.122552 | -3.5255 | 2 |
| INIP | 0.098429 | 2.446775 | 2.392442 | 0.019699 | 0.123509 | -3.53382 | 0 |
| SMYD3 | 0.085645 | 1.814692 | 2.388604 | 0.019889 | 0.124184 | -3.542 | 0 |
| WLS | 0.091329 | 3.003925 | 2.381842 | 0.020226 | 0.125597 | -3.55638 | 2 |
| DLST | 0.138976 | 3.090497 | 2.377073 | 0.020468 | 0.12642 | -3.5665 | 2 |
| ZNF830 | 0.130261 | 2.411475 | 2.375641 | 0.020541 | 0.126693 | -3.56953 | 2 |
| PPM1K | 0.171915 | 2.807557 | 2.374311 | 0.020609 | 0.126977 | -3.57235 | 3 |
| PSMC5 | 0.141885 | 3.364579 | 2.371339 | 0.020761 | 0.127522 | -3.57864 | 2 |
| GALNT11 | 0.071947 | 2.318273 | 2.370461 | 0.020806 | 0.127522 | -3.5805 | 1 |
| LCLAT1 | 0.125591 | 2.725545 | 2.369271 | 0.020868 | 0.127719 | -3.58302 | 2 |
| ALDH1A1 | 0.135315 | 2.238683 | 2.368343 | 0.020916 | 0.127969 | -3.58498 | 0 |
| CASP8AP2 | 0.095834 | 2.206133 | 2.368156 | 0.020926 | 0.127983 | -3.58538 | 0 |
| KANK1 | 0.104106 | 2.756464 | 2.367515 | 0.020959 | 0.128142 | -3.58673 | 3 |
| ZSCAN26 | 0.086493 | 2.176855 | 2.363589 | 0.021164 | 0.129078 | -3.59502 | 1 |
| GFM1 | 0.144279 | 2.788445 | 2.36202 | 0.021246 | 0.129486 | -3.59833 | 2 |
| RBM17 | 0.107913 | 2.778819 | 2.361525 | 0.021272 | 0.129513 | -3.59938 | 2 |
| OXA1L | 0.128743 | 3.341978 | 2.361209 | 0.021289 | 0.129569 | -3.60004 | 2 |
| CYTH3 | 0.077523 | 2.093269 | 2.357277 | 0.021497 | 0.130555 | -3.60833 | 0 |

| | | | | | | | |
|---|---|---|---|---|---|---|---|
| COX20 | 0.140858 | 2.583581 | 2.357049 | 0.021509 | 0.130555 | -3.60881 | 2 |
| SYPL1 | 0.102996 | 3.028535 | 2.356659 | 0.021529 | 0.13058 | -3.60963 | 2 |
| SAMM50 | 0.107944 | 2.80697 | 2.348629 | 0.02196 | 0.13223 | -3.62651 | 2 |
| EIF3K | 0.170272 | 3.800126 | 2.347635 | 0.022014 | 0.132509 | -3.6286 | 2 |
| NDUFB8 | 0.047835 | 2.956469 | 2.347233 | 0.022036 | 0.132549 | -3.62944 | 2 |
| NUDT9 | 0.096369 | 2.447299 | 2.347028 | 0.022047 | 0.13255 | -3.62987 | 2 |
| PEBP1 | 0.129636 | 3.654843 | 2.345874 | 0.02211 | 0.132765 | -3.63229 | 2 |
| SLC38A7 | 0.073196 | 2.34195 | 2.339282 | 0.022471 | 0.134381 | -3.6461 | 0 |
| SH3BP1 | 0.078739 | 1.875828 | 2.338366 | 0.022522 | 0.134594 | -3.64802 | 0 |
| RPL22 | 0.108773 | 3.071321 | 2.337282 | 0.022582 | 0.134769 | -3.65029 | 2 |
| CYP4V2 | 0.114064 | 2.549548 | 2.336222 | 0.022641 | 0.135028 | -3.6525 | 1 |
| ARHGAP25 | 0.102684 | 1.887943 | 2.33429 | 0.022749 | 0.135255 | -3.65654 | 1 |
| GRHPR | 0.078685 | 3.052353 | 2.332661 | 0.02284 | 0.135521 | -3.65994 | 2 |
| GPBAR1 | 0.103295 | 1.835423 | 2.331907 | 0.022882 | 0.135588 | -3.66152 | 1 |
| KIAA1671 | 0.10298 | 2.258289 | 2.327238 | 0.023146 | 0.136686 | -3.67125 | 1 |
| KLHDC2 | 0.148294 | 2.991646 | 2.326621 | 0.023181 | 0.136822 | -3.67254 | 2 |
| TACC1 | 0.085893 | 3.006957 | 2.323581 | 0.023354 | 0.137313 | -3.67886 | 0 |
| TSFM | 0.098889 | 2.528555 | 2.320419 | 0.023536 | 0.137962 | -3.68544 | 2 |
| SAP18 | 0.114898 | 3.272344 | 2.320127 | 0.023552 | 0.138015 | -3.68605 | 2 |
| SUPT7L | 0.127792 | 2.727191 | 2.316731 | 0.023749 | 0.138886 | -3.6931 | 2 |
| WWP1 | 0.13437 | 3.019965 | 2.31635 | 0.023771 | 0.138922 | -3.69389 | 2 |
| FXN | 0.084743 | 2.524916 | 2.314269 | 0.023892 | 0.139351 | -3.69821 | 2 |
| MCM3AP | 0.087124 | 2.44591 | 2.313688 | 0.023926 | 0.139456 | -3.69941 | 0 |
| LGR6 | 0.080253 | 1.666318 | 2.307083 | 0.024315 | 0.14058 | -3.71309 | 0 |
| PPWD1 | 0.09916 | 2.53679 | 2.305883 | 0.024386 | 0.140822 | -3.71557 | 2 |
| DTYMK | 0.080684 | 2.010945 | 2.305556 | 0.024405 | 0.140848 | -3.71625 | 0 |
| VPS45 | 0.088335 | 2.755664 | 2.305402 | 0.024414 | 0.140848 | -3.71657 | 2 |
| MTX3 | 0.07765 | 2.544024 | 2.30064 | 0.024699 | 0.142021 | -3.7264 | 2 |
| DPP4 | 0.16983 | 1.915577 | 2.29737 | 0.024896 | 0.142593 | -3.73314 | 0 |
| SLC7A7 | 0.086035 | 1.933584 | 2.291728 | 0.02524 | 0.14414 | -3.74476 | 0 |
| DZIP3 | 0.070277 | 2.482023 | 2.282971 | 0.025781 | 0.146272 | -3.76274 | 3 |
| SLC25A33 | 0.078998 | 2.063065 | 2.279956 | 0.02597 | 0.147097 | -3.76892 | 3 |
| ZNF33B | 0.106208 | 2.258471 | 2.279194 | 0.026018 | 0.147281 | -3.77048 | 0 |
| DROSHA | 0.099856 | 2.594435 | 2.276499 | 0.026188 | 0.147956 | -3.77599 | 3 |
| SESN1 | 0.127535 | 2.807956 | 2.275156 | 0.026273 | 0.148198 | -3.77874 | 3 |
| RPL15 | 0.116119 | 3.489631 | 2.272245 | 0.026459 | 0.149003 | -3.78469 | 2 |
| BBS10 | 0.082935 | 2.290847 | 2.269557 | 0.026631 | 0.149675 | -3.79017 | 3 |
| NSA2 | 0.163517 | 3.148003 | 2.262098 | 0.027114 | 0.151424 | -3.80537 | 2 |
| PANK4 | 0.072431 | 2.171277 | 2.26152 | 0.027151 | 0.151585 | -3.80654 | 0 |
| POLB | 0.077317 | 2.513855 | 2.260136 | 0.027242 | 0.151947 | -3.80936 | 0 |
| SLC16A7 | 0.115745 | 2.979719 | 2.259583 | 0.027278 | 0.152101 | -3.81048 | 2 |
| METTL21A | 0.063921 | 2.405883 | 2.259267 | 0.027299 | 0.152129 | -3.81112 | 0 |
| CHTOP | 0.084988 | 2.861375 | 2.256746 | 0.027465 | 0.152419 | -3.81624 | 2 |
| VAMP8 | 0.137628 | 2.316863 | 2.256731 | 0.027466 | 0.152419 | -3.81628 | 0 |
| ZNF605 | 0.092954 | 1.917236 | 2.255895 | 0.027521 | 0.152569 | -3.81797 | 0 |
| COX18 | 0.079273 | 2.352784 | 2.25582 | 0.027526 | 0.152569 | -3.81812 | 0 |
| IFT27 | 0.083296 | 2.452424 | 2.254652 | 0.027604 | 0.152697 | -3.82049 | 0 |
| RAD21 | 0.121099 | 3.130791 | 2.253443 | 0.027684 | 0.152948 | -3.82295 | 2 |
| TBC1D5 | 0.079297 | 2.35489 | 2.249222 | 0.027966 | 0.15397 | -3.8315 | 0 |
| PNPO | 0.092307 | 2.214894 | 2.248529 | 0.028012 | 0.15408 | -3.8329 | 2 |
| NR1D2 | 0.146708 | 2.329597 | 2.248339 | 0.028025 | 0.154102 | -3.83328 | 3 |

| | | | | | | | |
|---|---|---|---|---|---|---|---|
| GCDH | 0.080658 | 2.27641 | 2.247158 | 0.028104 | 0.154442 | -3.83568 | 0 |
| CTSS | 0.16026 | 2.582899 | 2.244901 | 0.028257 | 0.154987 | -3.84024 | 0 |
| PRIM1 | 0.120355 | 1.773657 | 2.244216 | 0.028303 | 0.155096 | -3.84162 | 0 |
| ADNP2 | 0.0932 | 1.827512 | 2.243902 | 0.028325 | 0.155131 | -3.84226 | 0 |
| INTS7 | 0.117063 | 2.020719 | 2.243859 | 0.028327 | 0.155131 | -3.84234 | 0 |
| MADD | 0.098309 | 2.626144 | 2.242774 | 0.028401 | 0.155404 | -3.84453 | 0 |
| TBC1D4 | 0.134549 | 2.92429 | 2.236162 | 0.028854 | 0.157094 | -3.85787 | 3 |
| CDKN1B | 0.061928 | 2.886158 | 2.233998 | 0.029003 | 0.157619 | -3.86223 | 3 |
| ALS2 | 0.101278 | 2.519752 | 2.233944 | 0.029007 | 0.157619 | -3.86234 | 3 |
| ATG2A | 0.07439 | 2.116804 | 2.232457 | 0.02911 | 0.157873 | -3.86533 | 0 |
| CSF1R | 0.10182 | 2.461803 | 2.231122 | 0.029203 | 0.158046 | -3.86802 | 1 |
| HSPA9 | 0.130444 | 3.50689 | 2.229726 | 0.029301 | 0.158387 | -3.87082 | 2 |
| CNBP | 0.128937 | 3.478605 | 2.228891 | 0.029359 | 0.158546 | -3.8725 | 2 |
| TRIM65 | 0.090833 | 2.071554 | 2.217171 | 0.03019 | 0.161141 | -3.89599 | 0 |
| NUP210 | 0.071686 | 1.790728 | 2.21643 | 0.030243 | 0.161179 | -3.89747 | 0 |
| NIPAL3 | 0.092326 | 2.086584 | 2.215633 | 0.0303 | 0.161337 | -3.89907 | 0 |
| OARD1 | 0.067923 | 2.67492 | 2.212746 | 0.030509 | 0.162052 | -3.90483 | 2 |
| CBFA2T3 | 0.094079 | 2.083558 | 2.212136 | 0.030553 | 0.162238 | -3.90605 | 1 |
| RGCC | 0.102183 | 2.988441 | 2.208871 | 0.03079 | 0.16261 | -3.91257 | 0 |
| RIC8B | 0.065878 | 1.977025 | 2.207042 | 0.030924 | 0.163074 | -3.91621 | 0 |
| HADHA | 0.18732 | 3.60758 | 2.2041 | 0.03114 | 0.163668 | -3.92207 | 2 |
| ZNF180 | 0.099293 | 1.982771 | 2.202805 | 0.031236 | 0.164022 | -3.92464 | 0 |
| ENDOG | 0.093896 | 3.164522 | 2.199894 | 0.031452 | 0.164612 | -3.93043 | 0 |
| ECI1 | 0.084444 | 3.091853 | 2.196416 | 0.031712 | 0.165082 | -3.93734 | 2 |
| MRPS9 | 0.138366 | 2.832051 | 2.19514 | 0.031807 | 0.165223 | -3.93987 | 2 |
| NINJ1 | 0.08917 | 2.776816 | 2.192965 | 0.031971 | 0.165644 | -3.94418 | 0 |
| LIN52 | 0.099624 | 2.127333 | 2.192458 | 0.032009 | 0.165793 | -3.94518 | 3 |
| PPTC7 | 0.103925 | 3.311881 | 2.189406 | 0.032241 | 0.166551 | -3.95122 | 2 |
| MRPS35 | 0.161433 | 2.972334 | 2.184464 | 0.032618 | 0.167897 | -3.96099 | 2 |
| ATRX | 0.127246 | 2.845813 | 2.182375 | 0.032779 | 0.168531 | -3.96512 | 3 |
| COX5A | 0.163697 | 3.987628 | 2.181995 | 0.032808 | 0.168583 | -3.96587 | 2 |
| DHPS | 0.132061 | 2.727201 | 2.179621 | 0.032992 | 0.169178 | -3.97055 | 2 |
| CENPV | 0.079261 | 2.081695 | 2.176386 | 0.033244 | 0.17022 | -3.97692 | 0 |
| ZNF92 | 0.090123 | 1.92966 | 2.175484 | 0.033314 | 0.170431 | -3.9787 | 2 |
| PTPRM | 0.118612 | 3.289562 | 2.172545 | 0.033545 | 0.171145 | -3.98448 | 2 |
| PCYOX1L | 0.079878 | 1.986267 | 2.166567 | 0.034018 | 0.172819 | -3.99621 | 0 |
| METTL15 | 0.101393 | 2.068956 | 2.156512 | 0.034828 | 0.175658 | -4.01589 | 0 |
| RPS23 | 0.150169 | 3.053889 | 2.156059 | 0.034865 | 0.17579 | -4.01678 | 2 |
| DDHD2 | 0.065286 | 2.683995 | 2.152138 | 0.035185 | 0.176846 | -4.02443 | 3 |
| PEX19 | 0.084791 | 2.805193 | 2.149039 | 0.03544 | 0.177581 | -4.03047 | 2 |
| RBM14 | 0.109777 | 2.273938 | 2.149003 | 0.035443 | 0.177581 | -4.03054 | 0 |
| REEP5 | 0.086339 | 3.356712 | 2.146591 | 0.035643 | 0.178071 | -4.03523 | 2 |
| NIPAL2 | 0.075495 | 2.069716 | 2.141869 | 0.036037 | 0.179308 | -4.04441 | 0 |
| DCAF7 | 0.108581 | 2.808208 | 2.138151 | 0.036349 | 0.179993 | -4.05162 | 2 |
| GALT | 0.069081 | 2.583799 | 2.137712 | 0.036386 | 0.180042 | -4.05247 | 0 |
| MRPL21 | 0.156177 | 3.111296 | 2.137579 | 0.036398 | 0.180042 | -4.05273 | 2 |
| ZNF17 | 0.08165 | 1.843032 | 2.13648 | 0.036491 | 0.18031 | -4.05486 | 0 |
| ZNF703 | 0.114522 | 2.303886 | 2.134864 | 0.036628 | 0.180669 | -4.05799 | 0 |
| NDUFV1 | 0.116807 | 3.57734 | 2.127546 | 0.037254 | 0.182831 | -4.07214 | 2 |
| CDC42SE2 | 0.073072 | 2.277398 | 2.126964 | 0.037305 | 0.182975 | -4.07326 | 0 |
| HIBADH | 0.095565 | 3.068816 | 2.125223 | 0.037455 | 0.183405 | -4.07662 | 2 |

| | | | | | | | |
|---|---|---|---|---|---|---|---|
| DAAM1 | 0.172836 | 2.8337 | 2.120533 | 0.037864 | 0.184349 | -4.08566 | 3 |
| OPA3 | 0.062754 | 2.11491 | 2.116594 | 0.038209 | 0.185489 | -4.09324 | 0 |
| GNPTG | 0.056604 | 2.801763 | 2.115904 | 0.03827 | 0.185681 | -4.09456 | 0 |
| ZNF268 | 0.0708 | 1.647631 | 2.113684 | 0.038467 | 0.186047 | -4.09883 | 0 |
| RPS6KA5 | 0.100303 | 1.72055 | 2.111828 | 0.038632 | 0.186452 | -4.10239 | 0 |
| POLR2I | 0.141465 | 3.199831 | 2.109137 | 0.038872 | 0.187352 | -4.10755 | 2 |
| GALK2 | 0.064999 | 2.201503 | 2.102943 | 0.03943 | 0.188948 | -4.1194 | 0 |
| SCAPER | 0.123569 | 2.788183 | 2.100434 | 0.039658 | 0.189728 | -4.12419 | 3 |
| OXCT1 | 0.158757 | 3.145048 | 2.099484 | 0.039744 | 0.189934 | -4.12601 | 2 |
| THUMPD1 | 0.122044 | 2.37069 | 2.098834 | 0.039804 | 0.190166 | -4.12725 | 0 |
| AMD1 | 0.153143 | 2.835191 | 2.092028 | 0.04043 | 0.191795 | -4.14022 | 2 |
| CD99 | 0.104684 | 3.516035 | 2.08878 | 0.040732 | 0.192287 | -4.14639 | 0 |
| HARS2 | 0.078149 | 2.249633 | 2.082331 | 0.041337 | 0.19448 | -4.15863 | 2 |
| RAD17 | 0.080858 | 2.345548 | 2.077742 | 0.041772 | 0.195911 | -4.16732 | 2 |
| BCCIP | 0.152434 | 2.744602 | 2.076995 | 0.041843 | 0.196 | -4.16873 | 2 |
| BRWD1 | 0.110494 | 2.610245 | 2.074289 | 0.042102 | 0.196843 | -4.17384 | 3 |
| KRTAP10-1 | 0.109588 | 1.807333 | 2.068919 | 0.04262 | 0.198058 | -4.18397 | 1 |
| SMPD1 | 0.098283 | 2.587741 | 2.065299 | 0.042972 | 0.198838 | -4.19079 | 2 |
| PSMC3 | 0.142106 | 3.156664 | 2.064021 | 0.043097 | 0.199062 | -4.1932 | 2 |
| GAL3ST4 | 0.095938 | 1.880032 | 2.061544 | 0.04334 | 0.199802 | -4.19785 | 0 |
| ARFGEF2 | 0.128766 | 2.91966 | 2.05805 | 0.043685 | 0.200914 | -4.20441 | 2 |
| HSF2 | 0.075183 | 2.526367 | 2.052171 | 0.04427 | 0.202541 | -4.21542 | 0 |
| IRF2BP2 | 0.127253 | 2.795693 | 2.049395 | 0.044549 | 0.203241 | -4.22062 | 3 |
| EHBP1 | 0.063193 | 2.394875 | 2.049377 | 0.044551 | 0.203241 | -4.22065 | 0 |
| SLC7A6OS | 0.093913 | 2.830012 | 2.041293 | 0.045372 | 0.205697 | -4.23573 | 2 |
| USP28 | 0.149102 | 3.060994 | 2.041013 | 0.045401 | 0.20572 | -4.23625 | 3 |
| PDE12 | 0.088158 | 2.121777 | 2.038581 | 0.04565 | 0.20653 | -4.24078 | 2 |
| MRPL54 | 0.125524 | 2.712292 | 2.036009 | 0.045915 | 0.207034 | -4.24556 | 2 |
| TRUB2 | 0.100265 | 2.588363 | 2.035271 | 0.045992 | 0.207325 | -4.24693 | 2 |
| SMU1 | 0.130653 | 2.654161 | 2.033894 | 0.046135 | 0.207754 | -4.24949 | 2 |
| YWHAG | 0.108613 | 3.236771 | 2.029296 | 0.046614 | 0.209267 | -4.25802 | 2 |
| ATP2A2 | 0.172198 | 3.999603 | 2.027949 | 0.046755 | 0.209632 | -4.26052 | 2 |
| NTHL1 | 0.081717 | 2.01259 | 2.023134 | 0.047263 | 0.21099 | -4.26942 | 0 |
| FGL2 | 0.112938 | 2.321386 | 2.021274 | 0.047461 | 0.211651 | -4.27286 | 0 |
| HFE | 0.078119 | 1.752017 | 2.02117 | 0.047472 | 0.211651 | -4.27305 | 1 |
| PACS2 | 0.071329 | 2.61494 | 2.0186 | 0.047746 | 0.212062 | -4.27779 | 0 |
| PDE4A | 0.084479 | 1.949781 | 2.015762 | 0.04805 | 0.21282 | -4.28303 | 0 |
| BTBD6 | 0.09429 | 2.555385 | 2.004318 | 0.049294 | 0.215868 | -4.30405 | 0 |
| BOLA1 | 0.063906 | 2.249121 | 2.002873 | 0.049454 | 0.21624 | -4.3067 | 0 |
| GAR1 | 0.055623 | 2.193833 | 2.000339 | 0.049733 | 0.216922 | -4.31134 | 0 |
| HBD | -0.50294 | 1.792686 | -5.9681 | 1.17E-07 | 0.00012 | 7.444355 | 4 |
| IL1RAP | -0.33042 | 2.133162 | -5.89405 | 1.56E-07 | 0.000144 | 7.174655 | 1 |
| SLC7A5 | -0.37518 | 2.239037 | -5.63865 | 4.22E-07 | 0.00023 | 6.251248 | 1 |
| PPP4C | -0.28772 | 2.774022 | -5.20021 | 2.25E-06 | 0.000636 | 4.695885 | 1 |
| RALB | -0.19849 | 2.707276 | -5.18372 | 2.40E-06 | 0.000647 | 4.638223 | 1 |
| MVP | -0.2681 | 2.221548 | -5.14578 | 2.77E-06 | 0.000701 | 4.505868 | 1 |
| AQP9 | -0.30878 | 1.707655 | -5.13336 | 2.90E-06 | 0.000714 | 4.462617 | 4 |
| SERPINE2 | -0.41087 | 2.811387 | -5.07348 | 3.63E-06 | 0.000813 | 4.25469 | 0 |
| GADD45B | -0.7463 | 3.418643 | -5.04481 | 4.04E-06 | 0.000861 | 4.155455 | 1 |
| GABARAPL1 | -0.26834 | 3.422766 | -4.95718 | 5.59E-06 | 0.00103 | 3.853579 | 1 |
| SOCS3 | -0.42433 | 2.437404 | -4.87466 | 7.59E-06 | 0.001156 | 3.571287 | 1 |

| | | | | | | | |
|---|---|---|---|---|---|---|---|
| SPRY1 | -0.26192 | 2.93512 | -4.87167 | 7.67E-06 | 0.001156 | 3.561109 | 1 |
| MIDN | -0.30184 | 2.740416 | -4.8448 | 8.47E-06 | 0.001213 | 3.469645 | 1 |
| FBXW5 | -0.14529 | 3.057461 | -4.84141 | 8.58E-06 | 0.001213 | 3.458117 | 0 |
| IL4R | -0.27242 | 2.040523 | -4.79723 | 1.01E-05 | 0.001316 | 3.308274 | 1 |
| CSNK1D | -0.22476 | 3.076394 | -4.74972 | 1.20E-05 | 0.001448 | 3.1478 | 1 |
| CASP4 | -0.27505 | 2.63237 | -4.71413 | 1.37E-05 | 0.001592 | 3.028081 | 1 |
| PAG1 | -0.2801 | 2.331078 | -4.67907 | 1.55E-05 | 0.001695 | 2.910498 | 1 |
| GRB10 | -0.18873 | 2.397414 | -4.67338 | 1.58E-05 | 0.00172 | 2.891481 | 0 |
| ETS2 | -0.38989 | 3.333658 | -4.66223 | 1.65E-05 | 0.001758 | 2.85418 | 1 |
| CST7 | -0.27301 | 2.070358 | -4.65007 | 1.72E-05 | 0.001783 | 2.813581 | 4 |
| S100A12 | -0.56956 | 2.03036 | -4.64536 | 1.75E-05 | 0.001783 | 2.797871 | 4 |
| CEBPD | -0.34158 | 2.701308 | -4.63281 | 1.83E-05 | 0.001788 | 2.756019 | 1 |
| GRAMD1A | -0.26458 | 2.665041 | -4.61226 | 1.98E-05 | 0.001877 | 2.687618 | 1 |
| TAF10 | -0.15248 | 3.367935 | -4.60913 | 2.00E-05 | 0.001888 | 2.67722 | 1 |
| SLC25A37 | -0.16501 | 2.653493 | -4.60682 | 2.01E-05 | 0.001893 | 2.669545 | 4 |
| STK3 | -0.1253 | 2.220902 | -4.54402 | 2.52E-05 | 0.002206 | 2.461543 | 1 |
| CYP1B1 | -0.33175 | 2.790621 | -4.54168 | 2.54E-05 | 0.002212 | 2.453831 | 1 |
| XPO6 | -0.26203 | 2.787464 | -4.53149 | 2.64E-05 | 0.002245 | 2.420226 | 1 |
| TNIP2 | -0.21882 | 2.203882 | -4.52482 | 2.70E-05 | 0.002272 | 2.39822 | 1 |
| KLHL8 | -0.37598 | 2.673616 | -4.51891 | 2.76E-05 | 0.002287 | 2.378783 | 1 |
| UPP1 | -0.20431 | 2.523533 | -4.51874 | 2.76E-05 | 0.002287 | 2.378214 | 1 |
| JUNB | -0.44462 | 3.38529 | -4.49095 | 3.05E-05 | 0.002361 | 2.28686 | 1 |
| MMP8 | -0.31412 | 1.567875 | -4.41953 | 3.93E-05 | 0.002763 | 2.053364 | 4 |
| DOCK4 | -0.22007 | 2.415703 | -4.39296 | 4.32E-05 | 0.002914 | 1.966983 | 1 |
| SH3GLB2 | -0.14678 | 2.427343 | -4.36889 | 4.70E-05 | 0.003097 | 1.888947 | 2 |
| YIPF2 | -0.16335 | 2.466927 | -4.35604 | 4.92E-05 | 0.003184 | 1.847394 | 3 |
| ATP13A3 | -0.46471 | 3.015162 | -4.35086 | 5.01E-05 | 0.003218 | 1.830654 | 1 |
| SPTLC1 | -0.18107 | 2.457065 | -4.33618 | 5.27E-05 | 0.003364 | 1.783298 | 1 |
| VPS54 | -0.17111 | 2.392884 | -4.29955 | 5.99E-05 | 0.003663 | 1.665447 | 1 |
| LCN2 | -0.28394 | 1.936728 | -4.28252 | 6.36E-05 | 0.003808 | 1.61084 | 4 |
| ARL8A | -0.29978 | 2.764815 | -4.27868 | 6.44E-05 | 0.00382 | 1.59853 | 1 |
| ALPL | -0.27582 | 2.225322 | -4.25932 | 6.89E-05 | 0.003936 | 1.53661 | 1 |
| UBALD2 | -0.20701 | 3.114263 | -4.25862 | 6.91E-05 | 0.003936 | 1.534382 | 1 |
| PHC2 | -0.22369 | 2.565357 | -4.24952 | 7.13E-05 | 0.00401 | 1.505314 | 1 |
| B4GALT5 | -0.34862 | 3.072497 | -4.24483 | 7.25E-05 | 0.004015 | 1.490359 | 1 |
| HDAC7 | -0.16404 | 2.520718 | -4.21446 | 8.05E-05 | 0.004232 | 1.393716 | 0 |
| THBS1 | -0.64267 | 3.728622 | -4.20184 | 8.41E-05 | 0.004378 | 1.353691 | 1 |
| NPLOC4 | -0.24117 | 2.884651 | -4.18547 | 8.90E-05 | 0.004435 | 1.30184 | 1 |
| PLSCR1 | -0.30249 | 2.448205 | -4.17889 | 9.11E-05 | 0.004511 | 1.28102 | 1 |
| POR | -0.27244 | 2.67387 | -4.16041 | 9.70E-05 | 0.004714 | 1.222664 | 1 |
| NUCB1 | -0.20046 | 3.24272 | -4.14552 | 0.000102 | 0.00488 | 1.175752 | 1 |
| GALNT2 | -0.14769 | 2.359568 | -4.13985 | 0.000104 | 0.004948 | 1.157917 | 1 |
| UGCG | -0.312 | 2.450743 | -4.1196 | 0.000112 | 0.00511 | 1.094299 | 1 |
| AGTRAP | -0.20529 | 2.397509 | -4.09405 | 0.000122 | 0.00542 | 1.014318 | 1 |
| STOM | -0.2426 | 3.217438 | -4.08848 | 0.000124 | 0.005496 | 0.996908 | 1 |
| KLHL2 | -0.16677 | 2.094675 | -4.07789 | 0.000129 | 0.005626 | 0.963852 | 1 |
| LDLRAD3 | -0.16847 | 2.433435 | -4.07301 | 0.000131 | 0.005664 | 0.948634 | 1 |
| GLI3 | -0.15557 | 2.118289 | -4.06373 | 0.000135 | 0.005763 | 0.91974 | 0 |
| PPBP | -0.33384 | 1.787833 | -4.05695 | 0.000138 | 0.005783 | 0.898648 | 4 |
| A4GALT | -0.18809 | 2.548098 | -4.04906 | 0.000142 | 0.005879 | 0.874116 | 3 |
| HRH2 | -0.40465 | 2.467588 | -4.04522 | 0.000144 | 0.005892 | 0.862193 | 1 |

| | | | | | | | |
|---|---|---|---|---|---|---|---|
| FSTL3 | -0.27029 | 2.857245 | -4.03661 | 0.000148 | 0.005965 | 0.835493 | 3 |
| LIMK2 | -0.19553 | 2.004863 | -4.03601 | 0.000148 | 0.005965 | 0.833615 | 1 |
| IFITM1 | -0.18649 | 3.417857 | -4.03156 | 0.000151 | 0.006014 | 0.819822 | 1 |
| TNFRSF1A | -0.24134 | 3.139812 | -3.99465 | 0.000171 | 0.006472 | 0.705776 | 1 |
| GRK6 | -0.12457 | 2.218044 | -3.97571 | 0.000182 | 0.006723 | 0.647483 | 0 |
| RRBP1 | -0.16085 | 2.651379 | -3.9676 | 0.000187 | 0.006877 | 0.622569 | 1 |
| KDM5B | -0.16012 | 2.494566 | -3.96511 | 0.000188 | 0.006908 | 0.614923 | 0 |
| SELL | -0.27692 | 1.790401 | -3.95323 | 0.000196 | 0.007115 | 0.578484 | 4 |
| STX11 | -0.30568 | 2.311642 | -3.93312 | 0.00021 | 0.007471 | 0.51698 | 1 |
| ABLIM2 | -0.20151 | 2.102601 | -3.90948 | 0.000227 | 0.007864 | 0.444901 | 2 |
| IER3 | -0.36863 | 3.073869 | -3.89985 | 0.000234 | 0.008021 | 0.415634 | 0 |
| HES1 | -0.28759 | 2.147091 | -3.89475 | 0.000238 | 0.008095 | 0.400132 | 1 |
| ADAM19 | -0.35531 | 2.729655 | -3.89306 | 0.00024 | 0.008109 | 0.394984 | 1 |
| SLC6A6 | -0.37802 | 2.871966 | -3.89214 | 0.00024 | 0.008118 | 0.392204 | 3 |
| GCA | -0.20037 | 2.19732 | -3.88708 | 0.000245 | 0.008193 | 0.376851 | 4 |
| SAMD8 | -0.26127 | 2.447909 | -3.87972 | 0.000251 | 0.008295 | 0.354549 | 1 |
| FNDC3A | -0.27052 | 2.887441 | -3.87871 | 0.000251 | 0.008295 | 0.35147 | 1 |
| MAPKAPK2 | -0.28353 | 3.45187 | -3.8716 | 0.000257 | 0.008431 | 0.329962 | 1 |
| RAB35 | -0.21843 | 2.345221 | -3.85613 | 0.000271 | 0.008648 | 0.28321 | 1 |
| PTK2B | -0.1675 | 1.998052 | -3.8357 | 0.00029 | 0.009006 | 0.22163 | 4 |
| SEMA4B | -0.25949 | 2.448987 | -3.81678 | 0.000309 | 0.009352 | 0.164799 | 1 |
| CHD7 | -0.1881 | 2.396377 | -3.81437 | 0.000311 | 0.00941 | 0.15758 | 0 |
| KCTD2 | -0.1531 | 2.724039 | -3.80137 | 0.000325 | 0.009688 | 0.118615 | 0 |
| ETV6 | -0.2633 | 2.421428 | -3.78131 | 0.000347 | 0.010198 | 0.058693 | 1 |
| VCAN | -0.33114 | 2.887633 | -3.76891 | 0.000361 | 0.010437 | 0.021746 | 1 |
| PROK2 | -0.27433 | 1.70814 | -3.76637 | 0.000364 | 0.010489 | 0.014186 | 3 |
| RAB31 | -0.31605 | 2.368099 | -3.76432 | 0.000367 | 0.010525 | 0.008094 | 1 |
| GNB2 | -0.12699 | 2.815318 | -3.76098 | 0.000371 | 0.010599 | -0.00183 | 1 |
| AP3S1 | -0.14085 | 2.555764 | -3.76016 | 0.000372 | 0.010599 | -0.00428 | 1 |
| PGS1 | -0.15553 | 2.18915 | -3.73781 | 0.0004 | 0.011006 | -0.07061 | 1 |
| R3HDM4 | -0.1738 | 2.164678 | -3.73535 | 0.000403 | 0.011035 | -0.07786 | 1 |
| RSBN1 | -0.13455 | 2.397324 | -3.73416 | 0.000404 | 0.01105 | -0.08141 | 1 |
| PTX3 | -0.51457 | 2.463567 | -3.72707 | 0.000414 | 0.011221 | -0.10238 | 1 |
| SRPK1 | -0.13009 | 2.391413 | -3.72468 | 0.000417 | 0.01125 | -0.10943 | 1 |
| ACVR1B | -0.1698 | 2.221762 | -3.7186 | 0.000425 | 0.011419 | -0.12738 | 1 |
| ARL4A | -0.21681 | 2.369269 | -3.70254 | 0.000448 | 0.011767 | -0.17473 | 1 |
| ATN1 | -0.1457 | 2.879938 | -3.68313 | 0.000477 | 0.012271 | -0.23178 | 1 |
| EIF4G3 | -0.13813 | 2.860645 | -3.67891 | 0.000484 | 0.012322 | -0.24417 | 0 |
| BAZ1A | -0.20842 | 2.411053 | -3.67567 | 0.000489 | 0.012424 | -0.25365 | 1 |
| BEX1 | -0.34812 | 2.14562 | -3.66345 | 0.000509 | 0.012757 | -0.28944 | 0 |
| EFEMP2 | -0.12655 | 2.895192 | -3.6549 | 0.000523 | 0.013019 | -0.3144 | 0 |
| SH3GL1 | -0.14941 | 2.276669 | -3.65111 | 0.000529 | 0.013123 | -0.32547 | 1 |
| FLOT2 | -0.30223 | 2.944213 | -3.64969 | 0.000532 | 0.013146 | -0.32963 | 1 |
| TGM2 | -0.34802 | 3.733888 | -3.62322 | 0.000579 | 0.013879 | -0.40665 | 1 |
| MAD2L2 | -0.12455 | 2.566147 | -3.62124 | 0.000582 | 0.013929 | -0.41239 | 3 |
| IL1R1 | -0.1688 | 2.741799 | -3.61693 | 0.00059 | 0.013988 | -0.42491 | 1 |
| ICA1 | -0.24409 | 2.713267 | -3.6157 | 0.000593 | 0.014025 | -0.42849 | 1 |
| KCNJ15 | -0.15723 | 1.557773 | -3.61376 | 0.000596 | 0.014093 | -0.4341 | 4 |
| RHOG | -0.12682 | 3.055468 | -3.61232 | 0.000599 | 0.014104 | -0.43828 | 1 |
| CSF3R | -0.25517 | 2.014896 | -3.60707 | 0.000609 | 0.014262 | -0.45348 | 4 |
| INSR | -0.29282 | 2.936602 | -3.60405 | 0.000615 | 0.014343 | -0.46222 | 1 |

| | | | | | | | |
|---|---|---|---|---|---|---|---|
| RNF24 | -0.14152 | 2.315856 | -3.60336 | 0.000617 | 0.014356 | -0.46423 | 1 |
| S100A11 | -0.20718 | 3.581594 | -3.58354 | 0.000657 | 0.014973 | -0.52147 | 1 |
| GNA15 | -0.16204 | 1.883966 | -3.57901 | 0.000666 | 0.015072 | -0.53452 | 1 |
| RPL22L1 | -0.20125 | 2.453104 | -3.57371 | 0.000678 | 0.01523 | -0.54979 | 1 |
| ARID5A | -0.15344 | 2.167391 | -3.55485 | 0.000719 | 0.015821 | -0.60397 | 0 |
| SERPINA1 | -0.25812 | 2.412322 | -3.54321 | 0.000746 | 0.016193 | -0.63733 | 4 |
| FLOT1 | -0.22688 | 3.318631 | -3.52381 | 0.000794 | 0.016854 | -0.69274 | 1 |
| AGO2 | -0.19422 | 2.944095 | -3.52368 | 0.000794 | 0.016854 | -0.69312 | 1 |
| YWHAH | -0.09711 | 3.110526 | -3.52183 | 0.000799 | 0.016904 | -0.69839 | 1 |
| SLC38A5 | -0.12155 | 1.795539 | -3.51943 | 0.000805 | 0.016991 | -0.70522 | 3 |
| OSBPL6 | -0.23225 | 1.934656 | -3.50278 | 0.000848 | 0.017587 | -0.75259 | 1 |
| RAB13 | -0.28142 | 3.01044 | -3.49369 | 0.000872 | 0.017901 | -0.77838 | 1 |
| PYGL | -0.24267 | 2.35682 | -3.48931 | 0.000884 | 0.018075 | -0.79082 | 1 |
| ASAP1 | -0.21947 | 3.002237 | -3.4863 | 0.000893 | 0.01816 | -0.79934 | 1 |
| TRAF7 | -0.14965 | 2.326508 | -3.48538 | 0.000895 | 0.018186 | -0.80194 | 1 |
| CUL4B | -0.12289 | 2.487202 | -3.48023 | 0.00091 | 0.018332 | -0.8165 | 1 |
| MAPK3 | -0.11003 | 3.126138 | -3.47619 | 0.000922 | 0.018417 | -0.82792 | 1 |
| RBMS1 | -0.18512 | 3.118925 | -3.46988 | 0.00094 | 0.018677 | -0.84577 | 1 |
| RAB20 | -0.17922 | 2.58752 | -3.45957 | 0.000971 | 0.01905 | -0.87482 | 1 |
| CA1 | -0.24537 | 1.711187 | -3.43754 | 0.00104 | 0.019847 | -0.93675 | 4 |
| RHOBTB2 | -0.2332 | 2.539234 | -3.43154 | 0.001059 | 0.020132 | -0.95358 | 1 |
| PNP | -0.36097 | 2.614119 | -3.42873 | 0.001069 | 0.020243 | -0.96144 | 1 |
| PRR13 | -0.11629 | 3.067891 | -3.42259 | 0.001089 | 0.020433 | -0.97863 | 1 |
| SERINC2 | -0.18659 | 1.947987 | -3.41818 | 0.001104 | 0.020582 | -0.99095 | 3 |
| ARG1 | -0.21362 | 1.658105 | -3.41577 | 0.001112 | 0.020648 | -0.9977 | 4 |
| ITPRIP | -0.24368 | 2.527537 | -3.41468 | 0.001116 | 0.020652 | -1.00074 | 1 |
| AVL9 | -0.10018 | 2.170213 | -3.40862 | 0.001137 | 0.020933 | -1.01766 | 1 |
| SLC50A1 | -0.17914 | 2.603233 | -3.39621 | 0.001182 | 0.021547 | -1.05223 | 1 |
| SERPINE1 | -0.69325 | 3.18087 | -3.39372 | 0.001191 | 0.021659 | -1.05913 | 1 |
| STAT5B | -0.17538 | 2.65557 | -3.39351 | 0.001192 | 0.021659 | -1.05972 | 1 |
| EFNA1 | -0.28121 | 2.703115 | -3.3899 | 0.001205 | 0.021822 | -1.06975 | 1 |
| CD55 | -0.24789 | 3.213753 | -3.38402 | 0.001227 | 0.022096 | -1.0861 | 1 |
| UIMC1 | -0.13894 | 2.201222 | -3.38236 | 0.001233 | 0.022173 | -1.0907 | 1 |
| SIRPA | -0.13679 | 2.894122 | -3.36725 | 0.001292 | 0.022726 | -1.13255 | 1 |
| C9orf16 | -0.11794 | 2.556551 | -3.36695 | 0.001293 | 0.022726 | -1.13339 | 2 |
| IL1RL1 | -0.47484 | 2.337756 | -3.36456 | 0.001303 | 0.022812 | -1.13998 | 1 |
| SNX4 | -0.21679 | 2.192441 | -3.36222 | 0.001312 | 0.022852 | -1.14647 | 1 |
| SLC16A5 | -0.11976 | 2.490199 | -3.36158 | 0.001315 | 0.022874 | -1.14824 | 0 |
| TRMT6 | -0.11712 | 2.139283 | -3.35346 | 0.001348 | 0.023298 | -1.17063 | 3 |
| PKNOX1 | -0.1793 | 2.197263 | -3.35084 | 0.001359 | 0.023415 | -1.17787 | 1 |
| SERPINB1 | -0.33929 | 2.811865 | -3.34128 | 0.001399 | 0.023821 | -1.20419 | 1 |
| STAT3 | -0.33768 | 3.688595 | -3.33687 | 0.001418 | 0.024074 | -1.21632 | 1 |
| EFHD2 | -0.17563 | 2.225087 | -3.33406 | 0.00143 | 0.024173 | -1.22406 | 1 |
| TRPS1 | -0.14701 | 2.236019 | -3.32592 | 0.001466 | 0.02463 | -1.24639 | 0 |
| CACNB1 | -0.14515 | 1.981625 | -3.32552 | 0.001468 | 0.02463 | -1.24748 | 2 |
| TBC1D22B | -0.15234 | 1.997195 | -3.32449 | 0.001473 | 0.02463 | -1.25032 | 1 |
| ITGB5 | -0.12553 | 2.549886 | -3.32416 | 0.001474 | 0.02463 | -1.25121 | 1 |
| SNX11 | -0.1222 | 1.806282 | -3.31443 | 0.001519 | 0.025146 | -1.27788 | 1 |
| TWF1 | -0.12863 | 2.62142 | -3.30889 | 0.001544 | 0.025405 | -1.29302 | 1 |
| METTL22 | -0.14983 | 2.378703 | -3.30022 | 0.001586 | 0.025721 | -1.31671 | 1 |
| TNIP1 | -0.22817 | 2.883648 | -3.29632 | 0.001605 | 0.025856 | -1.32732 | 1 |

| | | | | | | | |
|---|---|---|---|---|---|---|---|
| USP3 | -0.16491 | 2.614462 | -3.29533 | 0.001609 | 0.025856 | -1.33003 | 1 |
| SPRY4 | -0.17902 | 2.761838 | -3.29515 | 0.00161 | 0.025856 | -1.33051 | 0 |
| MCTP2 | -0.21192 | 1.579762 | -3.29065 | 0.001632 | 0.026115 | -1.34278 | 3 |
| NR1H2 | -0.14061 | 2.388849 | -3.27786 | 0.001697 | 0.026691 | -1.37755 | 1 |
| IL18RAP | -0.1659 | 1.429267 | -3.27411 | 0.001716 | 0.026888 | -1.38774 | 4 |
| ALOX5 | -0.16644 | 2.094121 | -3.2623 | 0.001779 | 0.027526 | -1.41974 | 4 |
| PLOD2 | -0.31123 | 2.571618 | -3.26192 | 0.001781 | 0.027529 | -1.42074 | 1 |
| PHF5A | -0.23586 | 2.044126 | -3.25601 | 0.001813 | 0.027925 | -1.43673 | 1 |
| TMEM54 | -0.14338 | 2.321347 | -3.24996 | 0.001846 | 0.028339 | -1.45308 | 2 |
| NME4 | -0.10286 | 2.981185 | -3.24952 | 0.001848 | 0.028351 | -1.45425 | 0 |
| MSMO1 | -0.16938 | 2.098805 | -3.22626 | 0.001982 | 0.029725 | -1.51687 | 3 |
| ALPK1 | -0.15928 | 1.668595 | -3.22406 | 0.001995 | 0.029832 | -1.52279 | 3 |
| VAMP7 | -0.16758 | 2.674473 | -3.22008 | 0.002019 | 0.03014 | -1.53347 | 1 |
| OAT | -0.12362 | 2.506069 | -3.21708 | 0.002037 | 0.03032 | -1.54149 | 0 |
| SEC24A | -0.2177 | 2.519008 | -3.20856 | 0.00209 | 0.030919 | -1.56432 | 1 |
| SP100 | -0.17673 | 2.577122 | -3.2023 | 0.002129 | 0.031336 | -1.58105 | 1 |
| HS2ST1 | -0.07649 | 2.626605 | -3.19656 | 0.002166 | 0.031672 | -1.59636 | 0 |
| ZYX | -0.12081 | 2.871018 | -3.18922 | 0.002214 | 0.032183 | -1.61594 | 3 |
| TMEM165 | -0.18918 | 2.888998 | -3.18559 | 0.002238 | 0.03239 | -1.6256 | 1 |
| CD44 | -0.18226 | 2.709187 | -3.16528 | 0.002377 | 0.033723 | -1.6795 | 1 |
| SLC38A10 | -0.09206 | 2.388514 | -3.15984 | 0.002416 | 0.03415 | -1.69392 | 0 |
| PROS1 | -0.1999 | 2.835143 | -3.15958 | 0.002418 | 0.03415 | -1.69459 | 0 |
| STX5 | -0.15639 | 2.241074 | -3.1592 | 0.002421 | 0.034158 | -1.69562 | 1 |
| OLFM4 | -0.22781 | 1.710046 | -3.15792 | 0.00243 | 0.034158 | -1.69899 | 0 |
| SBNO2 | -0.13222 | 2.155648 | -3.15546 | 0.002447 | 0.034292 | -1.7055 | 1 |
| FPR2 | -0.12846 | 1.624105 | -3.15277 | 0.002467 | 0.034443 | -1.7126 | 4 |
| UHRF1BP1L | -0.14243 | 2.449939 | -3.14611 | 0.002516 | 0.034925 | -1.73018 | 1 |
| GLT1D1 | -0.11381 | 1.522555 | -3.13736 | 0.002582 | 0.035499 | -1.75324 | 2 |
| KIAA1958 | -0.10305 | 1.875389 | -3.10317 | 0.002855 | 0.03779 | -1.84288 | 0 |
| MGAT4B | -0.09446 | 2.67546 | -3.10143 | 0.002869 | 0.037864 | -1.84742 | 0 |
| JAK3 | -0.11039 | 1.80256 | -3.10109 | 0.002872 | 0.037864 | -1.84831 | 3 |
| RNF10 | -0.25343 | 3.335194 | -3.08893 | 0.002976 | 0.038697 | -1.88001 | 1 |
| NRSN2 | -0.10977 | 2.52969 | -3.08673 | 0.002996 | 0.038829 | -1.88573 | 1 |
| PXK | -0.13751 | 2.551427 | -3.08518 | 0.003009 | 0.038947 | -1.88975 | 1 |
| GNB1 | -0.11356 | 3.274691 | -3.08319 | 0.003027 | 0.039087 | -1.89492 | 1 |
| ALG13 | -0.11298 | 2.371443 | -3.06272 | 0.003213 | 0.040707 | -1.94801 | 0 |
| HDGF | -0.10151 | 2.884598 | -3.06066 | 0.003232 | 0.040921 | -1.95333 | 1 |
| SLC25A28 | -0.10157 | 2.636423 | -3.05736 | 0.003263 | 0.041196 | -1.96185 | 1 |
| RHBDD2 | -0.09046 | 2.898115 | -3.0525 | 0.003309 | 0.041423 | -1.97441 | 1 |
| TCEA2 | -0.09478 | 2.223919 | -3.0507 | 0.003327 | 0.041524 | -1.97905 | 0 |
| FNDC3B | -0.24339 | 3.061139 | -3.04958 | 0.003337 | 0.041626 | -1.98194 | 0 |
| ATP9A | -0.14565 | 2.503417 | -3.03888 | 0.003443 | 0.042482 | -2.00949 | 0 |
| ELL2 | -0.3399 | 3.127246 | -3.03533 | 0.003478 | 0.042769 | -2.01861 | 1 |
| MVD | -0.1214 | 2.18444 | -3.02934 | 0.003539 | 0.043333 | -2.03401 | 2 |
| C3orf52 | -0.25919 | 2.160865 | -3.02814 | 0.003551 | 0.043399 | -2.03707 | 3 |
| QPCT | -0.17485 | 1.965592 | -3.01953 | 0.00364 | 0.044043 | -2.05913 | 0 |
| LDLR | -0.37263 | 2.774008 | -3.01936 | 0.003642 | 0.044043 | -2.05957 | 1 |
| TSPYL1 | -0.11417 | 3.102961 | -3.00606 | 0.003784 | 0.045168 | -2.09355 | 0 |
| NAV2 | -0.27507 | 2.835567 | -3.00302 | 0.003818 | 0.04541 | -2.10132 | 1 |
| CLEC2B | -0.21564 | 2.209299 | -2.9991 | 0.003861 | 0.045595 | -2.1113 | 1 |
| ADIPOR2 | -0.11604 | 2.87179 | -2.99806 | 0.003872 | 0.045627 | -2.11395 | 1 |

| | | | | | | | |
|---|---|---|---|---|---|---|---|
| DEFA4 | -0.28403 | 1.70487 | -2.98992 | 0.003964 | 0.046268 | -2.13465 | 0 |
| TIMP1 | -0.32268 | 3.739817 | -2.98808 | 0.003985 | 0.046389 | -2.13934 | 1 |
| NUP98 | -0.21104 | 2.819705 | -2.98358 | 0.004036 | 0.046814 | -2.15076 | 1 |
| SLC11A1 | -0.20269 | 2.223391 | -2.97935 | 0.004085 | 0.047154 | -2.16148 | 4 |
| TINF2 | -0.13359 | 2.585733 | -2.97828 | 0.004098 | 0.047199 | -2.16419 | 1 |
| CLU | -0.09386 | 2.979779 | -2.97777 | 0.004104 | 0.047212 | -2.16549 | 0 |
| GLRX | -0.17891 | 3.100424 | -2.97376 | 0.004151 | 0.047568 | -2.17564 | 1 |
| SLA | -0.15237 | 1.888675 | -2.95454 | 0.004385 | 0.049088 | -2.22416 | 4 |
| GLIS2 | -0.11647 | 2.459217 | -2.95236 | 0.004412 | 0.049175 | -2.22966 | 0 |
| BCL2A1 | -0.18013 | 1.721271 | -2.95235 | 0.004412 | 0.049175 | -2.22968 | 4 |
| BASP1 | -0.1394 | 2.489432 | -2.95205 | 0.004416 | 0.049185 | -2.23043 | 4 |
| KLF16 | -0.14952 | 2.240672 | -2.94845 | 0.004462 | 0.049463 | -2.23949 | 2 |
| TRABD | -0.09856 | 2.537598 | -2.94806 | 0.004467 | 0.049463 | -2.24048 | 2 |
| SLCO5A1 | -0.21595 | 2.437017 | -2.94722 | 0.004477 | 0.049518 | -2.24258 | 1 |
| STARD3NL | -0.09032 | 2.388723 | -2.94481 | 0.004508 | 0.049763 | -2.24863 | 3 |
| IL1R2 | -0.26115 | 2.320314 | -2.94362 | 0.004523 | 0.049895 | -2.25163 | 4 |
| NOL6 | -0.12664 | 2.175444 | -2.9357 | 0.004626 | 0.050586 | -2.27149 | 1 |
| FERMT3 | -0.09653 | 2.043035 | -2.93372 | 0.004652 | 0.050807 | -2.27646 | 3 |
| EFCAB12 | -0.11925 | 1.581283 | -2.93179 | 0.004677 | 0.050894 | -2.2813 | 2 |
| FOSL2 | -0.24043 | 3.037159 | -2.93158 | 0.00468 | 0.050894 | -2.28182 | 1 |
| TBC1D15 | -0.15068 | 2.683774 | -2.92945 | 0.004708 | 0.051075 | -2.28715 | 1 |
| TRIB1 | -0.22022 | 2.694517 | -2.92828 | 0.004724 | 0.051213 | -2.29009 | 1 |
| SLC39A1 | -0.0801 | 2.358851 | -2.92507 | 0.004767 | 0.051552 | -2.29811 | 3 |
| YIPF1 | -0.19043 | 2.241077 | -2.92166 | 0.004813 | 0.051826 | -2.30663 | 1 |
| DPY19L3 | -0.09363 | 1.624204 | -2.91856 | 0.004856 | 0.052217 | -2.31436 | 0 |
| RALGAPA2 | -0.20313 | 2.563796 | -2.91011 | 0.004973 | 0.052795 | -2.33542 | 1 |
| WDR45 | -0.09154 | 2.913611 | -2.90934 | 0.004984 | 0.052846 | -2.33734 | 1 |
| AGFG1 | -0.17483 | 3.124887 | -2.90806 | 0.005002 | 0.052973 | -2.34053 | 1 |
| MCTP1 | -0.10898 | 2.101844 | -2.90709 | 0.005015 | 0.053051 | -2.34296 | 3 |
| B3GNT5 | -0.18238 | 2.148566 | -2.90563 | 0.005036 | 0.053207 | -2.34657 | 0 |
| HK1 | -0.13314 | 2.946388 | -2.8972 | 0.005157 | 0.053867 | -2.36753 | 1 |
| MTMR3 | -0.13696 | 2.61453 | -2.89695 | 0.00516 | 0.053872 | -2.36815 | 0 |
| CFB | -0.29213 | 2.352587 | -2.89006 | 0.005261 | 0.054502 | -2.38523 | 1 |
| ALAS2 | -0.15097 | 1.685704 | -2.86405 | 0.005659 | 0.057493 | -2.44943 | 4 |
| HHEX | -0.13057 | 2.016617 | -2.85693 | 0.005772 | 0.058374 | -2.46693 | 2 |
| GTPBP2 | -0.09513 | 2.40523 | -2.85473 | 0.005808 | 0.058584 | -2.47234 | 1 |
| ARID3A | -0.10406 | 1.917863 | -2.84911 | 0.005899 | 0.058993 | -2.48611 | 0 |
| E2F3 | -0.10133 | 2.190023 | -2.84846 | 0.00591 | 0.059016 | -2.48771 | 3 |
| TCTEX1D1 | -0.13484 | 1.557602 | -2.84652 | 0.005942 | 0.059234 | -2.49246 | 0 |
| OXSR1 | -0.12632 | 2.594556 | -2.84435 | 0.005978 | 0.059491 | -2.49778 | 1 |
| RPS19BP1 | -0.0919 | 2.840835 | -2.84344 | 0.005993 | 0.059558 | -2.50001 | 1 |
| ENO1 | -0.21997 | 3.304948 | -2.84117 | 0.006031 | 0.059788 | -2.50556 | 1 |
| GCLM | -0.22433 | 2.00965 | -2.84011 | 0.006048 | 0.059854 | -2.50814 | 1 |
| LINC0026C | -0.35888 | 2.350014 | -2.83696 | 0.006102 | 0.060177 | -2.51585 | 1 |
| PARD3 | -0.18711 | 2.723741 | -2.82855 | 0.006246 | 0.061316 | -2.53638 | 1 |
| CAMKK2 | -0.09383 | 2.306554 | -2.8242 | 0.006321 | 0.061748 | -2.54696 | 0 |
| RAB1B | -0.15029 | 3.462807 | -2.82033 | 0.006389 | 0.061931 | -2.55637 | 1 |
| ATP11A | -0.2751 | 3.064172 | -2.81857 | 0.006421 | 0.062119 | -2.56066 | 1 |
| BMX | -0.13859 | 1.787435 | -2.81729 | 0.006443 | 0.06228 | -2.56375 | 2 |
| SGMS2 | -0.12347 | 2.832353 | -2.81673 | 0.006453 | 0.062307 | -2.56511 | 1 |
| IFITM2 | -0.1464 | 3.65094 | -2.79979 | 0.006762 | 0.064516 | -2.60618 | 1 |

| | | | | | | | |
|---|---|---|---|---|---|---|---|
| MMP14 | -0.13469 | 1.952085 | -2.79973 | 0.006763 | 0.064516 | -2.60633 | 1 |
| KPNA1 | -0.16493 | 2.929299 | -2.79626 | 0.006828 | 0.064925 | -2.6147 | 1 |
| RASA2 | -0.13772 | 1.936424 | -2.79265 | 0.006896 | 0.065073 | -2.62341 | 0 |
| SPTB | -0.21479 | 2.775382 | -2.78461 | 0.00705 | 0.066276 | -2.64281 | 1 |
| TTC7B | -0.14396 | 2.654109 | -2.77458 | 0.007246 | 0.067538 | -2.66691 | 0 |
| ST6GALNAC | -0.13964 | 2.096388 | -2.77065 | 0.007325 | 0.067925 | -2.67636 | 1 |
| SAT1 | -0.3474 | 3.381301 | -2.76592 | 0.00742 | 0.06843 | -2.68768 | 1 |
| COPE | -0.11744 | 2.645845 | -2.75695 | 0.007604 | 0.069611 | -2.70914 | 1 |
| MTF1 | -0.15431 | 2.568991 | -2.75351 | 0.007675 | 0.070084 | -2.71736 | 1 |
| FAM114A1 | -0.10022 | 2.504942 | -2.75268 | 0.007693 | 0.070207 | -2.71935 | 0 |
| TCTA | -0.08249 | 2.686659 | -2.75212 | 0.007704 | 0.070272 | -2.72068 | 0 |
| GDI1 | -0.12665 | 3.189655 | -2.74394 | 0.007878 | 0.071337 | -2.74017 | 1 |
| LRP10 | -0.15916 | 2.805941 | -2.73964 | 0.00797 | 0.071951 | -2.75039 | 1 |
| MS4A3 | -0.12816 | 1.37583 | -2.73921 | 0.00798 | 0.071961 | -2.75142 | 3 |
| IFNGR2 | -0.12634 | 2.806742 | -2.73766 | 0.008013 | 0.072144 | -2.75511 | 1 |
| CLSTN3 | -0.12744 | 1.979529 | -2.73593 | 0.008051 | 0.07234 | -2.7592 | 2 |
| DDX60L | -0.12998 | 2.163699 | -2.7331 | 0.008113 | 0.072692 | -2.76592 | 1 |
| GNAI3 | -0.17116 | 2.643588 | -2.72964 | 0.00819 | 0.073174 | -2.77413 | 1 |
| GADD45G | -0.30036 | 2.469902 | -2.72531 | 0.008286 | 0.073887 | -2.7844 | 0 |
| STRN4 | -0.11603 | 2.313653 | -2.72214 | 0.008358 | 0.074296 | -2.7919 | 1 |
| KLF5 | -0.1343 | 1.957846 | -2.71837 | 0.008443 | 0.074573 | -2.80081 | 3 |
| AFTPH | -0.14966 | 2.490038 | -2.71814 | 0.008448 | 0.074573 | -2.80136 | 0 |
| LILRA5 | -0.12312 | 1.837755 | -2.71702 | 0.008474 | 0.074723 | -2.80399 | 4 |
| PRICKLE3 | -0.08389 | 2.150007 | -2.71332 | 0.008559 | 0.075245 | -2.81273 | 0 |
| CD177 | -0.17669 | 2.028844 | -2.71183 | 0.008594 | 0.075435 | -2.81625 | 4 |
| PLEKHO2 | -0.11885 | 2.112463 | -2.70793 | 0.008684 | 0.075838 | -2.82543 | 3 |
| COL18A1 | -0.10692 | 2.318904 | -2.70393 | 0.008779 | 0.076143 | -2.83487 | 2 |
| SGTB | -0.11915 | 2.148921 | -2.69192 | 0.009067 | 0.077678 | -2.86307 | 0 |
| CCNH | -0.18415 | 2.57417 | -2.68672 | 0.009194 | 0.078157 | -2.87525 | 1 |
| VNN2 | -0.24483 | 1.667016 | -2.68485 | 0.009241 | 0.078475 | -2.87964 | 4 |
| CYB5R3 | -0.11977 | 3.63149 | -2.68262 | 0.009296 | 0.078677 | -2.88486 | 0 |
| SLC1A5 | -0.13525 | 2.129406 | -2.68244 | 0.0093 | 0.078677 | -2.88527 | 3 |
| STK16 | -0.08637 | 1.93024 | -2.66526 | 0.009738 | 0.081338 | -2.92533 | 0 |
| UBE2J1 | -0.17961 | 2.78879 | -2.66244 | 0.009811 | 0.081657 | -2.9319 | 1 |
| FTH1 | -0.12409 | 3.764435 | -2.6584 | 0.009918 | 0.082272 | -2.94129 | 1 |
| SNORA71C | -0.15253 | 2.36666 | -2.65499 | 0.010008 | 0.08271 | -2.9492 | 0 |
| NFE2 | -0.12221 | 1.620088 | -2.65359 | 0.010045 | 0.082934 | -2.95244 | 4 |
| TGFB1 | -0.13045 | 1.964813 | -2.64273 | 0.01034 | 0.084367 | -2.97758 | 0 |
| CCP110 | -0.09443 | 1.904307 | -2.63811 | 0.010467 | 0.085166 | -2.98826 | 0 |
| DNTTIP1 | -0.09288 | 2.374776 | -2.63654 | 0.010511 | 0.08518 | -2.99187 | 0 |
| SPHK1 | -0.13215 | 2.012731 | -2.63634 | 0.010517 | 0.08518 | -2.99235 | 3 |
| CHIC2 | -0.12109 | 2.590846 | -2.6312 | 0.010661 | 0.085977 | -3.00418 | 1 |
| PDE4D | -0.10965 | 2.375454 | -2.627 | 0.01078 | 0.086623 | -3.01385 | 1 |
| CEBPB | -0.12348 | 2.256793 | -2.62676 | 0.010787 | 0.086623 | -3.0144 | 2 |
| TFE3 | -0.13472 | 1.98933 | -2.62553 | 0.010822 | 0.086763 | -3.01723 | 1 |
| BCL6 | -0.26495 | 3.115334 | -2.62464 | 0.010847 | 0.08681 | -3.01929 | 1 |
| CHSY1 | -0.12054 | 2.252026 | -2.62246 | 0.01091 | 0.087111 | -3.02429 | 1 |
| ALG2 | -0.10334 | 2.361569 | -2.62106 | 0.010951 | 0.087315 | -3.02751 | 1 |
| S100P | -0.1001 | 1.787591 | -2.61772 | 0.011048 | 0.087769 | -3.03518 | 4 |
| C1RL | -0.10149 | 2.000282 | -2.61659 | 0.011081 | 0.087911 | -3.03777 | 1 |
| PLAC8 | -0.12987 | 1.53871 | -2.61576 | 0.011105 | 0.088024 | -3.03967 | 4 |

| | | | | | | | |
|---|---|---|---|---|---|---|---|
| PDLIM7 | -0.14025 | 2.400564 | -2.61372 | 0.011165 | 0.088219 | -3.04435 | 0 |
| ZFAS1 | -0.2453 | 2.896532 | -2.60787 | 0.011338 | 0.089186 | -3.05773 | 1 |
| TXNDC9 | -0.08151 | 2.551151 | -2.6061 | 0.011391 | 0.089493 | -3.06177 | 1 |
| EGFL7 | -0.10223 | 2.35351 | -2.60496 | 0.011425 | 0.089608 | -3.06438 | 2 |
| P2RX1 | -0.11623 | 1.826934 | -2.60031 | 0.011566 | 0.090077 | -3.075 | 3 |
| NAMPT | -0.30256 | 3.42453 | -2.59159 | 0.011834 | 0.09134 | -3.09487 | 1 |
| SESN2 | -0.08543 | 1.867341 | -2.59113 | 0.011848 | 0.09134 | -3.09591 | 0 |
| TPM4 | -0.16075 | 3.470236 | -2.58825 | 0.011938 | 0.091717 | -3.10246 | 1 |
| KCMF1 | -0.09982 | 3.010361 | -2.58402 | 0.012071 | 0.092373 | -3.11206 | 1 |
| TCN1 | -0.10869 | 1.374594 | -2.57593 | 0.012329 | 0.093815 | -3.1304 | 4 |
| TSHZ3 | -0.09939 | 1.75343 | -2.56996 | 0.012523 | 0.094754 | -3.14392 | 0 |
| USB1 | -0.08498 | 2.530823 | -2.56188 | 0.012789 | 0.095809 | -3.16215 | 0 |
| GRINA | -0.09709 | 3.004228 | -2.55728 | 0.012944 | 0.096389 | -3.17252 | 1 |
| HLX | -0.09653 | 2.095465 | -2.55254 | 0.013104 | 0.097134 | -3.18319 | 2 |
| DHX34 | -0.08824 | 1.929124 | -2.54878 | 0.013232 | 0.097742 | -3.19162 | 0 |
| CCDC71L | -0.07944 | 2.110067 | -2.54677 | 0.013302 | 0.097975 | -3.19613 | 1 |
| COLGALT1 | -0.15329 | 2.600856 | -2.53996 | 0.013539 | 0.098971 | -3.2114 | 1 |
| SCN1B | -0.18594 | 2.121391 | -2.5362 | 0.013671 | 0.099563 | -3.2198 | 1 |
| FKBP8 | -0.10789 | 2.596949 | -2.53138 | 0.013843 | 0.100145 | -3.23058 | 1 |
| ARF4 | -0.12184 | 2.957536 | -2.52978 | 0.0139 | 0.100349 | -3.23415 | 1 |
| CR1 | -0.14139 | 1.651296 | -2.51174 | 0.014562 | 0.103642 | -3.2743 | 2 |
| ROR1 | -0.1154 | 2.820446 | -2.50962 | 0.014641 | 0.103904 | -3.279 | 0 |
| RALBP1 | -0.15686 | 2.639473 | -2.50793 | 0.014705 | 0.104101 | -3.28274 | 1 |
| PNPLA6 | -0.08168 | 2.099136 | -2.50076 | 0.014978 | 0.105141 | -3.29861 | 0 |
| TKT | -0.11664 | 2.617972 | -2.5004 | 0.014992 | 0.105174 | -3.29941 | 1 |
| CLTB | -0.07929 | 3.284363 | -2.49726 | 0.015113 | 0.105665 | -3.30634 | 1 |
| SPI1 | -0.0985 | 2.318593 | -2.49655 | 0.01514 | 0.105771 | -3.3079 | 3 |
| NNMT | -0.16369 | 2.754558 | -2.49571 | 0.015173 | 0.105959 | -3.30978 | 1 |
| SLC36A4 | -0.12837 | 2.014965 | -2.49457 | 0.015218 | 0.106057 | -3.31228 | 1 |
| PTBP3 | -0.12769 | 2.69106 | -2.49209 | 0.015314 | 0.106392 | -3.31775 | 1 |
| USP12 | -0.24251 | 2.727067 | -2.48648 | 0.015536 | 0.107503 | -3.3301 | 1 |
| FAM89B | -0.09818 | 2.058909 | -2.485 | 0.015595 | 0.107783 | -3.33337 | 5 |
| RCN3 | -0.14256 | 2.035157 | -2.47572 | 0.015968 | 0.109498 | -3.35373 | 0 |
| TOM1 | -0.09497 | 2.776549 | -2.47254 | 0.016098 | 0.10966 | -3.36072 | 1 |
| TNFAIP8L3 | -0.16278 | 2.045377 | -2.47226 | 0.01611 | 0.109696 | -3.36133 | 2 |
| ZNF592 | -0.08956 | 2.810645 | -2.47074 | 0.016172 | 0.109949 | -3.36466 | 0 |
| MBD6 | -0.1128 | 2.420354 | -2.46904 | 0.016242 | 0.110011 | -3.36837 | 0 |
| SNORD8 | -0.32742 | 2.337621 | -2.46899 | 0.016244 | 0.110011 | -3.36848 | 5 |
| C1R | -0.12987 | 3.14703 | -2.46704 | 0.016325 | 0.110377 | -3.37275 | 1 |
| ABHD5 | -0.12409 | 2.811798 | -2.46413 | 0.016446 | 0.110907 | -3.37911 | 1 |
| BIRC7 | -0.08195 | 2.08419 | -2.45439 | 0.016858 | 0.11264 | -3.40035 | 0 |
| NETO2 | -0.12115 | 1.856451 | -2.45179 | 0.016969 | 0.113092 | -3.406 | 3 |
| SVIP | -0.15917 | 2.883831 | -2.44841 | 0.017115 | 0.11368 | -3.41336 | 1 |
| PLBD1 | -0.16354 | 2.57522 | -2.44575 | 0.017231 | 0.114098 | -3.41913 | 0 |
| ACSL4 | -0.12632 | 2.604887 | -2.44336 | 0.017335 | 0.114378 | -3.42433 | 0 |
| TECPR2 | -0.09903 | 2.252972 | -2.43926 | 0.017516 | 0.115037 | -3.4332 | 1 |
| SMOX | -0.08944 | 1.858754 | -2.43653 | 0.017637 | 0.115634 | -3.43911 | 0 |
| CDT1 | -0.09326 | 1.867595 | -2.43208 | 0.017836 | 0.116591 | -3.44875 | 2 |
| NRP2 | -0.09979 | 1.996842 | -2.42653 | 0.018087 | 0.117527 | -3.46072 | 0 |
| ST3GAL2 | -0.09874 | 2.239199 | -2.42556 | 0.018131 | 0.117646 | -3.46282 | 2 |
| MMP9 | -0.1024 | 1.822763 | -2.42554 | 0.018132 | 0.117646 | -3.46286 | 4 |

| | | | | | | | |
|---|---|---|---|---|---|---|---|
| ELANE | -0.12592 | 1.934532 | -2.42347 | 0.018227 | 0.118128 | -3.46731 | 3 |
| CLIC1 | -0.10786 | 3.457228 | -2.41869 | 0.018447 | 0.119114 | -3.4776 | 1 |
| SERPINB1C | -0.08932 | 1.348485 | -2.41441 | 0.018646 | 0.119825 | -3.4868 | 0 |
| BTBD10 | -0.13429 | 2.557676 | -2.41355 | 0.018686 | 0.119849 | -3.48866 | 0 |
| ITGB3 | -0.22933 | 2.007886 | -2.40707 | 0.018993 | 0.120772 | -3.50257 | 1 |
| CD24 | -0.19908 | 1.745584 | -2.39652 | 0.0195 | 0.122552 | -3.52513 | 0 |
| JAM3 | -0.0979 | 2.864552 | -2.39505 | 0.019572 | 0.12284 | -3.52826 | 0 |
| HIF1A | -0.20941 | 3.197366 | -2.39345 | 0.01965 | 0.123243 | -3.53167 | 1 |
| IRAK4 | -0.10659 | 1.980735 | -2.39198 | 0.019722 | 0.123534 | -3.5348 | 1 |
| IFITM3 | -0.11028 | 3.834883 | -2.39104 | 0.019769 | 0.123632 | -3.53682 | 1 |
| ARHGEF12 | -0.19788 | 3.290473 | -2.38875 | 0.019882 | 0.124184 | -3.54169 | 0 |
| TADA3 | -0.07864 | 2.431401 | -2.38823 | 0.019907 | 0.124233 | -3.54279 | 1 |
| VSTM1 | -0.09349 | 1.603343 | -2.38636 | 0.02 | 0.1246 | -3.54677 | 2 |
| FAM20C | -0.1211 | 2.117769 | -2.3849 | 0.020073 | 0.124848 | -3.54988 | 0 |
| MAPRE1 | -0.18306 | 3.24727 | -2.38222 | 0.020208 | 0.125544 | -3.55557 | 1 |
| PAQR6 | -0.09447 | 2.071618 | -2.38215 | 0.020211 | 0.125544 | -3.55571 | 0 |
| SCYL2 | -0.20858 | 2.708639 | -2.38014 | 0.020312 | 0.125772 | -3.55999 | 1 |
| PGM2L1 | -0.09948 | 1.605678 | -2.37903 | 0.020368 | 0.125987 | -3.56234 | 3 |
| DRAM1 | -0.12596 | 1.953746 | -2.37743 | 0.020449 | 0.126353 | -3.56574 | 1 |
| MGAM | -0.12744 | 1.470676 | -2.37047 | 0.020806 | 0.127522 | -3.58049 | 4 |
| IGF2BP2 | -0.13661 | 2.187053 | -2.37 | 0.02083 | 0.127623 | -3.58148 | 1 |
| HOOK3 | -0.1489 | 2.682005 | -2.36569 | 0.021054 | 0.128631 | -3.59058 | 0 |
| MAF1 | -0.13206 | 3.142042 | -2.35965 | 0.021371 | 0.12994 | -3.60334 | 1 |
| MAP4K4 | -0.14853 | 3.086945 | -2.35201 | 0.021778 | 0.131632 | -3.61942 | 0 |
| GIT1 | -0.10225 | 2.448563 | -2.3369 | 0.022603 | 0.134848 | -3.65108 | 1 |
| ARPC3 | -0.15152 | 3.069584 | -2.32807 | 0.023099 | 0.136535 | -3.66951 | 1 |
| CYTH4 | -0.10198 | 1.89411 | -2.31976 | 0.023574 | 0.138093 | -3.68681 | 4 |
| HK2 | -0.23097 | 2.213118 | -2.31816 | 0.023666 | 0.138525 | -3.69014 | 0 |
| ECE1 | -0.09632 | 3.030142 | -2.31504 | 0.023847 | 0.139228 | -3.69661 | 1 |
| SAMSN1 | -0.14251 | 1.887499 | -2.31349 | 0.023938 | 0.139477 | -3.69982 | 1 |
| HMGB2 | -0.11759 | 2.879881 | -2.31226 | 0.024009 | 0.13971 | -3.70237 | 0 |
| MIIP | -0.08833 | 2.004368 | -2.31069 | 0.024102 | 0.139906 | -3.70563 | 0 |
| JMJD6 | -0.15739 | 2.789732 | -2.30993 | 0.024146 | 0.140039 | -3.70719 | 1 |
| CRB3 | -0.10352 | 1.981891 | -2.30325 | 0.024543 | 0.141262 | -3.72101 | 0 |
| DYNC1LI2 | -0.13851 | 3.146021 | -2.29358 | 0.025127 | 0.143631 | -3.74096 | 0 |
| MXD3 | -0.082 | 2.19672 | -2.29126 | 0.025268 | 0.144251 | -3.74571 | 0 |
| RIOK3 | -0.17331 | 2.937376 | -2.28673 | 0.025547 | 0.14538 | -3.75502 | 1 |
| FGD4 | -0.13876 | 2.649686 | -2.28519 | 0.025643 | 0.145772 | -3.75819 | 0 |
| WBP2 | -0.11102 | 3.354125 | -2.285 | 0.025655 | 0.145791 | -3.75858 | 1 |
| POLD4 | -0.08169 | 2.95275 | -2.26945 | 0.026638 | 0.149675 | -3.7904 | 1 |
| ZDHHC19 | -0.09254 | 1.642818 | -2.26361 | 0.027015 | 0.151018 | -3.80229 | 4 |
| MT1G | -0.25451 | 3.32669 | -2.26296 | 0.027057 | 0.151206 | -3.8036 | 1 |
| IGFLR1 | -0.08727 | 1.858319 | -2.25823 | 0.027368 | 0.15221 | -3.81324 | 3 |
| ZFAND2A | -0.16906 | 2.430627 | -2.25716 | 0.027437 | 0.152405 | -3.8154 | 0 |
| TNNI1 | -0.23044 | 2.4109 | -2.25578 | 0.027529 | 0.152569 | -3.81821 | 0 |
| CBX3 | -0.09901 | 2.831003 | -2.24255 | 0.028416 | 0.155409 | -3.84499 | 1 |
| BLOC1S2 | -0.08711 | 2.415497 | -2.23829 | 0.028708 | 0.156527 | -3.85359 | 1 |
| GPR160 | -0.10199 | 1.750261 | -2.23528 | 0.028915 | 0.157324 | -3.85964 | 3 |
| MARCO | -0.14985 | 1.861212 | -2.22584 | 0.029573 | 0.159161 | -3.87862 | 1 |
| WNT9A | -0.117 | 1.903526 | -2.22299 | 0.029775 | 0.159714 | -3.88435 | 0 |
| SLC9A1 | -0.12297 | 2.479962 | -2.22021 | 0.029972 | 0.160469 | -3.8899 | 0 |

| | | | | | | | |
|---|---|---|---|---|---|---|---|
| MOSPD3 | -0.08525 | 2.295683 | -2.21992 | 0.029993 | 0.160534 | -3.8905 | 0 |
| CLEC4D | -0.10974 | 1.297436 | -2.21851 | 0.030094 | 0.160843 | -3.89331 | 0 |
| ZNF354A | -0.08775 | 2.224438 | -2.20949 | 0.030745 | 0.162568 | -3.91133 | 0 |
| ZNF410 | -0.08974 | 2.397901 | -2.20618 | 0.030988 | 0.163207 | -3.91793 | 1 |
| DYRK3 | -0.06456 | 2.100125 | -2.20544 | 0.031042 | 0.163396 | -3.91941 | 0 |
| DBN1 | -0.10129 | 2.387275 | -2.20509 | 0.031067 | 0.16343 | -3.92009 | 2 |
| TMEM234 | -0.06244 | 1.992209 | -2.20465 | 0.0311 | 0.163545 | -3.92097 | 0 |
| FBXO38 | -0.11037 | 2.472824 | -2.20267 | 0.031246 | 0.164026 | -3.92491 | 0 |
| LRPAP1 | -0.09398 | 3.295588 | -2.2017 | 0.031318 | 0.164255 | -3.92684 | 0 |
| PEF1 | -0.06239 | 2.679384 | -2.20113 | 0.03136 | 0.164379 | -3.92798 | 0 |
| SEMA6B | -0.06438 | 2.329959 | -2.19824 | 0.031575 | 0.164809 | -3.93372 | 1 |
| GTPBP1 | -0.08368 | 2.686667 | -2.19503 | 0.031816 | 0.165223 | -3.94008 | 0 |
| FGR | -0.10519 | 2.077154 | -2.19116 | 0.032107 | 0.166054 | -3.94775 | 4 |
| NADSYN1 | -0.08269 | 2.274306 | -2.18686 | 0.032435 | 0.167302 | -3.95626 | 1 |
| GSTO1 | -0.128 | 3.111369 | -2.18468 | 0.032602 | 0.167897 | -3.96057 | 1 |
| BEND2 | -0.08352 | 1.360085 | -2.18323 | 0.032713 | 0.168241 | -3.96343 | 0 |
| NFAT5 | -0.14139 | 2.730895 | -2.18019 | 0.032948 | 0.169052 | -3.96943 | 0 |
| APOC1 | -0.16655 | 2.334611 | -2.1758 | 0.03329 | 0.170357 | -3.97808 | 2 |
| C11orf58 | -0.14849 | 3.400023 | -2.17475 | 0.033372 | 0.170527 | -3.98015 | 1 |
| HGF | -0.11171 | 1.661354 | -2.173 | 0.033509 | 0.171027 | -3.98358 | 0 |
| CHD2 | -0.12968 | 2.963028 | -2.17208 | 0.033581 | 0.171145 | -3.98538 | 0 |
| IRAK3 | -0.15058 | 2.635251 | -2.15896 | 0.034629 | 0.175059 | -4.01111 | 1 |
| ANO10 | -0.09352 | 2.044789 | -2.15515 | 0.034939 | 0.176102 | -4.01856 | 3 |
| KCTD20 | -0.1258 | 2.30707 | -2.14869 | 0.035469 | 0.17761 | -4.03115 | 1 |
| SLCO4C1 | -0.08451 | 1.331929 | -2.14755 | 0.035564 | 0.177821 | -4.03337 | 0 |
| GSDMD | -0.06869 | 1.956099 | -2.14415 | 0.035846 | 0.178627 | -4.03998 | 2 |
| IFT20 | -0.11917 | 2.831537 | -2.1415 | 0.036067 | 0.179372 | -4.04512 | 0 |
| SLC2A5 | -0.07864 | 1.918438 | -2.14099 | 0.036111 | 0.179501 | -4.04612 | 0 |
| NFKBIZ | -0.17105 | 2.46938 | -2.13867 | 0.036305 | 0.179948 | -4.05061 | 1 |
| PGM1 | -0.15187 | 3.395163 | -2.13579 | 0.036549 | 0.180459 | -4.0562 | 1 |
| LPCAT2 | -0.09632 | 2.42925 | -2.13573 | 0.036554 | 0.180459 | -4.05631 | 0 |
| USP32 | -0.14757 | 2.580163 | -2.13317 | 0.036772 | 0.181082 | -4.06127 | 1 |
| ATF6 | -0.16199 | 2.908853 | -2.13221 | 0.036854 | 0.181325 | -4.06313 | 1 |
| SNX10 | -0.12111 | 1.619427 | -2.13047 | 0.037003 | 0.181903 | -4.06649 | 2 |
| WDR41 | -0.06749 | 2.571855 | -2.12571 | 0.037413 | 0.183354 | -4.07569 | 0 |
| CPD | -0.18588 | 2.804693 | -2.12451 | 0.037517 | 0.183511 | -4.078 | 1 |
| CGRRF1 | -0.09998 | 1.984912 | -2.12423 | 0.037541 | 0.18357 | -4.07854 | 0 |
| LDHA | -0.14184 | 3.309127 | -2.12183 | 0.03775 | 0.184156 | -4.08316 | 1 |
| BUD31 | -0.12923 | 2.87867 | -2.12155 | 0.037775 | 0.184196 | -4.0837 | 1 |
| SLC16A6 | -0.10948 | 1.678376 | -2.12065 | 0.037853 | 0.184349 | -4.08543 | 0 |
| IL18R1 | -0.10102 | 1.958586 | -2.11565 | 0.038293 | 0.185687 | -4.09505 | 1 |
| SHARPIN | -0.06651 | 2.776349 | -2.10859 | 0.038921 | 0.187485 | -4.1086 | 0 |
| CEACAM6 | -0.09133 | 1.62724 | -2.10752 | 0.039017 | 0.187844 | -4.11064 | 0 |
| LILRA3 | -0.13883 | 1.883177 | -2.10731 | 0.039036 | 0.18788 | -4.11105 | 4 |
| NAT1 | -0.09493 | 1.57412 | -2.1047 | 0.039271 | 0.18854 | -4.11604 | 0 |
| FCN3 | -0.16251 | 2.493337 | -2.10084 | 0.039621 | 0.189602 | -4.12342 | 3 |
| HOMER3 | -0.07112 | 2.448764 | -2.09434 | 0.040216 | 0.191341 | -4.13582 | 0 |
| TSEN34 | -0.05854 | 2.076868 | -2.08975 | 0.040641 | 0.192072 | -4.14455 | 0 |
| RASL11A | -0.22093 | 1.95938 | -2.0892 | 0.040692 | 0.192154 | -4.14559 | 1 |
| TNFAIP6 | -0.44816 | 2.232062 | -2.08229 | 0.041341 | 0.19448 | -4.15871 | 1 |
| CREM | -0.09124 | 2.174264 | -2.07715 | 0.041828 | 0.195982 | -4.16843 | 1 |

| | | | | | | | |
|---|---|---|---|---|---|---|---|
| RAB3IL1 | -0.06204 | 2.054389 | -2.07268 | 0.042257 | 0.197257 | -4.17689 | 0 |
| TMCO3 | -0.06564 | 2.373837 | -2.06219 | 0.043277 | 0.199666 | -4.19664 | 0 |
| SLC22A2 | -0.09509 | 1.534004 | -2.0598 | 0.043512 | 0.200436 | -4.20113 | 2 |
| ITGAX | -0.09747 | 1.978151 | -2.05921 | 0.04357 | 0.200556 | -4.20223 | 4 |
| PTGS1 | -0.11478 | 1.916563 | -2.05508 | 0.04398 | 0.201794 | -4.20998 | 2 |
| SHKBP1 | -0.07153 | 2.13641 | -2.05382 | 0.044106 | 0.20218 | -4.21234 | 3 |
| PPP1R15A | -0.12775 | 3.112872 | -2.05337 | 0.04415 | 0.202256 | -4.21317 | 0 |
| ARID4A | -0.0606 | 2.572075 | -2.05123 | 0.044365 | 0.20292 | -4.21718 | 0 |
| BROX | -0.07959 | 2.640915 | -2.05046 | 0.044442 | 0.203026 | -4.21862 | 0 |
| TPGS2 | -0.09456 | 2.669566 | -2.04582 | 0.044911 | 0.204455 | -4.2273 | 0 |
| NT5C2 | -0.13688 | 2.743839 | -2.04478 | 0.045016 | 0.204717 | -4.22923 | 0 |
| CDV3 | -0.09385 | 3.036503 | -2.04283 | 0.045215 | 0.205198 | -4.23287 | 1 |
| RPS6KA4 | -0.0579 | 1.747479 | -2.03826 | 0.045683 | 0.206573 | -4.24138 | 0 |
| RAB8B | -0.13928 | 2.720101 | -2.03716 | 0.045797 | 0.206713 | -4.24342 | 1 |
| AMFR | -0.10648 | 2.81369 | -2.02836 | 0.046712 | 0.209546 | -4.25976 | 0 |
| SLITRK4 | -0.12025 | 1.713889 | -2.02053 | 0.04754 | 0.211756 | -4.27423 | 0 |
| BAG4 | -0.06194 | 2.503429 | -2.01747 | 0.047867 | 0.21243 | -4.27988 | 0 |
| ZDHHC3 | -0.0709 | 2.785059 | -2.01629 | 0.047993 | 0.212778 | -4.28205 | 1 |
| NT5DC3 | -0.06486 | 1.593765 | -2.01602 | 0.048023 | 0.212805 | -4.28255 | 1 |
| CKAP4 | -0.05566 | 3.044826 | -2.01529 | 0.048101 | 0.212844 | -4.28389 | 0 |
| UBA6 | -0.11923 | 2.261462 | -2.01304 | 0.048344 | 0.213321 | -4.28804 | 0 |
| NLGN2 | -0.08994 | 1.845126 | -2.01148 | 0.048513 | 0.213892 | -4.29091 | 2 |
| DNTTIP2 | -0.12061 | 2.738957 | -2.01054 | 0.048615 | 0.21418 | -4.29264 | 1 |
| LRRN1 | -0.09268 | 1.830681 | -2.01021 | 0.048651 | 0.214219 | -4.29325 | 2 |
| TCIRG1 | -0.08343 | 2.374626 | -2.00902 | 0.04878 | 0.214582 | -4.29542 | 4 |
| MLKL | -0.13485 | 1.868184 | -2.00818 | 0.048872 | 0.214718 | -4.29698 | 1 |
| EDEM1 | -0.06817 | 2.08413 | -2.00663 | 0.049041 | 0.215082 | -4.29982 | 1 |
| APPL2 | -0.14642 | 2.446368 | -2.00504 | 0.049215 | 0.215575 | -4.30273 | 0 |
| HSPA13 | -0.11041 | 2.649112 | -2.00313 | 0.049426 | 0.216172 | -4.30624 | 1 |
| INHBA | -0.1163 | 1.750525 | -1.99918 | 0.049862 | 0.217318 | -4.31346 | 0 |
