# Supplementary material for: Exploration of the Shared Gene Signatures between Myocardium and Blood in Sepsis: Evidence from Bioinformatics Analysis
Source: Biomed Res Int. 2022 Aug 6;2022:3690893. doi: 10.1155/2022/3690893 (PMC9375705; doi:10.1155/2022/3690893)
Supplement: Supplementary Materials — Supplement Figure S1. Correlations between different module memberships generated by weighted correlation network analysis (WGCNA). The gene significances were calculated and are presented in a scatter plot. Correlation coefficients and p-values are shown on the top of the figure. The blue, brown, grey, and turquoise modules were found to be linearly correlated with gene significance. Figure S2. The x-axis of the bar plot indicates the possible clustering method suggested by k-means clustering analysis, and the y-axis presents the evaluation scores of the different clustering methods. The k-means clustering analysis suggests that the best classification is dichotomous. B. The eigenvalue decreases rapidly as the component number increases at the beginning, especially when the first two components are added. The decrease in the eigenvalue was less obvious when more than three components were added. A scree plot shows that two components can well describe the characteristics of the groups clustered by the clustering analysis. Figure S3. Nomograph displaying the risk score of each risk factor when six genes were integrated with the age and appach II score, including SMU1, SP100, and ARHGAP25, which contribute great weight to mortality. Calibration curve in GSE54514, which shows the good fit of our model. The mean absolute error is acceptable (mean absolute error =0.033). Table S1. A total of 1,049 DEGs changed in the same way in both the myocardium and blood datasets, including 549 genes upregulated and 500 genes downregulated. There were 325 genes in the blue module, 116 genes in the brown module, 305 genes in the turquoise module, and 261 genes in the gray module. Table S2. Detailed results of Gene Ontology (GO) analysis and GeneSet Enrichment Analysis (GSEA) of the turquoise and blue modules, as supplementary material for Figure 2. This table displays all the GO analysis pathways, including biological process (BP), cell component (CC), and molecular function (MF), as [file 3690893.f1.zip › Table-S2.pdf]

	ONTOLOGY	ID	Descripti	GeneRatic	BgRatio	pvalue	p.adjust	qvalue
GO:003497	BP	GO:003497	response	41/1016	296/18862	2.67E-08	0.000145	0.000119
GO:200123	BP	GO:200123	regulatic	44/1016	348/18862	1.26E-07	0.000239	0.000197
GO:007137	BP	GO:007137	cellular	42/1016	325/18862	1.32E-07	0.000239	0.000197
GO:003596	BP	GO:003596	response	31/1016	206/18862	2.07E-07	0.00028	0.00023
GO:003596	BP	GO:003596	cellular	27/1016	168/18862	3.27E-07	0.000341	0.000281
GO:000688	BP	GO:000688	cellular	12/1016	38/18862	4.11E-07	0.000341	0.000281
GO:200123	BP	GO:200123	negative	32/1016	224/18862	4.41E-07	0.000341	0.000281
GO:190165	BP	GO:190165	cellular	46/1016	391/18862	5.42E-07	0.000367	0.000302
GO:000698	BP	GO:000698	response	28/1016	185/18862	7.04E-07	0.000416	0.000343
GO:005506	BP	GO:005506	zinc ion	12/1016	40/18862	7.68E-07	0.000416	0.000343
GO:001905	BP	GO:001905	viral lif	42/1016	348/18862	8.56E-07	0.000421	0.000347
GO:000989	BP	GO:000989	positive	50/1016	450/18862	9.86E-07	0.000442	0.000364
GO:007128	BP	GO:007128	cellular	10/1016	28/18862	1.06E-06	0.000442	0.000364
GO:004691	BP	GO:004691	cellular	21/1016	118/18862	1.20E-06	0.000465	0.000384
GO:003462	BP	GO:003462	cellular	24/1016	149/18862	1.40E-06	0.000483	0.000398
GO:005507	BP	GO:005507	transitic	23/1016	139/18862	1.43E-06	0.000483	0.000398
GO:003649	BP	GO:003649	PERK-medi	9/1016	23/18862	1.52E-06	0.000483	0.000398
GO:003133	BP	GO:003133	positive	44/1016	384/18862	1.94E-06	0.000585	0.000482
GO:003109	BP	GO:003109	stress-ac	35/1016	276/18862	2.25E-06	0.000641	0.000528
GO:007127	BP	GO:007127	cellular	11/1016	38/18862	3.29E-06	0.000854	0.000704
GO:004668	BP	GO:004668	response	14/1016	61/18862	3.31E-06	0.000854	0.000704
GO:004225	BP	GO:004225	ribosome	37/1016	307/18862	3.92E-06	0.000897	0.000739
GO:009719	BP	GO:009719	intrinsic	35/1016	283/18862	3.99E-06	0.000897	0.000739
GO:003096	BP	GO:003096	endoplasm	21/1016	127/18862	4.09E-06	0.000897	0.000739
GO:007134	BP	GO:007134	cellular	26/1016	180/18862	4.23E-06	0.000897	0.000739
GO:004343	BP	GO:004343	response	47/1016	435/18862	4.47E-06	0.000897	0.000739
GO:004852	BP	GO:004852	negative	17/1016	89/18862	4.55E-06	0.000897	0.000739
GO:005140	BP	GO:005140	stress-ac	33/1016	261/18862	4.64E-06	0.000897	0.000739
GO:004578	BP	GO:004578	positive	46/1016	425/18862	5.38E-06	0.000958	0.00079
GO:190589	BP	GO:190589	regulatic	16/1016	81/18862	5.49E-06	0.000958	0.00079
GO:001027	BP	GO:001027	detoxific	7/1016	15/18862	5.66E-06	0.000958	0.00079
GO:199016	BP	GO:199016	stress re	7/1016	15/18862	5.66E-06	0.000958	0.00079
GO:007055	BP	GO:007055	response	28/1016	206/18862	6.08E-06	0.000998	0.000822
GO:014046	BP	GO:014046	integrate	9/1016	27/18862	7.15E-06	0.001139	0.000939
GO:004668	BP	GO:004668	response	11/1016	42/18862	9.60E-06	0.001482	0.001222
GO:007067	BP	GO:007067	response	12/1016	50/18862	1.01E-05	0.001482	0.001222
GO:007074	BP	GO:007074	response	12/1016	50/18862	1.01E-05	0.001482	0.001222
GO:200124	BP	GO:200124	negative	17/1016	95/18862	1.14E-05	0.001619	0.001334
GO:002240	BP	GO:002240	positive	33/1016	276/18862	1.52E-05	0.00206	0.001698
GO:006168	BP	GO:006168	detoxific	7/1016	17/18862	1.56E-05	0.00206	0.001698
GO:200124	BP	GO:200124	regulatic	23/1016	160/18862	1.61E-05	0.00206	0.001698
GO:004594	BP	GO:004594	positive	8/1016	23/18862	1.64E-05	0.00206	0.001698
GO:190438	BP	GO:190438	cellular	8/1016	23/18862	1.64E-05	0.00206	0.001698
GO:002261	BP	GO:002261	ribonucle	48/1016	473/18862	1.90E-05	0.00234	0.001928
GO:005170	BP	GO:005170	interacti	28/1016	219/18862	1.94E-05	0.00234	0.001928
GO:005123	BP	GO:005123	maintenan	36/1016	319/18862	2.28E-05	0.002645	0.00218
GO:007129	BP	GO:007129	cellular	8/1016	24/18862	2.34E-05	0.002645	0.00218
GO:004333	BP	GO:004333	response	12/1016	54/18862	2.35E-05	0.002645	0.00218
GO:009750	BP	GO:009750	stress re	7/1016	18/18862	2.43E-05	0.002659	0.002191
GO:007135	BP	GO:007135	cellular	11/1016	46/18862	2.46E-05	0.002659	0.002191
GO:000828	BP	GO:000828	insulin r	21/1016	143/18862	2.68E-05	0.002848	0.002347
GO:000150	BP	GO:000150	ossificat	42/1016	401/18862	2.99E-05	0.003117	0.002568
GO:199077	BP	GO:199077	response	8/1016	25/18862	3.28E-05	0.003348	0.002759

GO:004331	BP	GO:004331	neutrophi	48/1016	485/18862	3.62E-05	0.003623	0.002986
GO:000244	BP	GO:000244	neutrophi	49/1016	499/18862	3.68E-05	0.003623	0.002986
GO:000862	BP	GO:000862	extrinsic	15/1016	84/18862	3.77E-05	0.00365	0.003007
GO:003009	BP	GO:003009	myeloid c	43/1016	419/18862	3.92E-05	0.003728	0.003072
GO:003019	BP	GO:003019	extracell	41/1016	393/18862	4.06E-05	0.00379	0.003123
GO:004211	BP	GO:004211	T cell ac	47/1016	474/18862	4.15E-05	0.003809	0.003139
GO:000228	BP	GO:000228	neutrophi	48/1016	488/18862	4.23E-05	0.003814	0.003143
GO:004306	BP	GO:004306	extracell	41/1016	394/18862	4.30E-05	0.003818	0.003146
GO:000635	BP	GO:000635	regulatio	9/1016	33/18862	4.39E-05	0.003838	0.003162
GO:005109	BP	GO:005109	positive	31/1016	266/18862	4.48E-05	0.003851	0.003173
GO:007134	BP	GO:007134	cellular	11/1016	49/18862	4.62E-05	0.003912	0.003223
GO:004522	BP	GO:004522	external	41/1016	396/18862	4.83E-05	0.004021	0.003313
GO:005109	BP	GO:005109	regulatio	38/1016	357/18862	4.99E-05	0.00408	0.003362
GO:000181	BP	GO:000181	positive	44/1016	437/18862	5.05E-05	0.00408	0.003362
GO:007037	BP	GO:007037	ERK1 and	35/1016	320/18862	5.63E-05	0.004484	0.003695
GO:003434	BP	GO:003434	response	25/1016	197/18862	6.03E-05	0.00473	0.003898
GO:001907	BP	GO:001907	viral gen	19/1016	129/18862	6.13E-05	0.00474	0.003906
GO:005079	BP	GO:005079	regulatio	24/1016	186/18862	6.44E-05	0.004869	0.004012
GO:190303	BP	GO:190303	positive	28/1016	234/18862	6.47E-05	0.004869	0.004012
GO:004685	BP	GO:004685	regulatio	11/1016	51/18862	6.85E-05	0.005079	0.004185
GO:005165	BP	GO:005165	establish	43/1016	431/18862	7.56E-05	0.005532	0.004559
GO:004211	BP	GO:004211	neutrophi	48/1016	500/18862	7.71E-05	0.005569	0.004589
GO:003249	BP	GO:003249	response	35/1016	326/18862	8.21E-05	0.005847	0.004818
GO:007005	BP	GO:007005	intrinsic	12/1016	61/18862	8.41E-05	0.005895	0.004858
GO:005072	BP	GO:005072	regulatio	38/1016	366/18862	8.49E-05	0.005895	0.004858
GO:007037	BP	GO:007037	regulatio	33/1016	301/18862	8.67E-05	0.005945	0.004899
GO:003286	BP	GO:003286	cellular	27/1016	226/18862	8.92E-05	0.006033	0.004972
GO:190357	BP	GO:190357	negative	10/1016	44/18862	9.13E-05	0.006033	0.004972
GO:000910	BP	GO:000910	glycoprot	42/1016	421/18862	9.14E-05	0.006033	0.004972
GO:001050	BP	GO:001050	regulatio	35/1016	328/18862	9.28E-05	0.006053	0.004987
GO:000698	BP	GO:000698	ER-nucleu	11/1016	53/18862	9.93E-05	0.006211	0.005118
GO:000636	BP	GO:000636	rRNA proc	27/1016	228/18862	0.000104	0.006211	0.005118
GO:004217	BP	GO:004217	regulatio	39/1016	383/18862	0.000104	0.006211	0.005118
GO:003166	BP	GO:003166	response	44/1016	451/18862	0.000105	0.006211	0.005118
GO:200037	BP	GO:200037	regulatio	24/1016	192/18862	0.000107	0.006211	0.005118
GO:003650	BP	GO:003650	ERAD path	16/1016	102/18862	0.000107	0.006211	0.005118
GO:003097	BP	GO:003097	retrograd	8/1016	29/18862	0.000107	0.006211	0.005118
GO:006005	BP	GO:006005	angiogene	8/1016	29/18862	0.000107	0.006211	0.005118
GO:190351	BP	GO:190351	endoplasm	8/1016	29/18862	0.000107	0.006211	0.005118
GO:001088	BP	GO:001088	negative	7/1016	22/18862	0.000108	0.006211	0.005118
GO:000926	BP	GO:000926	cellular	21/1016	157/18862	0.000108	0.006211	0.005118
GO:190313	BP	GO:190313	mononucle	41/1016	411/18862	0.00011	0.006294	0.005186
GO:009719	BP	GO:009719	extrinsic	26/1016	217/18862	0.000114	0.006428	0.005297
GO:004662	BP	GO:004662	negative	9/1016	37/18862	0.000117	0.006514	0.005368
GO:004506	BP	GO:004506	regulatio	14/1016	83/18862	0.000129	0.007123	0.00587
GO:000724	BP	GO:000724	I-kappaB	31/1016	282/18862	0.000134	0.007307	0.006021
GO:190390	BP	GO:190390	regulatio	20/1016	148/18862	0.000135	0.007323	0.006034
GO:190223	BP	GO:190223	regulatio	8/1016	30/18862	0.000139	0.007475	0.006159
GO:004259	BP	GO:004259	response	24/1016	196/18862	0.000147	0.00776	0.006394
GO:005109	BP	GO:005109	regulatio	43/1016	444/18862	0.000148	0.00776	0.006394
GO:004390	BP	GO:004390	regulatio	24/1016	197/18862	0.000159	0.008291	0.006832
GO:005135	BP	GO:005135	positive	11/1016	56/18862	0.000167	0.00856	0.007054
GO:001991	BP	GO:001991	lipid sto	14/1016	85/18862	0.000168	0.00856	0.007054
GO:000715	BP	GO:000715	leukocyte	37/1016	366/18862	0.00018	0.009045	0.007453

GO:190007	BP	GO:190007	negative	9/1016	39/18862	0.00018	0.009045	0.007453
GO:005212	BP	GO:005212	movement	22/1016	175/18862	0.000187	0.009187	0.007571
GO:00321C	BP	GO:00321C	negative	39/1016	394/18862	0.000189	0.009187	0.007571
GO:00458E	BP	GO:00458E	positive	37/1016	367/18862	0.00019	0.009187	0.007571
GO:00099S	BP	GO:00099S	response	45/1016	477/18862	0.00019	0.009187	0.007571
GO:004214	BP	GO:004214	cellular	10/1016	48/18862	0.000198	0.009434	0.007774
GO:19039C	BP	GO:19039C	negative	7/1016	24/18862	0.000199	0.009434	0.007774
GO:00224C	BP	GO:00224C	regulatic	42/1016	437/18862	0.000207	0.009741	0.008026
GO:001607	BP	GO:001607	rRNA meta	27/1016	238/18862	0.000212	0.009873	0.008135
GO:19059E	BP	GO:19059E	negative	12/1016	67/18862	0.000215	0.009873	0.008135
GO:00068S	BP	GO:00068S	retrograd	14/1016	87/18862	0.000216	0.009873	0.008135
GO:004677	BP	GO:004677	protein a	26/1016	226/18862	0.00022	0.009873	0.008135
GO:00075E	BP	GO:00075E	female pr	22/1016	177/18862	0.000221	0.009873	0.008135
GO:007134	BP	GO:007134	cellular	22/1016	177/18862	0.000221	0.009873	0.008135
GO:00328E	BP	GO:00328E	response	30/1016	278/18862	0.000235	0.010448	0.00861
GO:00105C	BP	GO:00105C	positive	17/1016	120/18862	0.000237	0.010453	0.008614
GO:00063E	BP	GO:00063E	transcrip	12/1016	68/18862	0.000249	0.010852	0.008943
GO:00022E	BP	GO:00022E	response	35/1016	346/18862	0.000263	0.011383	0.00938
GO:004542	BP	GO:004542	regulatic	11/1016	59/18862	0.000271	0.011566	0.009531
GO:190204	BP	GO:190204	regulatic	11/1016	59/18862	0.000271	0.011566	0.009531
GO:001657	BP	GO:001657	protein d	30/1016	283/18862	0.00032	0.01354	0.011157
GO:004312	BP	GO:004312	positive	22/1016	182/18862	0.000328	0.013773	0.011349
GO:004592	BP	GO:004592	negative	27/1016	245/18862	0.000339	0.014108	0.011625
GO:00330C	BP	GO:00330C	positive	6/1016	19/18862	0.000356	0.014664	0.012084
GO:00510S	BP	GO:00510S	positive	20/1016	159/18862	0.000357	0.014664	0.012084
GO:00801E	BP	GO:00801E	regulatic	11/1016	61/18862	0.000367	0.014953	0.012322
GO:00703C	BP	GO:00703C	regulatic	22/1016	184/18862	0.000383	0.01546	0.012739
GO:00330C	BP	GO:00330C	positive	5/1016	13/18862	0.000402	0.015896	0.013098
GO:00433C	BP	GO:00433C	positive	5/1016	13/18862	0.000402	0.015896	0.013098
GO:007122	BP	GO:007122	cellular	23/1016	197/18862	0.000402	0.015896	0.013098
GO:003052	BP	GO:003052	intracell	29/1016	274/18862	0.00041	0.016095	0.013263
GO:00725C	BP	GO:00725C	cellular	44/1016	480/18862	0.000415	0.0161	0.013267
GO:190134	BP	GO:190134	regulatic	34/1016	341/18862	0.000416	0.0161	0.013267
GO:007052	BP	GO:007052	platelet	11/1016	62/18862	0.000425	0.016325	0.013452
GO:00506E	BP	GO:00506E	nucleic a	23/1016	198/18862	0.000432	0.016373	0.013492
GO:00506E	BP	GO:00506E	RNA trans	23/1016	198/18862	0.000432	0.016373	0.013492
GO:003021	BP	GO:003021	erythrocy	16/1016	115/18862	0.00044	0.016562	0.013648
GO:001072	BP	GO:001072	negative	21/1016	174/18862	0.000453	0.016929	0.01395
GO:00104S	BP	GO:00104S	proteasom	44/1016	483/18862	0.000473	0.017451	0.01438
GO:004512	BP	GO:004512	regulatic	9/1016	44/18862	0.000474	0.017451	0.01438
GO:00063E	BP	GO:00063E	transcrip	22/1016	187/18862	0.000479	0.017527	0.014443
GO:00068C	BP	GO:00068C	nitric ox	12/1016	73/18862	0.00049	0.017809	0.014675
GO:003247	BP	GO:003247	regulatic	17/1016	128/18862	0.000514	0.018545	0.015282
GO:00512E	BP	GO:00512E	establish	23/1016	201/18862	0.000535	0.019138	0.01577
GO:004507	BP	GO:004507	negative	10/1016	54/18862	0.00054	0.019138	0.01577
GO:003022	BP	GO:003022	monocyte	8/1016	36/18862	0.000541	0.019138	0.01577
GO:19059E	BP	GO:19059E	regulatic	22/1016	189/18862	0.000555	0.019406	0.015991
GO:00159E	BP	GO:00159E	nucleobas	27/1016	253/18862	0.000562	0.019406	0.015991
GO:00018S	BP	GO:00018S	maternal	7/1016	28/18862	0.000562	0.019406	0.015991
GO:00326C	BP	GO:00326C	type I in	17/1016	129/18862	0.000563	0.019406	0.015991
GO:00091C	BP	GO:00091C	glycoprot	34/1016	347/18862	0.00057	0.019531	0.016094
GO:00341C	BP	GO:00341C	homotypic	13/1016	85/18862	0.000595	0.020218	0.01666
GO:00603S	BP	GO:00603S	growth hc	5/1016	14/18862	0.000597	0.020218	0.01666
GO:00466E	BP	GO:00466E	alpha-bet	15/1016	107/18862	0.000606	0.020379	0.016793

GO:00457€	BP	GO:00457€	regulatic	33/1016	335/18862	0.000621	0.020754	0.017102
GO:00435З	BP	GO:00435З	positive	12/1016	75/18862	0.000631	0.020754	0.017102
GO:19021C	BP	GO:19021C	positive	19/1016	154/18862	0.000632	0.020754	0.017102
GO:19037C	BP	GO:19037C	positive	19/1016	154/18862	0.000632	0.020754	0.017102
GO:004001	BP	GO:004001	negative	36/1016	377/18862	0.000643	0.020973	0.017283
GO:006104	BP	GO:006104	vascular	6/1016	21/18862	0.000649	0.021046	0.017342
GO:00341C	BP	GO:00341C	regulatic	13/1016	86/18862	0.000667	0.021417	0.017648
GO:007084	BP	GO:007084	response	9/1016	46/18862	0.000668	0.021417	0.017648
GO:00458C	BP	GO:00458C	positive	14/1016	97/18862	0.000678	0.021586	0.017788
GO:004312	BP	GO:004312	regulatic	26/1016	244/18862	0.000721	0.022828	0.01881
GO:00100З	BP	GO:00100З	response	34/1016	352/18862	0.000734	0.022979	0.018935
GO:005127	BP	GO:005127	negative	34/1016	352/18862	0.000734	0.022979	0.018935
GO:003287	BP	GO:003287	regulatic	21/1016	181/18862	0.000765	0.023699	0.019528
GO:00098Ç	BP	GO:00098Ç	negative	31/1016	312/18862	0.000777	0.023699	0.019528
GO:00341C	BP	GO:00341C	erythrocy	16/1016	121/18862	0.000778	0.023699	0.019528
GO:00069C	BP	GO:00069C	phagocytc	36/1016	381/18862	0.000779	0.023699	0.019528
GO:000244	BP	GO:000244	mast cell	9/1016	47/18862	0.000788	0.023699	0.019528
GO:003572	BP	GO:003572	interleuk	9/1016	47/18862	0.000788	0.023699	0.019528
GO:004564	BP	GO:004564	regulatic	9/1016	47/18862	0.000788	0.023699	0.019528
GO:00462C	BP	GO:00462C	nitric ox	12/1016	77/18862	0.000804	0.024026	0.019798
GO:00022€	BP	GO:00022€	myeloid c	18/1016	145/18862	0.000808	0.024026	0.019798
GO:007121	BP	GO:007121	cellular	25/1016	233/18862	0.000818	0.024198	0.01994
GO:190342	BP	GO:190342	regulatic	14/1016	99/18862	0.000834	0.024445	0.020143
GO:00364Ç	BP	GO:00364Ç	IRE1-medi	11/1016	67/18862	0.000841	0.024445	0.020143
GO:001004	BP	GO:001004	response	10/1016	57/18862	0.000842	0.024445	0.020143
GO:007064	BP	GO:007064	protein m	30/1016	300/18862	0.000844	0.024445	0.020143
GO:00328Ç	BP	GO:00328Ç	negative	5/1016	15/18862	0.000857	0.024537	0.020219
GO:20000Ꞓ	BP	GO:20000Ꞓ	positive	5/1016	15/18862	0.000857	0.024537	0.020219
GO:000222	BP	GO:000222	pattern r	23/1016	208/18862	0.000862	0.024537	0.020219
GO:004544	BP	GO:004544	fat cell	24/1016	221/18862	0.00087	0.024537	0.020219
GO:00064C	BP	GO:00064C	RNA local	25/1016	234/18862	0.00087	0.024537	0.020219
GO:000661	BP	GO:000661	protein ε	21/1016	183/18862	0.000883	0.024765	0.020407
GO:20010Ꞓ	BP	GO:20010Ꞓ	reactive	12/1016	78/18862	0.000905	0.025247	0.020805
GO:007121	BP	GO:007121	cellular	23/1016	209/18862	0.000921	0.025343	0.020883
GO:003252	BP	GO:003252	protein ε	9/1016	48/18862	0.000924	0.025343	0.020883
GO:00433З	BP	GO:00433З	response	9/1016	48/18862	0.000924	0.025343	0.020883
GO:00725C	BP	GO:00725C	divalent	44/1016	499/18862	0.000927	0.025343	0.020883
GO:00303З	BP	GO:00303З	negative	32/1016	330/18862	0.000971	0.026288	0.021662
GO:19030З	BP	GO:19030З	regulatic	32/1016	330/18862	0.000971	0.026288	0.021662
GO:00316€	BP	GO:00316€	cellular	23/1016	210/18862	0.000983	0.026318	0.021687
GO:007037	BP	GO:007037	positive	23/1016	210/18862	0.000983	0.026318	0.021687
GO:000164	BP	GO:000164	osteoblas	24/1016	223/18862	0.000987	0.026318	0.021687
GO:001624	BP	GO:001624	regulatic	19/1016	160/18862	0.00101	0.026744	0.022037
GO:00723З	BP	GO:00723З	signal tr	27/1016	263/18862	0.001015	0.026744	0.022037
GO:19037C	BP	GO:19037C	regulatic	38/1016	415/18862	0.001017	0.026744	0.022037
GO:200014	BP	GO:200014	negative	33/1016	345/18862	0.001029	0.026838	0.022115
GO:00455Ç	BP	GO:00455Ç	regulatic	17/1016	136/18862	0.001031	0.026838	0.022115
GO:004662	BP	GO:004662	regulatic	11/1016	69/18862	0.001082	0.028039	0.023105
GO:00355€	BP	GO:00355€	regulatic	6/1016	23/18862	0.001101	0.028385	0.02339
GO:00466З	BP	GO:00466З	alpha-bet	18/1016	149/18862	0.001111	0.028512	0.023495
GO:00457З	BP	GO:00457З	positive	24/1016	225/18862	0.001117	0.028533	0.023512
GO:00304З	BP	GO:00304З	ubiquitin	12/1016	80/18862	0.001137	0.028906	0.02382
GO:00603З	BP	GO:00603З	interferc	13/1016	91/18862	0.001147	0.029016	0.02391
GO:19021C	BP	GO:19021C	regulatic	28/1016	279/18862	0.001171	0.029415	0.024238

GO:003461	BP	GO:003461	response	31/1016	320/18862	0.001174	0.029415	0.024238
GO:003072	BP	GO:003072	ovulation	5/1016	16/18862	0.001191	0.029415	0.024238
GO:005123	BP	GO:005123	sequester	5/1016	16/18862	0.001191	0.029415	0.024238
GO:004353	BP	GO:004353	regulatic	18/1016	150/18862	0.0012	0.029415	0.024238
GO:005077	BP	GO:005077	negative	18/1016	150/18862	0.0012	0.029415	0.024238
GO:190336	BP	GO:190336	positive	18/1016	150/18862	0.0012	0.029415	0.024238
GO:000918	BP	GO:000918	ribonucle	17/1016	138/18862	0.001214	0.029556	0.024355
GO:004561	BP	GO:004561	regulatic	20/1016	175/18862	0.001217	0.029556	0.024355
GO:000690	BP	GO:000690	vesicle c	11/1016	70/18862	0.001223	0.029556	0.024355
GO:007049	BP	GO:007049	interleuk	14/1016	103/18862	0.001237	0.029774	0.024535
GO:000756	BP	GO:000756	embryo in	9/1016	50/18862	0.001253	0.029901	0.024639
GO:004826	BP	GO:004826	positive	9/1016	50/18862	0.001253	0.029901	0.024639
GO:005116	BP	GO:005116	nuclear e	22/1016	201/18862	0.001263	0.029916	0.024652
GO:004336	BP	GO:004336	CD4-posit	12/1016	81/18862	0.001271	0.029916	0.024652
GO:004346	BP	GO:004346	regulatic	12/1016	81/18862	0.001271	0.029916	0.024652
GO:000836	BP	GO:000836	regulatic	18/1016	151/18862	0.001296	0.030381	0.025035
GO:003300	BP	GO:003300	regulatic	8/1016	41/18862	0.001345	0.031081	0.025611
GO:004208	BP	GO:004208	T-helper	8/1016	41/18862	0.001345	0.031081	0.025611
GO:004564	BP	GO:004564	negative	4/1016	10/18862	0.001353	0.031081	0.025611
GO:004819	BP	GO:004819	Golgi ves	4/1016	10/18862	0.001353	0.031081	0.025611
GO:003003	BP	GO:003003	contracti	14/1016	104/18862	0.00136	0.031081	0.025611
GO:004314	BP	GO:004314	stress fi	14/1016	104/18862	0.00136	0.031081	0.025611
GO:004671	BP	GO:004671	viral ent	17/1016	140/18862	0.001424	0.032289	0.026607
GO:007167	BP	GO:007167	mononucle	21/1016	190/18862	0.001425	0.032289	0.026607
GO:000726	BP	GO:000726	Ras prote	32/1016	338/18862	0.001439	0.032463	0.02675
GO:004327	BP	GO:004327	apoptotic	9/1016	51/18862	0.001451	0.032592	0.026857
GO:005105	BP	GO:005105	negative	39/1016	438/18862	0.001476	0.033029	0.027216
GO:001623	BP	GO:001623	macroautc	30/1016	311/18862	0.001494	0.033287	0.027429
GO:004440	BP	GO:004440	entry int	18/1016	153/18862	0.001507	0.033356	0.027487
GO:001605	BP	GO:001605	vesicle c	32/1016	339/18862	0.001509	0.033356	0.027487
GO:000257	BP	GO:000257	myeloid l	22/1016	204/18862	0.00153	0.033547	0.027643
GO:004470	BP	GO:004470	multi-mul	22/1016	204/18862	0.00153	0.033547	0.027643
GO:004298	BP	GO:004298	amyloid p	13/1016	94/18862	0.001553	0.033912	0.027945
GO:000691	BP	GO:000691	nucleocyt	32/1016	340/18862	0.001583	0.03418	0.028165
GO:004678	BP	GO:004678	regulatic	8/1016	42/18862	0.001584	0.03418	0.028165
GO:007136	BP	GO:007136	cellular	8/1016	42/18862	0.001584	0.03418	0.028165
GO:009752	BP	GO:009752	myeloid l	23/1016	218/18862	0.001621	0.034831	0.028702
GO:004434	BP	GO:004434	cellular	17/1016	142/18862	0.001664	0.03561	0.029344
GO:003278	BP	GO:003278	regulatic	9/1016	52/18862	0.001672	0.03564	0.029369
GO:003021	BP	GO:003021	T cell di	25/1016	246/18862	0.001761	0.037401	0.03082
GO:005116	BP	GO:005116	nuclear t	32/1016	343/18862	0.001822	0.038501	0.031726
GO:000635	BP	GO:000635	DNA-templ	15/1016	119/18862	0.001827	0.038501	0.031726
GO:003447	BP	GO:003447	ncRNA prc	36/1016	400/18862	0.001835	0.038508	0.031731
GO:000166	BP	GO:000166	ameboidal	41/1016	473/18862	0.001873	0.039023	0.032156
GO:000913	BP	GO:000913	nucleosid	18/1016	156/18862	0.001876	0.039023	0.032156
GO:004819	BP	GO:004819	Golgi ves	34/1016	372/18862	0.001881	0.039023	0.032156
GO:003009	BP	GO:003009	lymphocyt	33/1016	358/18862	0.001897	0.03921	0.03231
GO:000269	BP	GO:000269	positive	36/1016	401/18862	0.001915	0.039246	0.03234
GO:200035	BP	GO:200035	negative	7/1016	34/18862	0.001927	0.039246	0.03234
GO:000974	BP	GO:000974	response	23/1016	221/18862	0.001938	0.039246	0.03234
GO:000648	BP	GO:000648	protein N	11/1016	74/18862	0.001942	0.039246	0.03234
GO:003241	BP	GO:003241	lysosome	11/1016	74/18862	0.001942	0.039246	0.03234
GO:004819	BP	GO:004819	vesicle t	11/1016	74/18862	0.001942	0.039246	0.03234
GO:003460	BP	GO:003460	cellular	15/1016	120/18862	0.001987	0.039996	0.032958

GO:00316€	BP	GO:00316€	cellular	24/1016	235/18862	0.002016	0.04044	0.033324
GO:000635	BP	GO:000635	DNA-templ	25/1016	249/18862	0.002079	0.041178	0.033932
GO:004887	BP	GO:004887	homeostas	25/1016	249/18862	0.002079	0.041178	0.033932
GO:00075€	BP	GO:00075€	aging	29/1016	304/18862	0.002096	0.041178	0.033932
GO:00420S	BP	GO:00420S	T-helper	10/1016	64/18862	0.002103	0.041178	0.033932
GO:007257	BP	GO:007257	endotheli	10/1016	64/18862	0.002103	0.041178	0.033932
GO:003297	BP	GO:003297	regulatic	35/1016	389/18862	0.002117	0.041178	0.033932
GO:003134	BP	GO:003134	negative	24/1016	236/18862	0.002133	0.041178	0.033932
GO:00450€	BP	GO:00450€	T-helper	5/1016	18/18862	0.002135	0.041178	0.033932
GO:004611	BP	GO:004611	nucleobas	5/1016	18/18862	0.002135	0.041178	0.033932
GO:190223	BP	GO:190223	negative	5/1016	18/18862	0.002135	0.041178	0.033932
GO:00507€	BP	GO:00507€	negative	16/1016	133/18862	0.002137	0.041178	0.033932
GO:005507	BP	GO:005507	iron ion	12/1016	86/18862	0.002149	0.041257	0.033997
GO:004327	BP	GO:004327	negative	30/1016	319/18862	0.002205	0.042188	0.034764
GO:190332	BP	GO:190332	regulatic	24/1016	237/18862	0.002256	0.043016	0.035446
GO:005181	BP	GO:005181	modulatic	13/1016	98/18862	0.002274	0.043059	0.035482
GO:011002	BP	GO:011002	regulatic	13/1016	98/18862	0.002274	0.043059	0.035482
GO:00107€	BP	GO:00107€	positive	7/1016	35/18862	0.002299	0.043076	0.035496
GO:001624	BP	GO:001624	negative	7/1016	35/18862	0.002299	0.043076	0.035496
GO:19058S	BP	GO:19058S	positive	7/1016	35/18862	0.002299	0.043076	0.035496
GO:19033€	BP	GO:19033€	regulatic	25/1016	251/18862	0.002317	0.043269	0.035655
GO:005134	BP	GO:005134	regulatic	14/1016	110/18862	0.002334	0.043412	0.035773
GO:004603	BP	GO:004603	ADP metak	15/1016	122/18862	0.002341	0.043412	0.035773
GO:004582	BP	GO:004582	negative	10/1016	65/18862	0.002367	0.043751	0.036052
GO:004232	BP	GO:004232	negative	36/1016	407/18862	0.002459	0.045296	0.037325
GO:00326{	BP	GO:00326{	regulatic	18/1016	160/18862	0.002486	0.045537	0.037524
GO:005087	BP	GO:005087	positive	22/1016	212/18862	0.002489	0.045537	0.037524
GO:19034C	BP	GO:19034C	reactive	15/1016	123/18862	0.002537	0.04625	0.038111
GO:007177	BP	GO:007177	response	17/1016	148/18862	0.002596	0.047175	0.038873
GO:00022S	BP	GO:00022S	CD4-posit	10/1016	66/18862	0.002658	0.047723	0.039325
GO:00456C	BP	GO:00456C	positive	10/1016	66/18862	0.002658	0.047723	0.039325
GO:190007	BP	GO:190007	regulatic	10/1016	66/18862	0.002658	0.047723	0.039325
GO:00094C	BP	GO:00094C	response	18/1016	161/18862	0.002662	0.047723	0.039325
GO:00324{	BP	GO:00324{	positive	11/1016	77/18862	0.002682	0.04782	0.039405
GO:000282	BP	GO:000282	regulatic	6/1016	27/18862	0.002685	0.04782	0.039405
GO:000283	BP	GO:000283	negative	13/1016	100/18862	0.002727	0.048249	0.039759
GO:003571	BP	GO:003571	CD4-posit	13/1016	100/18862	0.002727	0.048249	0.039759
GO:003475	BP	GO:003475	iron ion	5/1016	19/18862	0.002771	0.048709	0.040137
GO:006097	BP	GO:006097	left/righ	5/1016	19/18862	0.002771	0.048709	0.040137
GO:007135	BP	GO:007135	cellular	28/1016	296/18862	0.002795	0.048974	0.040356
GO:00108{	BP	GO:00108{	regulatic	9/1016	56/18862	0.002841	0.049322	0.040643
GO:006013	BP	GO:006013	maternal	9/1016	56/18862	0.002841	0.049322	0.040643
GO:003264	BP	GO:003264	tumor nec	18/1016	162/18862	0.002848	0.049322	0.040643
GO:004353	BP	GO:004353	blood ves	19/1016	175/18862	0.002873	0.049322	0.040643
GO:00457€	BP	GO:00457€	positive	19/1016	175/18862	0.002873	0.049322	0.040643
GO:190401	BP	GO:190401	positive	19/1016	175/18862	0.002873	0.049322	0.040643
GO:00514S	BP	GO:00514S	regulatic	12/1016	89/18862	0.002879	0.049322	0.040643
GO:00341S	BP	GO:00341S	cellular	8/1016	46/18862	0.002896	0.049322	0.040643
GO:00433C	BP	GO:00433C	mast cell	8/1016	46/18862	0.002896	0.049322	0.040643
GO:00420€	BP	GO:00420€	gliogenes	27/1016	283/18862	0.002924	0.049323	0.040644
GO:003433	BP	GO:003433	adherens	4/1016	12/18862	0.002924	0.049323	0.040644
GO:003811	BP	GO:003811	interleuk	4/1016	12/18862	0.002924	0.049323	0.040644
GO:00602{	BP	GO:00602{	regulatic	41/1016	485/18862	0.002947	0.049556	0.040835
GO:00022{	BP	GO:00022{	alpha-bet	10/1016	67/18862	0.002977	0.049632	0.040898

GO:000229	BP	GO:000229	alpha-bet	10/1016	67/18862	0.002977	0.049632	0.040898
GO:004562	BP	GO:004562	positive	13/1016	101/18862	0.002979	0.049632	0.040898
GO:004583	BP	GO:004583	positive	17/1016	150/18862	0.00299	0.049669	0.040929
GO:005086	BP	GO:005086	positive	36/1016	412/18862	0.003011	0.049853	0.04108
GO:000592	CC	GO:000592	focal adh	46/1037	416/19520	2.12E-06	0.00075	0.000649
GO:003005	CC	GO:003005	cell-subs	46/1037	423/19520	3.33E-06	0.00075	0.000649
GO:009057	CC	GO:009057	RNA polym	25/1037	170/19520	3.66E-06	0.00075	0.000649
GO:003068	CC	GO:003068	preribosc	15/1037	78/19520	1.28E-05	0.00197	0.001703
GO:01010C	CC	GO:01010C	ficolin-1	24/1037	185/19520	4.77E-05	0.005861	0.005066
GO:003125	CC	GO:003125	cell lead	41/1037	411/19520	8.24E-05	0.008427	0.007284
GO:000579	CC	GO:000579	Golgi-ass	15/1037	92/19520	9.59E-05	0.008427	0.007284
GO:000576	CC	GO:000576	lysosomal	38/1037	378/19520	0.000126	0.008621	0.007452
GO:009885	CC	GO:009885	lytic vac	38/1037	378/19520	0.000126	0.008621	0.007452
GO:000577	CC	GO:000577	vacuolar	41/1037	431/19520	0.00023	0.014119	0.012204
GO:00166C	CC	GO:00166C	nuclear s	39/1037	411/19520	0.000339	0.018976	0.016402
GO:000566	CC	GO:000566	transcrip	38/1037	409/19520	0.000603	0.0289	0.02498
GO:003068	CC	GO:003068	90S preri	7/1037	29/19520	0.000652	0.0289	0.02498
GO:000577	CC	GO:000577	autophagc	14/1037	98/19520	0.000658	0.0289	0.02498
GO:003204	CC	GO:003204	small-sub	8/1037	39/19520	0.000871	0.033724	0.029149
GO:190481	CC	GO:190481	ficolin-1	16/1037	124/19520	0.000881	0.033724	0.029149
GO:000563	CC	GO:000563	nuclear e	41/1037	462/19520	0.000932	0.033724	0.029149
GO:004512	CC	GO:004512	membrane	31/1037	323/19520	0.0011	0.03559	0.030762
GO:009885	CC	GO:009885	membrane	31/1037	323/19520	0.0011	0.03559	0.030762
GO:007082	CC	GO:007082	tertiary	19/1037	164/19520	0.001158	0.035611	0.030781
GO:000591	CC	GO:000591	cell-cell	42/1037	485/19520	0.001309	0.038335	0.033135
GO:003051	MF	GO:003051	snoRNA bi	11/1024	32/18337	6.80E-07	0.000339	0.000301
GO:000371	MF	GO:000371	transcrip	54/1024	480/18337	7.46E-07	0.000339	0.000301
GO:014029	MF	GO:014029	DNA-bindi	45/1024	376/18337	1.18E-06	0.000357	0.000317
GO:000371	MF	GO:000371	transcrip	33/1024	258/18337	7.65E-06	0.001414	0.001258
GO:006162	MF	GO:006162	RNA polym	34/1024	271/18337	8.54E-06	0.001414	0.001258
GO:004529	MF	GO:004529	cadherin	39/1024	332/18337	9.35E-06	0.001414	0.001258
GO:003525	MF	GO:003525	nuclear h	20/1024	140/18337	0.000101	0.013084	0.011634
GO:00199C	MF	GO:00199C	phosphata	24/1024	193/18337	0.000199	0.022546	0.020047
GO:005111	MF	GO:005111	ATPase bi	14/1024	85/18337	0.000243	0.023838	0.021196
GO:004432	MF	GO:004432	ion chann	18/1024	128/18337	0.000269	0.023838	0.021196
GO:003525	MF	GO:003525	glucocort	5/1024	12/18337	0.000306	0.023838	0.021196
GO:000188	MF	GO:000188	nucleosid	39/1024	390/18337	0.000315	0.023838	0.021196
GO:000028	MF	GO:000028	magnesium	25/1024	216/18337	0.000449	0.029395	0.026137
GO:000188	MF	GO:000188	purine nu	38/1024	384/18337	0.000459	0.029395	0.026137
GO:000371	MF	GO:000371	transcrip	22/1024	182/18337	0.000534	0.029395	0.026137
GO:004687	MF	GO:004687	ephrin re	7/1024	27/18337	0.000549	0.029395	0.026137
GO:000392	MF	GO:000392	GTPase ac	33/1024	322/18337	0.000577	0.029395	0.026137
GO:00199C	MF	GO:00199C	protein p	19/1024	148/18337	0.000595	0.029395	0.026137
GO:000178	MF	GO:000178	phosphoty	9/1024	44/18337	0.000615	0.029395	0.026137
GO:003037	MF	GO:003037	nuclear r	10/1024	54/18337	0.000715	0.032443	0.028847
GO:003255	MF	GO:003255	purine ri	37/1024	381/18337	0.000769	0.033262	0.029576
GO:005178	MF	GO:005178	misfolded	7/1024	29/18337	0.000876	0.035076	0.031189
GO:003254	MF	GO:003254	ribonucle	37/1024	384/18337	0.000888	0.035076	0.031189
GO:001692	MF	GO:001692	nuclear r	14/1024	97/18337	0.000961	0.036369	0.032339
GO:003525	MF	GO:003525	steroid h	12/1024	77/18337	0.001099	0.039912	0.035489
GO:000552	MF	GO:000552	GTP bindi	36/1024	376/18337	0.001159	0.04048	0.035993
GO:005172	MF	GO:005172	protein p	7/1024	31/18337	0.001338	0.045006	0.040018
GO:010631	MF	GO:010631	NA	26/1024	248/18337	0.001529	0.046392	0.041251
GO:010631	MF	GO:010631	NA	26/1024	249/18337	0.00162	0.046392	0.041251

GO:005142	MF	GO:005142	hormone r	20/1024	173/18337	0.001624	0.046392	0.041251
GO:003149	MF	GO:003149	nucleosom	11/1024	70/18337	0.001631	0.046392	0.041251
GO:001900	MF	GO:001900	guanyl nu	37/1024	398/18337	0.001686	0.046392	0.041251
GO:003256	MF	GO:003256	guanyl ri	37/1024	398/18337	0.001686	0.046392	0.041251

geneID	Count
NRBF2/CAS	41
SH3RF1/AA	44
SIK2/SOCS	42
SHC1/THBS	31
SHC1/SDF2	27
AP3D1/SLC	12
SH3RF1/AA	32
SIK2/RIPK	46
SHC1/THBS	28
AP3D1/SLC	12
PIKFYVE/I	42
RIPK2/NOD	50
DAXX/MT1H	10
AP3D1/HAM	21
SHC1/DAXX	24
AP3D1/HAM	23
EIF2AK3/A	9
RIPK2/NOD	44
MID1/RIPK	35
DAXX/AKT1	11
NPC1/DAXX	14
AATF/NOLC	37
BCL3/CASP	35
SHC1/EIF2	21
IL1RAP/RI	26
SIK2/SOCS	47
IFIT1/IFI	17
MID1/RIPK	33
RIPK2/VIT	46
NUPR1/EIF	16
MT1H/MT1F	7
MT1H/MT1F	7
IL1RAP/RI	28
EIF2AK3/A	9
DAXX/MT1H	11
RIPK2/HNR	12
MIR99A/SO	12
AKT1/PTPN	17
RIPK2/IL4	33
MT1H/MT1F	7
NUPR1/AKT	23
EIF2AK3/A	8
AGTRAP/AG	8
AATF/NOLC	48
PIKFYVE/I	28
BCL3/DIAP	36
MT1H/MT1F	8
RIPK2/RAL	12
MT1H/MT1F	7
MIR99A/SO	11
SIK2/SOCS	21
SUCO/SMAD	42
AGTRAP/AG	8

HBB/MVP/D	48
HBB/MVP/D	49
STK3/THBS	15
GAB2/KLF1	43
TNFRSF11B	41
RIPK2/BCL	47
HBB/MVP/D	48
TNFRSF11B	41
EIF2AK3/A	9
IL1RAP/RI	31
HNRNPF/SI	11
TNFRSF11B	41
RIPK2/RAL	38
IL1RAP/RI	44
RIPK2/NOD	35
MID1/SOCS	25
IFIT1/IFI	19
IFIT1/IFI	24
RIPK2/IL4	28
TNFRSF11B	11
GAB2/SPRY	43
HBB/MVP/D	48
RIPK2/TNI	35
CASP4/EIF	12
SMAD3/SOC	38
RIPK2/NOD	33
SIK2/SOCS	27
PTPN1/NR1	10
AATF/B4GA	42
RIPK2/NOD	35
EIF2AK3/A	11
NOLC1/WDR	27
SMAD3/CSN	39
TNFRSF11B	44
HBB/PIKFY	24
NPLOC4/UB	16
NPLOC4/UB	8
MCAM/SERP	8
NPLOC4/UB	8
OSBPL8/NR	7
SIK2/RALB	21
RIPK2/BCL	41
SMAD3/SH3	26
SOCS3/PTP	9
IFIT1/IFI	14
RIPK2/NOD	31
IFIT1/IFI	20
EIF2AK3/P	8
SIK2/RALB	24
IL1RAP/RI	43
IFIT1/IFI	24
NOD1/POR/	11
OSBPL8/PL	14
RIPK2/IL4	37

SOCS3/PTF	9
PIKFYVE/F	22
ANGPT2/SM	39
RIPK2/NOD	37
TNFRSF11E	45
SIK2/UPP1	10
IFITM1/PT	7
RIPK2/IL4	42
NOLC1/WDF	27
THBS1/OSE	12
ARF3/TMED	14
SIK2/PIKF	26
ANGPT2/JU	22
MID1/SOCS	22
SIK2/SOCS	30
RIPK2/NOD	17
EIF2AK3/M	12
RIPK2/TNI	35
HBB/SMAD3	11
STK3/THBS	11
SMAD3/ATX	30
RIPK2/NOD	22
SMAD3/STK	27
GAB2/IL4R	6
IL1RAP/RI	20
HBB/SMAD3	11
MID1/RIPK	22
GAB2/IL4R	5
GAB2/IL4R	5
RIPK2/TNI	23
RIPK2/NOD	29
SMAD3/DIA	44
MIR30E/AN	34
HBB/STXBF	11
MVP/ZFP36	23
MVP/ZFP36	23
KLF13/ZFF	16
RTN4/BCL1	21
CSNK1D/TM	44
TNFRSF11E	9
NRBF2/TAF	22
HBB/SMAD3	12
RIPK2/OTU	17
MVP/ZFP36	23
IFIT1/IFI	10
IFI16/ZFF	8
TMF1/PREL	22
MVP/ZFP36	27
JUNB/STC1	7
RIPK2/OTU	17
AATF/B4GA	34
HBB/STXBF	13
STAT5B/PT	5
RIPK2/BCL	15

MIR30E/AN	33
MAP3K3/TH	12
RIPK2/IL4	19
RIPK2/IL4	19
ANGPT2/CY	36
MCAM/SERF	6
TNFRSF11E	13
PDE8A/ZFF	9
DGKD/RAB3	14
RIPK2/NOD	26
TNFRSF11E	34
ANGPT2/CY	34
MID1/RIPK	21
SMAD3/NUF	31
KLF13/ZFF	16
PIKFYVE/T	36
GAB2/IL4R	9
HNRNPF/MI	9
KLF13/ZFF	9
HBB/SMAD3	12
KLF13/HAM	18
RIPK2/TNI	25
HBB/PIKFY	14
SHC1/PTPN	11
HAMP/MT1H	10
SMAD3/ATX	30
TRIM8/TRI	5
CSNK1D/SF	5
RIPK2/NOD	23
SMAD3/STK	24
MVP/ZFP36	25
XPO6/SDAD	21
HBB/SMAD3	12
RIPK2/TNI	23
NPLOC4/UE	9
RIPK2/RAL	9
SMAD3/DIA	44
ANGPT2/CY	32
RIPK2/IL4	32
SIK2/RALE	23
RIPK2/NOD	23
SUCO/SMAD	24
RIPK2/NOD	19
RPA1/BCL3	27
RIPK2/SH3	38
ANGPT2/CY	33
SMAD3/STK	17
SIK2/SOCS	11
EBF2/HMBC	6
RIPK2/BCL	18
CSNK1D/RN	24
NPLOC4/UE	12
MID1/SOCS	13
RIPK2/SH3	28

TNFRSF11E	31
IL4R/NRIF	5
AP3D1/SLC	5
ANGPT2/MA	18
IL4R/IFI1	18
CSNK1D/RN	18
NUPR1/INS	17
RIPK2/SH3	20
CSNK1D/TM	11
IL1RAP/RI	14
STC1/UBE2	9
DGKD/INSR	9
XPO6/SDAD	22
RIPK2/BCL	12
IFIT1/CNN	12
DIAPH1/CD	18
GAB2/IL4R	8
RIPK2/BCL	8
KLF13/ZFF	4
TMED9/ARF	4
SMAD3/TNF	14
SMAD3/TNF	14
PIKFYVE/F	17
THBS1/ADA	21
DENND4A/R	32
THBS1/TGM	9
MIDN/RRAD	39
RIPK2/PIK	30
PIKFYVE/F	18
PIKFYVE/C	32
GAB2/JUNE	22
ANGPT2/JU	22
AATF/RTN4	13
SMAD3/NOL	32
TRIM8/TRI	8
PDE8A/ZFF	8
PIKFYVE/T	23
SPRY1/THE	17
HMGN1/NEL	9
RIPK2/BCL	25
SMAD3/NOL	32
HMGN1/SSR	15
PUS7/SMAD	36
ANGPT2/CY	41
NUPR1/INS	18
CSNK1D/AF	34
RIPK2/BCL	33
GAB2/RIPK	36
MIR30E/TN	7
ANGPT2/TH	23
TMEM165/F	11
GAB2/IL4R	11
CSNK1D/TM	11
RPA1/THBS	15

SIK2/RALE	24
NRBF2/TAF	25
KLF13/VPS	25
MAP3K3/CA	29
RIPK2/BCL	10
MIR30E/TN	10
SMAD3/STC	35
SMAD3/SOC	24
RIPK2/IL4	5
CTPS1/CMF	5
PTPN1/GRI	5
RTN4/HES1	16
HAMP/STEA	12
MIDN/RRAD	30
RIPK2/CDC	24
STOM/PTX3	13
SMAD3/ACT	13
AKT1/AHCY	7
NUPR1/NPC	7
PTPN1/TME	7
CSNK1D/TM	25
NOD1/POR/	14
NUPR1/INS	15
IFI16/NR1	10
MVP/SOCS3	36
RIPK2/NOD	18
RIPK2/IL4	22
HBB/PIKFY	15
SPRY1/THE	17
RIPK2/BCL	10
STK3/CARM	10
SOCS3/PTF	10
RPA1/THBS	18
RIPK2/IFI	11
RIPK2/IL4	6
IFI16/NPL	13
RIPK2/BCL	13
HAMP/SLC3	5
IFT57/MEG	5
TNFRSF11E	28
OSBPL8/PL	9
ANGPT2/JU	9
RIPK2/NOD	18
ANGPT2/MA	19
ANGPT2/CY	19
ANGPT2/CY	19
SMAD3/ACT	12
EIF2AK3/M	8
GAB2/IL4R	8
RTN4/B4GA	27
HIPK1/CTN	4
SHC1/STAT	4
RTN4/BCL1	41
RIPK2/BCL	10

RIPK2/BCL	10
RIPK2/IL4	13
MID1IP1/A	17
GAB2/RIPK	36
CNN3/ACTN	46
CNN3/ACTN	46
SMAD3/TAF	25
WDR3/NOP1	15
HBB/MVP/D	24
GABARAPL1	41
PACS1/NCA	15
SLC7A5/AF	38
SLC7A5/AF	38
SLC7A5/GA	41
AAGAB/IFI	39
SMAD3/AAT	38
WDR3/NOP1	7
GABARAPL1	14
WDR3/NOP1	8
HBB/MVP/I	16
MVP/SMAD3	41
PIKFYVE/F	31
PIKFYVE/F	31
HBB/DIAPH	19
PIKFYVE/C	42
NOLC1/WDR	11
BCOR/TMF1	54
GABARAPL1	45
TMF1/ACTN	33
GABARAPL1	34
CNN3/CSNK	39
SMAD3/TAF	20
MVP/SMAD3	24
RALB/ANK1	14
PACS1/DIA	18
SMAD3/YWH	5
RALB/MPPE	39
SIK2/NT5C	25
RALB/MPPE	38
BCOR/DAXX	22
SHC1/EFNA	7
RALB/RRAD	33
MVP/ANK1/	19
SOCS3/SHC	9
TMF1/ACTN	10
RALB/MPPE	37
SDF2L1/RH	7
RALB/MPPE	37
SMAD3/TAF	14
SMAD3/TAF	12
RALB/RRAD	36
AKT1/ANKL	7
SIK2/RIPK	26
SIK2/RIPK	26

SMAD3/TAF	20
HMGN1/SMA	11
RALB/MPPE	37
RALB/MPPE	37

	ONTOLOGY	ID	Descripti	GeneRatic	BgRatio	pvalue	p.adjust	qvalue
GO:003254	BP	GO:003254	mitochond	31/312	134/18862	7.13E-27	1.02E-23	9.52E-24
GO:004533	BP	GO:004533	cellular	35/312	187/18862	7.47E-27	1.02E-23	9.52E-24
GO:014005	BP	GO:014005	mitochond	32/312	165/18862	3.90E-25	3.56E-22	3.32E-22
GO:007012	BP	GO:007012	mitochond	24/312	88/18862	6.59E-23	4.50E-20	4.20E-20
GO:003310	BP	GO:003310	mitochond	25/312	101/18862	1.16E-22	6.32E-20	5.89E-20
GO:007012	BP	GO:007012	mitochond	23/312	89/18862	2.07E-21	9.45E-19	8.80E-19
GO:001598	BP	GO:001598	energy de	35/312	278/18862	6.79E-21	2.65E-18	2.47E-18
GO:000641	BP	GO:000641	translati	26/312	134/18862	1.27E-20	4.35E-18	4.05E-18
GO:000641	BP	GO:000641	translati	23/312	105/18862	1.23E-19	3.73E-17	3.47E-17
GO:004277	BP	GO:004277	mitochond	21/312	99/18862	1.00E-17	2.74E-15	2.55E-15
GO:004277	BP	GO:004277	ATP synth	21/312	100/18862	1.25E-17	2.74E-15	2.55E-15
GO:001025	BP	GO:001025	NADH dehy	18/312	65/18862	1.30E-17	2.74E-15	2.55E-15
GO:003298	BP	GO:003298	mitochond	18/312	65/18862	1.30E-17	2.74E-15	2.55E-15
GO:002290	BP	GO:002290	electron	26/312	178/18862	2.10E-17	4.10E-15	3.82E-15
GO:002290	BP	GO:002290	respiratc	21/312	117/18862	3.72E-16	6.61E-14	6.15E-14
GO:000611	BP	GO:000611	oxidative	23/312	148/18862	3.86E-16	6.61E-14	6.15E-14
GO:000906	BP	GO:000906	aerobic r	18/312	86/18862	2.91E-15	4.69E-13	4.37E-13
GO:000612	BP	GO:000612	mitochond	15/312	55/18862	8.90E-15	1.35E-12	1.26E-12
GO:000609	BP	GO:000609	tricarbox	12/312	34/18862	1.35E-13	1.94E-11	1.81E-11
GO:004362	BP	GO:004362	cellular	24/312	221/18862	3.16E-13	4.32E-11	4.03E-11
GO:004603	BP	GO:004603	ATP metab	25/312	313/18862	9.11E-11	1.19E-08	1.11E-08
GO:000683	BP	GO:000683	mitochond	23/312	274/18862	1.98E-10	2.46E-08	2.29E-08
GO:003298	BP	GO:003298	protein-c	25/312	328/18862	2.46E-10	2.93E-08	2.73E-08
GO:000608	BP	GO:000608	acetyl-Cc	9/312	38/18862	8.84E-09	1.01E-06	9.38E-07
GO:000663	BP	GO:000663	acyl-CoA	13/312	105/18862	1.93E-08	2.03E-06	1.89E-06
GO:003538	BP	GO:003538	thioester	13/312	105/18862	1.93E-08	2.03E-06	1.89E-06
GO:003361	BP	GO:003361	mitochond	7/312	22/18862	4.37E-08	4.42E-06	4.12E-06
GO:003386	BP	GO:003386	nucleosid	14/312	140/18862	8.60E-08	7.85E-06	7.31E-06
GO:003387	BP	GO:003387	ribonucle	14/312	140/18862	8.60E-08	7.85E-06	7.31E-06
GO:003403	BP	GO:003403	purine nu	14/312	140/18862	8.60E-08	7.85E-06	7.31E-06
GO:007265	BP	GO:007265	establish	14/312	142/18862	1.03E-07	8.86E-06	8.25E-06
GO:001700	BP	GO:001700	cytochrom	8/312	36/18862	1.04E-07	8.86E-06	8.25E-06
GO:007058	BP	GO:007058	protein l	14/312	146/18862	1.46E-07	1.21E-05	1.13E-05
GO:000853	BP	GO:000853	respiratc	7/312	26/18862	1.59E-07	1.28E-05	1.19E-05
GO:000660	BP	GO:000660	protein t	24/312	441/18862	3.67E-07	2.81E-05	2.62E-05
GO:000679	BP	GO:000679	sulfur cc	22/312	378/18862	3.69E-07	2.81E-05	2.62E-05
GO:000662	BP	GO:000662	protein t	11/312	100/18862	8.14E-07	5.89E-05	5.49E-05
GO:000700	BP	GO:000700	mitochond	13/312	144/18862	8.18E-07	5.89E-05	5.49E-05
GO:000608	BP	GO:000608	acetyl-Cc	6/312	22/18862	1.17E-06	8.17E-05	7.61E-05
GO:199054	BP	GO:199054	mitochond	11/312	109/18862	1.93E-06	0.000132	0.000123
GO:007012	BP	GO:007012	regulatic	6/312	25/18862	2.65E-06	0.000177	0.000165
GO:004428	BP	GO:004428	small mol	22/312	431/18862	3.25E-06	0.000212	0.000197
GO:001605	BP	GO:001605	organic a	16/312	258/18862	6.70E-06	0.000418	0.00039
GO:006212	BP	GO:006212	regulatic	6/312	29/18862	6.73E-06	0.000418	0.00039
GO:004639	BP	GO:004639	carboxyli	15/312	243/18862	1.37E-05	0.000835	0.000778
GO:003015	BP	GO:003015	protein i	5/312	20/18862	1.52E-05	0.000902	0.00084
GO:004474	BP	GO:004474	protein t	6/312	36/18862	2.50E-05	0.00142	0.001323
GO:003538	BP	GO:003538	thioester	7/312	53/18862	2.54E-05	0.00142	0.001323
GO:007161	BP	GO:007161	acyl-CoA	7/312	53/18862	2.54E-05	0.00142	0.001323
GO:004276	BP	GO:004276	DNA damag	6/312	39/18862	4.02E-05	0.002201	0.00205
GO:000685	BP	GO:000685	mitochond	5/312	25/18862	4.85E-05	0.002604	0.002426
GO:007180	BP	GO:007180	protein t	7/312	60/18862	5.77E-05	0.003037	0.002829
GO:000608	BP	GO:000608	acetyl-Cc	4/312	15/18862	8.68E-05	0.004478	0.004171

GO:007252	BP	GO:007252	purine-cc	20/312	460/18862	8.84E-05	0.004478	0.004171
GO:000925	BP	GO:000925	ribonucle	19/312	425/18862	9.22E-05	0.004586	0.004272
GO:003386	BP	GO:003386	nucleosid	7/312	67/18862	0.000118	0.005555	0.005175
GO:003403	BP	GO:003403	ribonucle	7/312	67/18862	0.000118	0.005555	0.005175
GO:003403	BP	GO:003403	purine nu	7/312	67/18862	0.000118	0.005555	0.005175
GO:001969	BP	GO:001969	ribose ph	19/312	435/18862	0.000125	0.005787	0.00539
GO:000915	BP	GO:000915	purine ri	18/312	408/18862	0.000166	0.00756	0.007042
GO:006500	BP	GO:006500	intracell	6/312	51/18862	0.000188	0.00843	0.007852
GO:000639	BP	GO:000639	tRNA meta	11/312	179/18862	0.000201	0.008857	0.00825
GO:009015	BP	GO:009015	establish	6/312	53/18862	0.000233	0.010122	0.009428
GO:000663	BP	GO:000663	fatty aci	7/312	75/18862	0.00024	0.010267	0.009563
GO:005120	BP	GO:005120	protein i	7/312	77/18862	0.000283	0.011909	0.011092
GO:000908	BP	GO:000908	branched-	4/312	20/18862	0.000289	0.011963	0.011143
GO:000700	BP	GO:000700	inner mit	6/312	56/18862	0.000316	0.012913	0.012028
GO:001939	BP	GO:001939	fatty aci	8/312	105/18862	0.000356	0.014325	0.013342
GO:000906	BP	GO:000906	fatty aci	8/312	106/18862	0.00038	0.014996	0.013968
GO:004427	BP	GO:004427	sulfur cc	11/312	193/18862	0.000384	0.014996	0.013968
GO:000616	BP	GO:000616	purine nu	18/312	441/18862	0.000424	0.016343	0.015222
GO:000703	BP	GO:000703	peroxisom	7/312	84/18862	0.000483	0.018245	0.016994
GO:003444	BP	GO:003444	lipid oxi	8/312	110/18862	0.000487	0.018245	0.016994
GO:001094	BP	GO:001094	negative	3/312	10/18862	0.000493	0.018245	0.016994
GO:000908	BP	GO:000908	branched-	4/312	23/18862	0.000507	0.018503	0.017235
GO:000640	BP	GO:000640	RNA catab	17/312	414/18862	0.00057	0.02051	0.019104
GO:000608	BP	GO:000608	cellular	6/312	63/18862	0.000601	0.021348	0.019884
GO:004589	BP	GO:004589	positive	3/312	11/18862	0.00067	0.023212	0.021621
GO:190247	BP	GO:190247	positive	3/312	11/18862	0.00067	0.023212	0.021621
GO:000095	BP	GO:000095	nuclear-t	11/312	208/18862	0.000718	0.024559	0.022875
GO:009015	BP	GO:009015	establish	15/312	354/18862	0.000866	0.029268	0.027261
GO:000662	BP	GO:000662	protein t	6/312	68/18862	0.000903	0.029424	0.027407
GO:007266	BP	GO:007266	protein l	6/312	68/18862	0.000903	0.029424	0.027407
GO:007266	BP	GO:007266	establish	6/312	68/18862	0.000903	0.029424	0.027407
GO:000095	BP	GO:000095	mitochond	5/312	47/18862	0.001041	0.033519	0.031221
GO:003644	BP	GO:003644	calcium i	3/312	13/18862	0.001134	0.036063	0.03359
GO:000926	BP	GO:000926	ribonucle	10/312	188/18862	0.001188	0.037361	0.0348
GO:004357	BP	GO:004357	peroxisom	6/312	72/18862	0.00122	0.037945	0.035344
GO:000652	BP	GO:000652	cellular	14/312	331/18862	0.001299	0.039873	0.037139
GO:000628	BP	GO:000628	transcrip	6/312	73/18862	0.001312	0.039873	0.037139
GO:000612	BP	GO:000612	mitochond	3/312	14/18862	0.001425	0.042347	0.039443
GO:004589	BP	GO:004589	regulatic	3/312	14/18862	0.001425	0.042347	0.039443
GO:000641	BP	GO:000641	translati	10/312	193/18862	0.001447	0.042347	0.039443
GO:000803	BP	GO:000803	tRNA proc	8/312	130/18862	0.001455	0.042347	0.039443
GO:007232	BP	GO:007232	monocarbc	8/312	131/18862	0.001528	0.043623	0.040632
GO:000218	BP	GO:000218	cytoplasm	7/312	102/18862	0.001531	0.043623	0.040632
GO:004639	BP	GO:004639	ribose ph	10/312	195/18862	0.001562	0.04406	0.04104
GO:004424	BP	GO:004424	cellular	11/312	230/18862	0.00162	0.044891	0.041814
GO:004507	BP	GO:004507	positive	4/312	31/18862	0.001624	0.044891	0.041814
GO:000575	CC	GO:000575	mitochond	79/320	476/19520	8.13E-57	3.15E-54	2.65E-54
GO:009879	CC	GO:009879	mitochond	60/320	265/19520	3.15E-51	6.09E-49	5.14E-49
GO:000574	CC	GO:000574	mitochond	72/320	493/19520	1.39E-47	1.79E-45	1.51E-45
GO:009880	CC	GO:009880	inner mit	30/320	142/19520	7.00E-25	6.77E-23	5.71E-23
GO:007046	CC	GO:007046	respirasc	23/320	103/19520	6.43E-20	4.98E-18	4.20E-18
GO:199020	CC	GO:199020	oxidoredu	23/320	111/19520	3.93E-19	2.53E-17	2.14E-17
GO:000031	CC	GO:000031	organella	20/320	87/19520	9.56E-18	4.62E-16	3.90E-16
GO:000576	CC	GO:000576	mitochond	20/320	87/19520	9.56E-18	4.62E-16	3.90E-16

GO:000574CC	GO:000574mitochond	20/320	89/19520	1.55E-17	6.66E-16	5.62E-16
GO:004439CC	GO:004439ribosomal	26/320	187/19520	6.04E-17	2.34E-15	1.97E-15
GO:000584CC	GO:000584ribosome	28/320	243/19520	5.45E-16	1.92E-14	1.62E-14
GO:00988CCC	GO:00988Crespiratc	18/320	87/19520	3.14E-15	1.01E-13	8.54E-14
GO:000574CC	GO:000574mitochond	13/320	50/19520	9.97E-13	2.57E-11	2.17E-11
GO:003096CC	GO:003096NADH dehy	13/320	50/19520	9.97E-13	2.57E-11	2.17E-11
GO:004527CC	GO:004527respiratc	13/320	50/19520	9.97E-13	2.57E-11	2.17E-11
GO:001593CC	GO:001593large rib	16/320	115/19520	6.49E-11	1.54E-09	1.30E-09
GO:00313CCC	GO:00313Cintrinsic	12/320	55/19520	7.16E-11	1.54E-09	1.30E-09
GO:00313CCC	GO:00313Cintegral	12/320	55/19520	7.16E-11	1.54E-09	1.30E-09
GO:003259CC	GO:003259integral	14/320	85/19520	1.02E-10	2.07E-09	1.74E-09
GO:000031CC	GO:000031organella	12/320	57/19520	1.12E-10	2.07E-09	1.74E-09
GO:000576CC	GO:000576mitochond	12/320	57/19520	1.12E-10	2.07E-09	1.74E-09
GO:009857CC	GO:009857intrinsic	14/320	86/19520	1.20E-10	2.11E-09	1.78E-09
GO:000031CC	GO:000031organella	8/320	28/19520	1.12E-08	1.80E-07	1.52E-07
GO:000576CC	GO:000576mitochond	8/320	28/19520	1.12E-08	1.80E-07	1.52E-07
GO:001593CC	GO:001593small rib	10/320	74/19520	3.46E-07	5.36E-06	4.52E-06
GO:004523CC	GO:004523tricarbox	5/320	14/19520	2.03E-06	3.03E-05	2.55E-05
GO:00313CCC	GO:00313Cintegral	19/320	371/19520	1.30E-05	0.000187	0.000158
GO:003196CC	GO:003196organelle	14/320	220/19520	1.72E-05	0.000238	0.000201
GO:001986CC	GO:001986outer mem	14/320	222/19520	1.91E-05	0.000254	0.000215
GO:000574CC	GO:000574mitochond	13/320	195/19520	2.11E-05	0.000272	0.000229
GO:009879CC	GO:009879outer mit	5/320	23/19520	3.03E-05	0.000378	0.000319
GO:00313CCC	GO:00313Cintrinsic	19/320	402/19520	3.93E-05	0.000476	0.000401
GO:00014CCC	GO:00014CSAM compl	4/320	13/19520	4.51E-05	0.000513	0.000433
GO:014027CC	GO:014027MIB compl	4/320	13/19520	4.51E-05	0.000513	0.000433
GO:000574CC	GO:000574TIM23 mit	4/320	14/19520	6.23E-05	0.000689	0.000581
GO:000929CC	GO:000929nucleoid	6/320	44/19520	7.74E-05	0.000809	0.000682
GO:004264CC	GO:004264mitochond	6/320	44/19520	7.74E-05	0.000809	0.000682
GO:000577CC	GO:000577peroxisom	9/320	136/19520	0.000414	0.004109	0.003465
GO:004257CC	GO:004257microbody	9/320	136/19520	0.000414	0.004109	0.003465
GO:003197CC	GO:003197organelle	7/320	93/19520	0.000846	0.008188	0.006904
GO:000578CC	GO:000578peroxisom	5/320	51/19520	0.001454	0.013396	0.011295
GO:00319CCC	GO:00319Cmicrobody	5/320	51/19520	0.001454	0.013396	0.011295
GO:000575CC	GO:000575mitochond	6/320	83/19520	0.002427	0.021843	0.018418
GO:007006CC	GO:007006cytochrom	4/320	36/19520	0.002761	0.024284	0.020476
GO:000905MF	GO:000905electron	22/316	138/18337	2.28E-15	1.12E-12	9.85E-13
GO:000395MF	GO:000395NADH dehy	13/316	45/18337	4.11E-13	5.03E-11	4.44E-11
GO:000813MF	GO:000813NADH dehy	13/316	45/18337	4.11E-13	5.03E-11	4.44E-11
GO:005013MF	GO:005013NADH dehy	13/316	45/18337	4.11E-13	5.03E-11	4.44E-11
GO:000395MF	GO:000395NAD(P)H d	13/316	48/18337	1.04E-12	1.02E-10	8.96E-11
GO:000373MF	GO:000373structura	21/316	180/18337	5.34E-12	4.35E-10	3.84E-10
GO:001665MF	GO:001665oxidoredu	13/316	60/18337	2.31E-11	1.61E-09	1.42E-09
GO:001665MF	GO:001665oxidoredu	15/316	102/18337	2.28E-10	1.39E-08	1.23E-08
GO:005128MF	GO:005128NAD bindi	11/316	53/18337	1.34E-09	7.28E-08	6.42E-08
GO:00169CMF	GO:00169Coxidoredu	6/316	44/18337	0.000102	0.004967	0.004383
GO:001984MF	GO:001984rRNA bind	7/316	64/18337	0.000113	0.005027	0.004437
GO:001687MF	GO:001687ligase ac	11/316	163/18337	0.000125	0.005113	0.004513
GO:004803MF	GO:004803quinone b	4/316	18/18337	0.000219	0.008191	0.007229
GO:001661MF	GO:001661oxidoredu	9/316	120/18337	0.000235	0.008191	0.007229
GO:005066MF	GO:005066NADP bind	6/316	53/18337	0.00029	0.009457	0.008347
GO:001662MF	GO:001662oxidoredu	5/316	36/18337	0.000358	0.010943	0.009658
GO:001661MF	GO:001661oxidoredu	9/316	130/18337	0.000425	0.01223	0.010794
GO:00043CMF	GO:00043Cenoyl-CoA	3/316	10/18337	0.000556	0.015106	0.013332

GO:000813	MF	GO:000813	translati	7/316	84/18337	0.000615	0.015832	0.013973
GO:004518	MF	GO:004518	translati	9/316	138/18337	0.000657	0.016056	0.01417
GO:005153	MF	GO:005153	iron-sulf	6/316	67/18337	0.001031	0.022919	0.020228
GO:005154	MF	GO:005154	metal clu	6/316	67/18337	0.001031	0.022919	0.020228
GO:003503	MF	GO:003503	histone a	4/316	27/18337	0.00111	0.023608	0.020835
GO:003329	MF	GO:003329	monocarbo	6/316	71/18337	0.001397	0.02847	0.025127
GO:001683	MF	GO:001683	carbon-ca	5/316	51/18337	0.001808	0.035359	0.031207
GO:00704C	MF	GO:00704C	NADPH bin	3/316	15/18337	0.001978	0.037197	0.032829
GO:009007	MF	GO:009007	translati	7/316	108/18337	0.00267	0.04835	0.042673

geneID	Count
MRPS33/MR	31
LYRM7/NDU	35
MRPS33/MR	32
MRPS33/MR	24
LYRM7/NDU	25
MRPS33/MR	23
LYRM7/NDU	35
MRPS33/MR	26
MRPS33/MR	23
NDUFB5/ND	21
NDUFB5/ND	21
NDUFB5/TI	18
NDUFB5/TI	18
NDUFB5/ND	26
NDUFB5/ND	21
NDUFB5/ND	23
DLAT/PDHE	18
NDUFB5/ND	15
DLAT/PDHE	12
MRPS33/MR	24
NDUFB5/ND	25
TIMM21/DN	23
MRPS33/MR	25
DLAT/PDHE	9
DLAT/PDHE	13
DLAT/PDHE	13
TIMM21/TA	7
DLAT/PDHE	14
DLAT/PDHE	14
DLAT/PDHE	14
TIMM21/DN	14
LYRM7/TIM	8
TIMM21/DN	14
TIMM21/TA	7
TIMM21/SC	24
DLAT/PDHE	22
TIMM21/DN	11
APOOL/AGK	13
DLAT/PDHE	6
TIMM21/DN	11
TACO1/COA	6
DECR1/SCP	22
DECR1/SCP	16
TACO1/COA	6
DECR1/SCP	15
TIMM21/DN	5
TIMM21/DN	6
DLAT/PDHE	7
DLAT/PDHE	7
CUL4A/PAR	6
PHB/PMPCE	5
TIMM21/DN	7
DLAT/PDHE	4

DLAT/PDHE	20
DLAT/PDHE	19
DLAT/PDHE	7
DLAT/PDHE	7
DLAT/PDHE	7
DLAT/PDHE	19
DLAT/PDHE	18
TIMM21/DN	6
AIMP2/FAR	11
AGK/TOMM2	6
DECR1/SCP	7
AGK/TOMM2	7
MCCC1/BCK	4
APOOL/AGK	6
DECR1/SCP	8
DECR1/SCP	8
DLAT/PDHE	11
DLAT/PDHE	18
SCP2/HSD1	7
DECR1/SCP	8
PHB/CREB1	3
MCCC1/BCK	4
FASTKD1/M	17
AKR7A2/AD	6
CREB1/PSM	3
CLSTN3/DE	3
MLH1/PCID	11
AGK/TOMM2	15
SCP2/HSD1	6
SCP2/HSD1	6
SCP2/HSD1	6
FASTKD1/G	5
SMDT1/VDA	3
DLAT/PDHE	10
SCP2/HSD1	6
AIMP2/MCC	14
POLR2B/CU	6
CYC1/CYCS	3
CREB1/PSM	3
MTIF2/EIF	10
FARS2/GRS	8
DECR1/SCP	8
EIF3M/RPL	7
DLAT/PDHE	10
DECR1/SCP	11
PPID/TOP2	4
LYRM7/MRP	79
MRPS33/ND	60
MRPS33/ND	72
NDUFB5/PH	30
NDUFB5/ND	23
NDUFB5/DL	23
MRPS33/MR	20
MRPS33/MR	20

NDUFB5/ND	20
MRPS33/MR	26
MRPS33/MR	28
NDUFB5/ND	18
NDUFB5/ND	13
NDUFB5/ND	13
NDUFB5/ND	13
MRPL16/MR	16
APOOL/AGK	12
APOOL/AGK	12
APOOL/AGK	14
MRPL16/MR	12
MRPL16/MR	12
APOOL/AGK	14
MRPS33/MR	8
MRPS33/MR	8
MRPS33/MR	10
SUCLA2/FH	5
APOOL/AGK	19
PHB/APOOL	14
PHB/APOOL	14
PHB/APOOL	13
APOOL/TOM	5
APOOL/AGK	19
APOOL/SAM	4
APOOL/SAM	4
TIMM21/DN	4
GRSF1/TFA	6
GRSF1/TFA	6
ISOC1/PMV	9
ISOC1/PMV	9
NDUFA8/AG	7
SCP2/HSD1	5
SCP2/HSD1	5
NDUFA8/AG	6
CYC1/COX4	4
NDUFB5/ND	22
NDUFB5/ND	13
NDUFB5/ND	13
NDUFB5/ND	13
NDUFB5/ND	13
MRPS33/MR	21
NDUFB5/ND	13
NDUFB5/ND	15
IDH3B/NDU	11
DLAT/PDHE	6
MRPL16/IM	7
MCCC1/SUC	11
SDHD/NDUF	4
IDH3B/HSD	9
DECR1/GLY	6
DLAT/PDHE	5
IDH3B/HSD	9
HSD17B4/H	3

MTIF2/EIF	7
MTIF2/EIF	9
NDUFS2/FD	6
NDUFS2/FD	6
CREB1/PCN	4
NDUFAB1/S	6
ALDOC/MLY	5
DECR1/QDF	3
MTIF2/EIF	7

ID	Descripti	setSize	enrichmen	NES	pvalue	p.adjust	qvalues	rank
R-HSA-162	R-HSA-162	Signal Tr	66	0.328974	1.431172	0.064581	0.788133	0.788133
R-HSA-900	R-HSA-900	Signaling	13	0.432158	1.329429	0.156655	0.788133	0.788133
R-HSA-212	R-HSA-212	Generic T	16	0.399279	1.29945	0.16916	0.788133	0.788133
R-HSA-565	R-HSA-565	Vesicle-m	28	-0.18773	-1.21009	0.1875	0.788133	0.788133
R-HSA-199	R-HSA-199	Membrane	27	-0.18705	-1.17242	0.243243	0.788133	0.788133
R-HSA-125	R-HSA-125	PIP3 acti	10	0.427137	1.206476	0.248775	0.788133	0.788133
R-HSA-900	R-HSA-900	Intracell	10	0.427137	1.206476	0.248775	0.788133	0.788133
R-HSA-738	R-HSA-738	RNA Polym	17	0.363347	1.207176	0.259977	0.788133	0.788133
R-HSA-422	R-HSA-422	Axon guid	10	-0.29492	-1.10029	0.290323	0.788133	0.788133
R-HSA-967	R-HSA-967	Nervous s	10	-0.29492	-1.10029	0.290323	0.788133	0.788133
R-HSA-388	R-HSA-388	GPCR down	10	0.401213	1.133252	0.314951	0.788133	0.788133
R-HSA-741	R-HSA-741	Gene expr	18	0.330724	1.117489	0.357466	0.788133	0.788133
R-HSA-164	R-HSA-164	Disease	38	0.274619	1.091331	0.358193	0.788133	0.788133
R-HSA-128	R-HSA-128	Adaptive	10	0.362803	1.024761	0.422794	0.788133	0.788133
R-HSA-126	R-HSA-126	Developme	17	0.315542	1.04835	0.425314	0.788133	0.788133
R-HSA-760	R-HSA-760	Platelet	11	-0.2551	-1.00466	0.435294	0.788133	0.788133
R-HSA-147	R-HSA-147	Extracell	11	-0.2551	-1.00466	0.435294	0.788133	0.788133
R-HSA-372	R-HSA-372	Signaling	12	0.33711	1.009294	0.457988	0.788133	0.788133
R-HSA-566	R-HSA-566	Infectiou	20	0.282487	0.973439	0.50056	0.788133	0.788133
R-HSA-449	R-HSA-449	Signaling	22	0.269247	0.950952	0.529801	0.788133	0.788133
R-HSA-901	R-HSA-901	RHO GTPas	16	0.29131	0.948067	0.533947	0.788133	0.788133
R-HSA-713	R-HSA-713	Metabolis	10	-0.25085	-0.93588	0.543011	0.788133	0.788133
R-HSA-556	R-HSA-556	Metabolis	12	0.310345	0.929159	0.553846	0.788133	0.788133
R-HSA-446	R-HSA-446	Asparagin	12	-0.22152	-0.90141	0.573248	0.788133	0.788133
R-HSA-913	R-HSA-913	Interfero	11	0.30879	0.894872	0.59976	0.788133	0.788133
R-HSA-392	R-HSA-392	Metabolis	48	0.218571	0.909993	0.602683	0.788133	0.788133
R-HSA-109	R-HSA-109	Hemostasi	15	0.278801	0.8919	0.611819	0.788133	0.788133
R-HSA-128	R-HSA-128	Cytokine	31	0.231954	0.89621	0.613177	0.788133	0.788133
R-HSA-194	R-HSA-194	Signaling	22	0.247718	0.874915	0.618102	0.788133	0.788133
R-HSA-971	R-HSA-971	Signaling	22	0.247718	0.874915	0.618102	0.788133	0.788133
R-HSA-164	R-HSA-164	Cell Cycl	10	-0.2388	-0.89094	0.61828	0.788133	0.788133
R-HSA-168	R-HSA-168	Immune Sy	62	0.209045	0.894713	0.644831	0.788133	0.788133
R-HSA-597	R-HSA-597	Post-tran	41	0.21799	0.879245	0.65021	0.788133	0.788133
R-HSA-895	R-HSA-895	Cellular	13	-0.18836	-0.83299	0.732026	0.844018	0.844018
R-HSA-382	R-HSA-382	Transport	13	0.255529	0.786072	0.738516	0.844018	0.844018
R-HSA-679	R-HSA-679	Neutrophi	20	0.206945	0.713125	0.796193	0.873065	0.873065
R-HSA-168	R-HSA-168	Innate Im	29	0.186201	0.703997	0.817888	0.873065	0.873065
R-HSA-226	R-HSA-226	Cellular	11	-0.18707	-0.73675	0.829412	0.873065	0.873065
R-HSA-895	R-HSA-895	Metabolis	10	-0.17627	-0.65764	0.930108	0.93808	0.93808
R-HSA-143	R-HSA-143	Metabolis	45	0.140045	0.578859	0.93808	0.93808	0.93808

leading_ϵ	core_enrichment
154 tags=65%,	7433/1124/80820/56998/11221/5729/9938/151306/3556/5899/9021/102
153 tags=85%,	5899/10252/55824/3726/7057/9261/6455/3643/5595/6777/6774
143 tags=75%,	4094/1436/9021/6881/3726/1912/7057/3280/27161/7533/5595/5054
254 tags=96%,	51542/2590/11021/11031/1176/6455/7533/5872/1604/6845/10802/6811
254 tags=96%,	51542/2590/11021/11031/1176/6455/7533/5872/1604/6845/10802/6811
149 tags=80%,	3556/1912/1822/391/3643/27161/5595/9173
149 tags=80%,	3556/1912/1822/391/3643/27161/5595/9173
143 tags=71%,	4094/1436/9021/6881/3726/1912/7057/3280/27161/7533/5595/5054
219 tags=100%	200916/5595/1942/6710/6324/1212/3690/28964/10094
219 tags=100%	200916/5595/1942/6710/6324/1212/3690/28964/10094
134 tags=70%,	7433/5729/151306/3274/2783/2769/5595
143 tags=67%,	4094/1436/9021/6881/3726/1912/7057/3280/27161/7533/5595/5054
124 tags=50%,	7433/11221/5729/686/151306/1052/6881/1545/7057/57085/3274/3280/
89 tags=50%,	10219/9021/55824/11275/8519
153 tags=65%,	1879/386678/1052/3985/3280/91/200916/5595/1942/5316/6774
231 tags=100%	2783/391/2769/5595/5054/2782/7076/54676/2773/3690
231 tags=100%	8728/1462/5054/3693/5352/960/7076/4323/79709/3690
146 tags=67%,	7433/5729/151306/3274/2783/2769/5595/1604
134 tags=55%,	7433/5729/151306/1052/6881/3274/2783/6455/3554/200916/5595
153 tags=64%,	1436/3556/9021/3566/1052/79155/3726/7132/9261/3554/5595/6777/91
172 tags=75%,	1124/9938/9732/2040/2319/391/10211/23221/79180/5756/6811/23074
232 tags=100%	1462/5836/960/4928/3098/2023/22856/7086/5236
166 tags=75%,	1545/10558/7357/142891/9261/9489/114880/7376/10802
161 tags=83%,	1604/10802/6811/81876/6710/256435/11316/85365/378/9695
2 tags=9%,	3434
186 tags=73%,	63917/9021/1453/6881/79155/249/1912/9334/7057/4924/2590/11275/1
200 tags=87%,	8140/9732/7057/2783/391/2769/5595/5054/140885/960/2782/7076/546
97 tags=35%,	3434/1436/3556/9021/3566/1052/79155/3726/8519/7132/9261
172 tags=68%,	1124/9938/9732/2040/3985/2319/391/10211/7533/5595/23221/79180/5
172 tags=68%,	1124/9938/9732/2040/3985/2319/391/10211/7533/5595/23221/79180/5
180 tags=90%,	7533/5595/51720/4928/26277/81876/902/22919/57804
169 tags=61%,	3434/10219/1436/3556/9961/9021/3566/837/55824/1052/79155/3726/1
170 tags=66%,	63917/9021/1453/6881/79155/249/1912/9334/7057/4924/2590/11275/1
251 tags=100%	1912/9261/200916/5595/6774/4928/26277/91582/4520/3091/4495/6782
43 tags=23%,	94015/3077/8140
152 tags=60%,	9961/127829/2040/11031/5806/391/6282/5836/4860/1604/140885/1992
152 tags=55%,	9961/837/79155/127829/2040/9261/11031/5806/391/6282/5836/5595/4
251 tags=100%	1912/9261/200916/5595/6774/4928/26277/91582/3091/6782
254 tags=30%,	10644/8780/8896
219 tags=78%,	686/285440/8140/1545/7378/10558/9334/5447/7357/142891/9261/1462

52/55824/6788/3726/9732/1912/7057/2040/3274/3985/7132/3280/9261/2783/91/1822/6455/

/3949/64786/57186/3267/81876/6710/11316/2664/2495/378/1212/10094/8685/1362/51762
/3949/64786/57186/3267/81876/6710/11316/2664/2495/378/1212/10094/1362/51762

2120/1462/2783/6455/3554/391/200916

73/6774

1021/1462/11031/2783/2769/200916/5872/8450/55647/1604/51720/6774/79091/10318/9960/
76

756/6811/23074
756/6811/23074

27829/2040/11275/8519/7132/9261/11031/5806/3554/391/6282/5836/84231/5595/4860/677'
1021/1462/11031/5872/8450/55647/1604/51720/79091/10318/9960/7376/10802/6672/6811

860/1604/140885/1992

/2783/9489/2769/200916/114880/5836/4860/7376/10802/960/2782/81894/3949/79602/4928/

/2319/391/3643/2769/10211/27161/7533/5595/23221/5054/6777/1604/9173/6774/79180

/7376/10802/6672/6811/2782/3068/7076/4928

7/1604/140885/9173/1992/6774/3693/6845/10802/6672/960

/2745/3098/2023/2730/166929/6303/4520/26020

ID	Descriptiset	Size	enrichmen	NES	pvalue	p.adjust	qvalues	rank
R-HSA-142	R-HSA-142	The citri	39	0.201412	1.485306	0.02	0.378917	0.367371
R-HSA-168	R-HSA-168	Immune Sy	14	-0.46608	-1.59705	0.035152	0.378917	0.367371
R-HSA-536	R-HSA-536	Mitochond	22	0.280396	1.61399	0.049505	0.378917	0.367371
R-HSA-422	R-HSA-422	Axon guid	12	-0.46996	-1.52973	0.057285	0.378917	0.367371
R-HSA-967	R-HSA-967	Nervous s	12	-0.46996	-1.52973	0.057285	0.378917	0.367371
R-HSA-895	R-HSA-895	Metabolis	22	-0.37372	-1.4666	0.065483	0.378917	0.367371
R-HSA-541	R-HSA-541	Mitochond	21	0.258755	1.461685	0.080808	0.378917	0.367371
R-HSA-597	R-HSA-597	Post-tran	25	-0.35587	-1.44877	0.081967	0.378917	0.367371
R-HSA-126	R-HSA-126	Developme	17	-0.39625	-1.43837	0.090909	0.378917	0.367371
R-HSA-536	R-HSA-536	Mitochond	24	0.233331	1.375976	0.113636	0.378917	0.367371
R-HSA-714	R-HSA-714	Pyruvate	15	0.305195	1.417002	0.118421	0.378917	0.367371
R-HSA-556	R-HSA-556	Metabolis	25	-0.33504	-1.36395	0.12459	0.378917	0.367371
R-HSA-164	R-HSA-164	Cell Cycl	13	-0.40715	-1.35363	0.12963	0.378917	0.367371
R-HSA-226	R-HSA-226	Cellular	20	-0.34444	-1.31146	0.159683	0.40453	0.392204
R-HSA-895	R-HSA-895	Cellular	20	-0.34444	-1.31146	0.159683	0.40453	0.392204
R-HSA-566	R-HSA-566	Infectiou	19	-0.34159	-1.28159	0.180682	0.425532	0.412566
R-HSA-212	R-HSA-212	Generic T	26	-0.30365	-1.2523	0.198697	0.425532	0.412566
R-HSA-738	R-HSA-738	RNA Polym	29	-0.29429	-1.23947	0.208378	0.425532	0.412566
R-HSA-538	R-HSA-538	Mitochond	23	0.20479	1.19682	0.212766	0.425532	0.412566
R-HSA-712	R-HSA-712	Metabolis	24	-0.30112	-1.21554	0.237418	0.451094	0.437349
R-HSA-741	R-HSA-741	Gene expr	33	-0.26882	-1.16905	0.278252	0.503503	0.488161
R-HSA-392	R-HSA-392	Metabolis	64	-0.23042	-1.13065	0.32622	0.516421	0.500685
R-HSA-679	R-HSA-679	Complex I	16	0.219388	1.05142	0.351351	0.516421	0.500685
R-HSA-162	R-HSA-162	Signal Tr	30	-0.25471	-1.08122	0.370331	0.516421	0.500685
R-HSA-163	R-HSA-163	Respirato	25	0.178116	1.063023	0.37931	0.516421	0.500685
R-HSA-611	R-HSA-611	Respirato	25	0.178116	1.063023	0.37931	0.516421	0.500685
R-HSA-168	R-HSA-168	Influenza	10	-0.34038	-1.05088	0.389313	0.516421	0.500685
R-HSA-168	R-HSA-168	Influenza	10	-0.34038	-1.05088	0.389313	0.516421	0.500685
R-HSA-382	R-HSA-382	Transport	20	-0.28085	-1.06935	0.394111	0.516421	0.500685
R-HSA-126	R-HSA-126	Mitochond	14	0.203883	0.936015	0.508475	0.644068	0.624442
R-HSA-370	R-HSA-370	Transcrip	14	-0.27578	-0.94497	0.530909	0.650792	0.630962
R-HSA-738	R-HSA-738	DNA Repai	11	-0.28383	-0.90149	0.564617	0.670483	0.650053
R-HSA-960	R-HSA-960	Protein l	20	0.165017	0.911404	0.605042	0.686675	0.665751
R-HSA-164	R-HSA-164	Disease	28	-0.21279	-0.89357	0.614393	0.686675	0.665751
R-HSA-897	R-HSA-897	Fatty aci	11	-0.26489	-0.84132	0.6399	0.694748	0.673579
R-HSA-143	R-HSA-143	Metabolis	95	-0.17157	-0.88294	0.681087	0.718925	0.697018
R-HSA-727	R-HSA-727	Translati	37	-0.1634	-0.73095	0.790747	0.812118	0.787372
R-HSA-900	R-HSA-900	Signaling	10	-0.22502	-0.69471	0.812977	0.812977	0.788205

leading_ecore_enrichment

240 tags=95%,4711/1737/5162/4706/4704/51204/1537/29078/4722/6392/4717/4702/5
104 tags=64%,8453/79876/5705/11059/3313/5702/201626/1378/79792
88 tags=64%,51650/54948/64432/51642/4528/54148/64981/10884/55173/51373/5108
159 tags=92%,6156/6233/9045/8453/5705/6146/6138/6228/5702/84448/782
159 tags=92%,6156/6233/9045/8453/5705/6146/6138/6228/5702/84448/782
159 tags=77%,6156/6233/51808/29883/9045/23658/51493/6636/5705/84991/6146/233
88 tags=62%,51650/54948/64432/51642/54148/64981/10884/55173/51373/51081/650
187 tags=84%,51227/6596/8451/142/6233/7155/5111/25839/7416/1603/10600/9382/9
165 tags=82%,10664/6156/6233/9045/8453/5705/6146/6138/6228/5438/5702/84448/7
26 tags=33%,51650/54948/64432/51642/4528/54148/64981/10884
229 tags=100%1737/5162/6392/3420/8803/1431/2271/8801/4191/8050/5160/23530/74
62 tags=32%,3030/1632/5019/6609/4597/660/388121/5742
168 tags=85%,4660/5048/6233/5111/5705/5885/5438/5884/5702/7532/81620
192 tags=85%,1385/9575/1327/10128/6156/6233/9045/8453/5705/116228/6146/6138/
192 tags=85%,1385/9575/1327/10128/6156/6233/9045/8453/5705/116228/6146/6138/
122 tags=58%,9045/1603/5705/6146/10284/6138/6228/5438/5702/6483/79792
94 tags=42%,5705/116228/11059/9377/168374/5438/5884/5702/7532/1051/1378
102 tags=45%,6636/5705/116228/11059/26097/9377/168374/5438/5884/5702/7532/10
26 tags=30%,51650/54948/64432/51642/54148/64981/10884
164 tags=71%,2806/6156/6233/64087/8050/5160/9045/2805/1743/5705/6146/9380/61
102 tags=42%,6636/5705/116228/10284/11059/26097/9377/168374/5438/5884/5702/7
130 tags=48%,5111/25839/7416/9045/23107/1603/10600/9382/93973/8453/5705/8547
93 tags=62%,4711/4706/4704/29078/4722/4717/4702/4726/4720/55744
172 tags=67%,142/4660/5481/5048/6233/8050/5160/51566/79971/5705/25813/5037/1
63 tags=48%,4711/4706/4704/51204/1537/29078/4722/6392/4717/4702/54205/4726
63 tags=48%,4711/4706/4704/51204/1537/29078/4722/6392/4717/4702/54205/4726
172 tags=80%,142/6156/6233/9045/6146/6138/6228/5438
172 tags=80%,142/6156/6233/9045/6146/6138/6228/5438
106 tags=45%,10939/5705/11059/9194/5824/5702/488/341/6582
260 tags=100%29090/1537/131118/56993/1431/9512/10469/617/7416/92609/25813/47
180 tags=71%,1327/10128/6233/29883/5111/116228/9377/5438/5884/7532
175 tags=73%,8451/142/6233/5111/93973/91603/5438/5884
273 tags=100%29090/1537/6342/131118/56993/1431/3295/9512/373156/10469/617/62
94 tags=36%,5705/5037/6146/10284/6138/6228/5438/5702/6483/79792
62 tags=27%,3030/1632/5742
167 tags=54%,7923/10128/2806/4719/84532/6156/4191/6233/374291/64087/23417/80
92 tags=32%,85476/5018/27335/6146/10102/6138/64965/60488/6228/219927/23438/
67 tags=60%,5481/8050/5160/5885/5438/341

4205/4726/3420/8803/1431/4720/55744/2271/1327/8801/10128/4719/4191/374291/8050/51[illegible]

1/65080/65003/28973

98/6138/26097/6228/5438/5702
80/65003/28973
3973/8453/5705/5885/1725/10238/5702/4597/6483
82/1051

16/51660/1743

3313/9377/6228/5702/1051
3313/9377/6228/5702/1051

51/1378

38/6228/11112/262/5702
532/1051/1378
6/5018/27335/6146/10102/6138/5885/64965/60488/1725/6228/10238/219927/23438/5702/1[illegible]

1059/5885/5438/5702/7532/488/1627/341

14/2395/3313

33/23417/7416/92609/25813/4714/2395/3313/5824

50/5160/23171/79077/23530/57017/7416/9045/10975/25880/2805/55967/51660/1743/5705/[illegible]
116541

60/23530/7416/10975/25880/55967/51660/1743/116228/4714

0564/116541/4597/1051/6483

253558/116228/4714/53343/6146/9380/2395/6138/55163/3030/1632/9377/6228/4723/11112/

/5019/262/6609/5702/4597/660/388121/6483/6582/5742
