# Supplementary material for: Exploration of the Shared Gene Signatures between Myocardium and Blood in Sepsis: Evidence from Bioinformatics Analysis
Source: Biomed Res Int. 2022 Aug 6;2022:3690893. doi: 10.1155/2022/3690893 (PMC9375705; doi:10.1155/2022/3690893)
Supplement: Supplementary Materials — Supplement Figure S1. Correlations between different module memberships generated by weighted correlation network analysis (WGCNA). The gene significances were calculated and are presented in a scatter plot. Correlation coefficients and p-values are shown on the top of the figure. The blue, brown, grey, and turquoise modules were found to be linearly correlated with gene significance. Figure S2. The x-axis of the bar plot indicates the possible clustering method suggested by k-means clustering analysis, and the y-axis presents the evaluation scores of the different clustering methods. The k-means clustering analysis suggests that the best classification is dichotomous. B. The eigenvalue decreases rapidly as the component number increases at the beginning, especially when the first two components are added. The decrease in the eigenvalue was less obvious when more than three components were added. A scree plot shows that two components can well describe the characteristics of the groups clustered by the clustering analysis. Figure S3. Nomograph displaying the risk score of each risk factor when six genes were integrated with the age and appach II score, including SMU1, SP100, and ARHGAP25, which contribute great weight to mortality. Calibration curve in GSE54514, which shows the good fit of our model. The mean absolute error is acceptable (mean absolute error =0.033). Table S1. A total of 1,049 DEGs changed in the same way in both the myocardium and blood datasets, including 549 genes upregulated and 500 genes downregulated. There were 325 genes in the blue module, 116 genes in the brown module, 305 genes in the turquoise module, and 261 genes in the gray module. Table S2. Detailed results of Gene Ontology (GO) analysis and GeneSet Enrichment Analysis (GSEA) of the turquoise and blue modules, as supplementary material for Figure 2. This table displays all the GO analysis pathways, including biological process (BP), cell component (CC), and molecular function (MF), as [file 3690893.f1.zip › Table-S3.pdf]

	logFC	AveExpr	t	P.Value	adj.P.Val	B
NNMT	0.342916	2.502786	5.292224	1.86E-07	0.000195	6.797077
TNFRSF8	-0.23493	3.292691	-4.16932	3.64E-05	0.013354	2.029368
TBC1D22B	0.296145	4.190341	4.157861	3.82E-05	0.013354	1.986039
DEFA4	1.013594	7.411641	3.959555	8.67E-05	0.014683	1.253904
SELL	-0.15535	11.35196	-3.93622	9.53E-05	0.014683	1.169957
TMEM184C	-0.21338	4.327546	-3.89399	0.000113	0.014683	1.019153
CDT1	0.166466	2.542951	3.888952	0.000115	0.014683	1.001281
DYRK3	0.330194	3.149199	3.871441	0.000124	0.014683	0.93928
DECR1	-0.15811	8.480347	-3.86648	0.000126	0.014683	0.921772
MS4A3	0.921698	5.530657	3.767646	0.000186	0.017049	0.577165
ELANE	0.790014	5.092346	3.75942	0.000192	0.017049	0.54886
BEX1	0.508149	3.100919	3.747849	0.000201	0.017049	0.509142
VPS45	-0.22915	3.810502	-3.71752	0.000225	0.017049	0.405581
RPL22L1	0.333736	3.832398	3.715117	0.000228	0.017049	0.39742
RASSF4	-0.35489	5.0835	-3.65902	0.000282	0.019712	0.208085
NAGK	-0.27293	8.161813	-3.63516	0.000308	0.020225	0.128379
ADCY9	-0.10708	2.341201	-3.59571	0.000358	0.022075	-0.00234
TMBIM6	-0.14896	9.929749	-3.56746	0.000397	0.023163	-0.0951
TTYH2	-0.19885	3.468317	-3.52564	0.000464	0.024159	-0.2312
HSPA13	0.194693	2.777026	3.520448	0.000473	0.024159	-0.24797
ING3	-0.18855	4.758041	-3.50608	0.000498	0.024159	-0.29431
CA1	0.733339	8.019827	3.501583	0.000507	0.024159	-0.30877
TGM2	0.522611	4.619417	3.469241	0.00057	0.024577	-0.41229
NR1H2	-0.17011	4.399916	-3.46365	0.000582	0.024577	-0.43011
PEPD	-0.31578	4.434932	-3.46174	0.000586	0.024577	-0.43616
BLCAP	-0.12842	6.752393	-3.43363	0.000648	0.026156	-0.52523
SMU1	-0.13529	2.993983	-3.39519	0.000744	0.028904	-0.64587
ZYG11B	-0.21961	6.309437	-3.34617	0.000885	0.033159	-0.79787
LINC0026C	-0.25098	4.257325	-3.29412	0.001062	0.037887	-0.95696
RCBTB2	-0.36768	4.826725	-3.28633	0.001091	0.037887	-0.98056
NDRG3	-0.13047	2.771109	-3.27341	0.001141	0.037887	-1.01961
PPP1R15A	0.161099	4.274603	3.269681	0.001156	0.037887	-1.03084
CTSS	-0.19837	10.14557	-3.25028	0.001236	0.03928	-1.08912
MAP4K4	-0.22445	5.028615	-3.21189	0.001409	0.043477	-1.20346
MVP	-0.19094	5.964214	-3.18596	0.001539	0.045913	-1.27994
CEACAM6	0.285206	3.214222	3.169408	0.001627	0.045913	-1.32847
ARL4A	0.324161	3.360141	3.164722	0.001653	0.045913	-1.34216
TNFRSF1A	-0.22851	5.917219	-3.16291	0.001663	0.045913	-1.34745
IL1RL1	0.121374	2.430116	3.132625	0.001841	0.049511	-1.43543
NF2	-0.16332	3.636732	-3.11938	0.001924	0.05045	-1.47364
CLIC3	-0.28351	2.927964	-3.08841	0.002131	0.05453	-1.56241
TPGS2	0.437941	6.62749	3.075436	0.002224	0.05453	-1.59934
CD24	0.617399	7.997658	3.070073	0.002264	0.05453	-1.61457
RNF10	0.239141	9.083292	3.066637	0.00229	0.05453	-1.62431
PTER	-0.13826	2.680875	-3.05549	0.002375	0.05453	-1.65583
USP12	0.197192	2.81986	3.053383	0.002391	0.05453	-1.66178
FGL2	-0.40628	6.86245	-3.01407	0.002717	0.060642	-1.77201
ABLIM1	-0.31695	5.367436	-2.99861	0.002856	0.062417	-1.815
CYP4V2	-0.14115	2.584014	-2.96568	0.003174	0.066717	-1.90584
PAFAH1B1	-0.15795	7.149204	-2.96508	0.00318	0.066717	-1.90748

SLC7A5	0.275461	3.77339	2.932194	0.00353	0.069947	-1.9972
PPTC7	-0.23531	6.622161	-2.92266	0.003638	0.069947	-2.02302
SPOP	-0.12416	7.221904	-2.90246	0.003877	0.069947	-2.07749
COQ9	-0.12972	4.389259	-2.90007	0.003906	0.069947	-2.08391
RNF125	-0.10428	2.620371	-2.89954	0.003912	0.069947	-2.08534
PCYOX1L	-0.21035	4.246913	-2.89945	0.003913	0.069947	-2.08557
CYTH4	-0.25413	7.340458	-2.89886	0.003921	0.069947	-2.08717
UBALD2	0.145102	6.038382	2.894659	0.003972	0.069947	-2.09844
TMEM203	-0.16095	4.580903	-2.89209	0.004005	0.069947	-2.10533
GALNT11	-0.1621	4.440255	-2.88809	0.004055	0.069947	-2.11601
CENPV	0.102149	2.50235	2.884579	0.0041	0.069947	-2.12541
NME4	0.272613	4.135872	2.881884	0.004134	0.069947	-2.1326
DCAF7	-0.18752	3.749905	-2.87	0.00429	0.070003	-2.16425
CYTH3	-0.10791	2.499131	-2.86665	0.004335	0.070003	-2.17315
CSF1R	-0.21766	3.701546	-2.86149	0.004405	0.070003	-2.18684
SLC7A6OS	-0.11814	4.218848	-2.85512	0.004492	0.070003	-2.20371
YWHAH	0.203382	8.344383	2.853319	0.004518	0.070003	-2.20848
ARF4	0.140464	7.441546	2.850654	0.004555	0.070003	-2.21552
KLRG1	-0.24029	3.049982	-2.84176	0.004682	0.070003	-2.23898
ITGAX	-0.22098	8.382889	-2.84007	0.004706	0.070003	-2.24344
HBD	0.568749	8.544118	2.832237	0.004821	0.070003	-2.26401
SCAP	-0.14637	4.513184	-2.82951	0.004862	0.070003	-2.27116
DDHD2	0.15185	3.246071	2.828843	0.004872	0.070003	-2.27292
ZDHHC3	0.154995	5.326593	2.822581	0.004966	0.070397	-2.28932
MLYCD	-0.09424	4.674888	-2.80816	0.00519	0.072592	-2.32695
TPCN1	-0.16815	4.263789	-2.79956	0.005328	0.073108	-2.34929
C1RL	-0.22309	7.539733	-2.7972	0.005366	0.073108	-2.35543
TMEM187	-0.14611	3.291677	-2.79174	0.005456	0.07338	-2.36958
TSC1	-0.15302	5.447473	-2.78284	0.005606	0.073699	-2.39257
RPP25L	-0.18647	5.578126	-2.78197	0.00562	0.073699	-2.39483
FCGRT	-0.17934	9.295123	-2.77136	0.005804	0.074788	-2.42213
ICA1	0.175825	4.15506	2.768951	0.005846	0.074788	-2.42833
TNS3	-0.23748	4.424662	-2.75907	0.006023	0.075381	-2.45365
NIPAL3	-0.2005	3.929117	-2.75834	0.006036	0.075381	-2.45554
PNP	0.30464	7.423215	2.736383	0.006447	0.078944	-2.51149
THBS1	0.498414	5.628884	2.7351	0.006472	0.078944	-2.51475
ARHGAP25	-0.1936	8.030062	-2.71366	0.006899	0.083095	-2.56894
IFITM2	-0.11163	11.29157	-2.71017	0.006971	0.083095	-2.57772
SVIP	0.295224	5.460012	2.693822	0.007317	0.086237	-2.61873
LDLR	0.349445	4.586466	2.681807	0.00758	0.087576	-2.64871
IP6K2	-0.10008	5.215454	-2.68004	0.00762	0.087576	-2.65311
ABR	-0.13224	6.000599	-2.67734	0.007681	0.087576	-2.65982
THRA	0.196795	3.957019	2.667761	0.0079	0.087987	-2.6836
SLC7A7	-0.30401	6.560554	-2.6671	0.007915	0.087987	-2.68523
SP100	-0.1832	2.997817	-2.66481	0.007968	0.087987	-2.6909
IL1R2	0.369333	11.07113	2.658801	0.00811	0.088151	-2.70576
COL18A1	-0.14535	3.43806	-2.65705	0.008151	0.088151	-2.71008
ST3GAL5	-0.16507	4.724354	-2.63053	0.008805	0.094251	-2.7752
LRRN3	-0.19212	2.661019	-2.61868	0.009112	0.096552	-2.80409
GSTK1	-0.15219	8.502672	-2.61387	0.00924	0.096748	-2.81579
HDGF	0.216642	9.004856	2.61105	0.009315	0.096748	-2.82264

FAM78A	-0.15282	6.111117	-2.60358	0.009518	0.097881	-2.84073
CDK19	-0.16026	6.54769	-2.5876	0.009964	0.09946	-2.87928
HARS2	-0.10557	3.048912	-2.58683	0.009986	0.09946	-2.88112
PDHB	-0.14662	6.226812	-2.58644	0.009997	0.09946	-2.88208
