# Supplementary material for: Exploration of the Shared Gene Signatures between Myocardium and Blood in Sepsis: Evidence from Bioinformatics Analysis
Source: Biomed Res Int. 2022 Aug 6;2022:3690893. doi: 10.1155/2022/3690893 (PMC9375705; doi:10.1155/2022/3690893)
Supplement: Supplementary Materials — Supplement Figure S1. Correlations between different module memberships generated by weighted correlation network analysis (WGCNA). The gene significances were calculated and are presented in a scatter plot. Correlation coefficients and p-values are shown on the top of the figure. The blue, brown, grey, and turquoise modules were found to be linearly correlated with gene significance. Figure S2. The x-axis of the bar plot indicates the possible clustering method suggested by k-means clustering analysis, and the y-axis presents the evaluation scores of the different clustering methods. The k-means clustering analysis suggests that the best classification is dichotomous. B. The eigenvalue decreases rapidly as the component number increases at the beginning, especially when the first two components are added. The decrease in the eigenvalue was less obvious when more than three components were added. A scree plot shows that two components can well describe the characteristics of the groups clustered by the clustering analysis. Figure S3. Nomograph displaying the risk score of each risk factor when six genes were integrated with the age and appach II score, including SMU1, SP100, and ARHGAP25, which contribute great weight to mortality. Calibration curve in GSE54514, which shows the good fit of our model. The mean absolute error is acceptable (mean absolute error =0.033). Table S1. A total of 1,049 DEGs changed in the same way in both the myocardium and blood datasets, including 549 genes upregulated and 500 genes downregulated. There were 325 genes in the blue module, 116 genes in the brown module, 305 genes in the turquoise module, and 261 genes in the gray module. Table S2. Detailed results of Gene Ontology (GO) analysis and GeneSet Enrichment Analysis (GSEA) of the turquoise and blue modules, as supplementary material for Figure 2. This table displays all the GO analysis pathways, including biological process (BP), cell component (CC), and molecular function (MF), as [file 3690893.f1.zip › Table-S4.pdf]

	MeanDecreaseAccuracy	gene
SMU1	5.304062821	SMU1
NAGK	5.218135635	NAGK
COQ9	5.169187401	COQ9
TMEM184C	4.947692276	TMEM184C
PDHB	4.927717371	PDHB
FAM78A	4.915031032	FAM78A
GSTK1	4.684605352	GSTK1
NDRG3	4.634844145	NDRG3
TMBIM6	4.609383615	TMBIM6
CLIC3	4.422406173	CLIC3
THRA	4.371281054	THRA
DECR1	4.244727676	DECR1
LDLR	4.240282389	LDLR
KLRG1	4.155797321	KLRG1
PPP1R15A	4.002550381	PPP1R15A
RNF10	3.836212063	RNF10
BLCAP	3.785009095	BLCAP
DEFA4	3.726951485	DEFA4
ZYG11B	3.6837977	ZYG11B
RPL22L1	3.67751341	RPL22L1
TPGS2	3.563918731	TPGS2
ELANE	3.524768993	ELANE
DYRK3	3.500402446	DYRK3
VPS45	3.465436697	VPS45
TMEM187	3.271519554	TMEM187
SP100	3.167086139	SP100
PAFAH1B1	3.164391985	PAFAH1B1
CDT1	3.123601363	CDT1
ING3	3.116150921	ING3
ARHGAP25	3.110078732	ARHGAP25
RCBTB2	3.085957247	RCBTB2
CSF1R	3.047830287	CSF1R
NNMT	3.007637665	NNMT
MS4A3	3.005286974	MS4A3
TBC1D22B	2.989474509	TBC1D22B
FCGRT	2.951788273	FCGRT
TTYH2	2.945643783	TTYH2
SPOP	2.936579209	SPOP
USP12	2.936383822	USP12
CYTH4	2.929770357	CYTH4
HDGF	2.904844305	HDGF
CA1	2.857001445	CA1
NR1H2	2.853248366	NR1H2
NIPAL3	2.839136636	NIPAL3
TSC1	2.812716117	TSC1
COL18A1	2.802657221	COL18A1
HSPA13	2.778896891	HSPA13
CD24	2.750336769	CD24
DDHD2	2.749496193	DDHD2
MLYCD	2.734248972	MLYCD

IL1RL1	2.675181539	IL1RL1
PNP	2.637851755	PNP
TNFRSF1A	2.570949418	TNFRSF1A
RPP25L	2.519769783	RPP25L
TNFRSF8	2.509519015	TNFRSF8
TGM2	2.497822148	TGM2
TMEM203	2.436614375	TMEM203
BEX1	2.33506544	BEX1
SVIP	2.179335751	SVIP
HARS2	2.155669542	HARS2
ADCY9	2.143029511	ADCY9
ZDHHC3	2.133841303	ZDHHC3
MAP4K4	2.124031128	MAP4K4
ARF4	2.111906098	ARF4
RASSF4	2.066751862	RASSF4
SLC7A6OS	2.038829753	SLC7A6OS
NF2	1.984338313	NF2
HBD	1.842769396	HBD
CEACAM6	1.816608678	CEACAM6
CTSS	1.805858918	CTSS
DCAF7	1.801275778	DCAF7
UBALD2	1.783542182	UBALD2
SCAP	1.778351932	SCAP
NME4	1.771993379	NME4
SELL	1.735643638	SELL
ICA1	1.638606206	ICA1
ABR	1.598586778	ABR
CDK19	1.561439907	CDK19
PPTC7	1.536583599	PPTC7
MVP	1.468429203	MVP
THBS1	1.403372187	THBS1
C1RL	1.359469405	C1RL
ST3GAL5	1.325443808	ST3GAL5
CYTH3	1.2842556	CYTH3
PTER	1.274151812	PTER
YWHAH	1.250197837	YWHAH
ABLIM1	1.197971369	ABLIM1
SLC7A7	1.071828077	SLC7A7
ARL4A	1.023232041	ARL4A
FGL2	1.008872124	FGL2
TPCN1	0.936967565	TPCN1
ITGAX	0.835697468	ITGAX
CYP4V2	0.785945942	CYP4V2
PEPD	0.746206735	PEPD
SLC7A5	0.741813131	SLC7A5
IP6K2	0.397834627	IP6K2
CENPV	0.380547627	CENPV
RNF125	0.364028056	RNF125
PCYOX1L	0.329613768	PCYOX1L
LRRN3	0.158078906	LRRN3
GALNT11	0.154095602	GALNT11

IL1R2	-0.004911628	IL1R2
LINC0026C	-0.547302128	LINC00260
IFITM2	-0.647070331	IFITM2
TNS3	-0.755325443	TNS3

	MeanDecreaseGir	gene
NNMT	3.095474357	NNMT
ADCY9	2.534735672	ADCY9
CLIC3	2.493865539	CLIC3
HSPA13	2.457897228	HSPA13
THRA	2.353394487	THRA
NDRG3	2.33023231	NDRG3
RPL22L1	2.304242235	RPL22L1
SP100	2.281420327	SP100
PPP1R15A	2.268718111	PPP1R15A
IL1RL1	2.263559729	IL1RL1
NR1H2	2.217662115	NR1H2
COQ9	2.153887869	COQ9
TPCN1	2.080577492	TPCN1
KLRG1	2.07288653	KLRG1
RCBTB2	2.066987885	RCBTB2
ELANE	2.039952744	ELANE
SMU1	1.978466295	SMU1
CYP4V2	1.973182381	CYP4V2
SVIP	1.970860306	SVIP
TTYH2	1.870575137	TTYH2
ARL4A	1.842743866	ARL4A
CDT1	1.838803116	CDT1
BEX1	1.816495888	BEX1
TNFRSF8	1.812133255	TNFRSF8
TMEM184C	1.803817049	TMEM184C
TNS3	1.773965863	TNS3
DEFA4	1.765558874	DEFA4
ABLIM1	1.764222245	ABLIM1
YWHAH	1.763901231	YWHAH
PTER	1.752806231	PTER
LDLR	1.743148265	LDLR
TBC1D22B	1.709182304	TBC1D22B
ABR	1.693732573	ABR
MLYCD	1.683095101	MLYCD
LRRN3	1.655130531	LRRN3
BLCAP	1.651933079	BLCAP
PEPD	1.648108349	PEPD
ZDHHC3	1.636574813	ZDHHC3
ITGAX	1.631152843	ITGAX
DCAF7	1.630412446	DCAF7
NME4	1.624366669	NME4
TMEM187	1.622914454	TMEM187
PDHB	1.61000936	PDHB
MS4A3	1.589661799	MS4A3
CD24	1.585356565	CD24
IL1R2	1.532199163	IL1R2
THBS1	1.490155733	THBS1
SLC7A5	1.488957948	SLC7A5
CENPV	1.462638164	CENPV
SPOP	1.461487987	SPOP

PAFAH1B1	1.436314493	PAFAH1B1
ARF4	1.43508261	ARF4
FAM78A	1.432298276	FAM78A
CYTH4	1.420759927	CYTH4
MVP	1.410096925	MVP
SLC7A6OS	1.405999787	SLC7A6OS
DECR1	1.403225066	DECR1
FGL2	1.391979661	FGL2
CYTH3	1.381821259	CYTH3
TGM2	1.374630787	TGM2
GSTK1	1.372639958	GSTK1
ING3	1.371442071	ING3
UBALD2	1.368664591	UBALD2
PCYOX1L	1.338867358	PCYOX1L
VPS45	1.314909804	VPS45
ARHGAP25	1.272044296	ARHGAP25
DDHD2	1.262485456	DDHD2
SELL	1.261472179	SELL
NF2	1.248733509	NF2
RASSF4	1.226991339	RASSF4
NIPAL3	1.221301245	NIPAL3
TMBIM6	1.215259321	TMBIM6
LINC00260	1.200134377	LINC00260
IP6K2	1.198340326	IP6K2
GALNT11	1.197656314	GALNT11
CEACAM6	1.182514514	CEACAM6
NAGK	1.178890305	NAGK
USP12	1.178025513	USP12
ST3GAL5	1.166311471	ST3GAL5
RNF125	1.162929492	RNF125
ZYG11B	1.157989087	ZYG11B
C1RL	1.154899178	C1RL
FCGRT	1.152416434	FCGRT
TNFRSF1A	1.146857872	TNFRSF1A
TPGS2	1.12598203	TPGS2
CA1	1.11904931	CA1
SCAP	1.118382681	SCAP
RNF10	1.11600877	RNF10
TMEM203	1.115228116	TMEM203
COL18A1	1.111440269	COL18A1
MAP4K4	1.100729836	MAP4K4
TSC1	1.08991735	TSC1
IFITM2	1.089344227	IFITM2
CDK19	1.084249596	CDK19
CTSS	1.07294822	CTSS
HDGF	1.072011311	HDGF
PPTC7	1.068669061	PPTC7
CSF1R	1.041806188	CSF1R
RPP25L	1.037642817	RPP25L
HBD	1.016430967	HBD
SLC7A7	0.994851523	SLC7A7

PNP	0.991479072	PNP
DYRK3	0.916365243	DYRK3
HARS2	0.909565449	HARS2
ICA1	0.838890879	ICA1
