# Supplementary material for: Exploration of the Shared Gene Signatures between Myocardium and Blood in Sepsis: Evidence from Bioinformatics Analysis
Source: Biomed Res Int. 2022 Aug 6;2022:3690893. doi: 10.1155/2022/3690893 (PMC9375705; doi:10.1155/2022/3690893)
Supplement: Supplementary Materials — Supplement Figure S1. Correlations between different module memberships generated by weighted correlation network analysis (WGCNA). The gene significances were calculated and are presented in a scatter plot. Correlation coefficients and p-values are shown on the top of the figure. The blue, brown, grey, and turquoise modules were found to be linearly correlated with gene significance. Figure S2. The x-axis of the bar plot indicates the possible clustering method suggested by k-means clustering analysis, and the y-axis presents the evaluation scores of the different clustering methods. The k-means clustering analysis suggests that the best classification is dichotomous. B. The eigenvalue decreases rapidly as the component number increases at the beginning, especially when the first two components are added. The decrease in the eigenvalue was less obvious when more than three components were added. A scree plot shows that two components can well describe the characteristics of the groups clustered by the clustering analysis. Figure S3. Nomograph displaying the risk score of each risk factor when six genes were integrated with the age and appach II score, including SMU1, SP100, and ARHGAP25, which contribute great weight to mortality. Calibration curve in GSE54514, which shows the good fit of our model. The mean absolute error is acceptable (mean absolute error =0.033). Table S1. A total of 1,049 DEGs changed in the same way in both the myocardium and blood datasets, including 549 genes upregulated and 500 genes downregulated. There were 325 genes in the blue module, 116 genes in the brown module, 305 genes in the turquoise module, and 261 genes in the gray module. Table S2. Detailed results of Gene Ontology (GO) analysis and GeneSet Enrichment Analysis (GSEA) of the turquoise and blue modules, as supplementary material for Figure 2. This table displays all the GO analysis pathways, including biological process (BP), cell component (CC), and molecular function (MF), as [file 3690893.f1.zip › Table-S5.pdf]

	s1	id
(Intercep	0.220085	(Intercept)
NNMT	0.016985	NNMT
TNFRSF8	-0.03434	TNFRSF8
TBC1D22B	0.010919	TBC1D22B
DEFA4	6.04E-05	DEFA4
CDT1	0.015442	CDT1
DECR1	-0.00908	DECR1
RPL22L1	0.052045	RPL22L1
ADCY9	-0.00922	ADCY9
HSPA13	0.009489	HSPA13
NR1H2	-0.05685	NR1H2
SMU1	-0.00102	SMU1
NDRG3	-0.02423	NDRG3
PPP1R15A	0.042388	PPP1R15A
MVP	-0.00537	MVP
IL1RL1	0.025254	IL1RL1
CLIC3	-0.00116	CLIC3
ABLIM1	-0.02968	ABLIM1
CYP4V2	-0.00015	CYP4V2
SLC7A5	-0.00828	SLC7A5
SPOP	-0.00582	SPOP
COQ9	-0.019	COQ9
PCYOX1L	-0.01999	PCYOX1L
UBALD2	0.021992	UBALD2
KLRG1	-0.01439	KLRG1
DDHD2	0.026645	DDHD2
TMEM187	-0.02136	TMEM187
ICA1	-0.00169	ICA1
TNS3	-0.00868	TNS3
NIPAL3	-0.0071	NIPAL3
PNP	-0.03362	PNP
THBS1	0.019094	THBS1
ARHGAP25	0.009326	ARHGAP25
LDLR	0.048368	LDLR
ABR	0.036768	ABR
THRA	0.054395	THRA
SP100	-0.03043	SP100
COL18A1	-0.03785	COL18A1
LRRN3	-0.00122	LRRN3
HDGF	-0.00355	HDGF
